# Hidden chemical order in disordered Ba_7_Nb_4_MoO_20_ revealed by resonant X-ray diffraction and solid-state NMR

**DOI:** 10.1038/s41467-023-37802-4

**Published:** 2023-04-24

**Authors:** Yuta Yasui, Masataka Tansho, Kotaro Fujii, Yuichi Sakuda, Atsushi Goto, Shinobu Ohki, Yuuki Mogami, Takahiro Iijima, Shintaro Kobayashi, Shogo Kawaguchi, Keiichi Osaka, Kazutaka Ikeda, Toshiya Otomo, Masatomo Yashima

**Affiliations:** 1 Department of Chemistry, School of Science, Tokyo Institute of Technology, 2-12-1-W4-17, O-okayama, Meguro-ku, Tokyo 152-8551 Japan; 2 NMR Station, National Institute for Materials Science (NIMS), 3-13 Sakura, Tsukuba, Ibaraki 305-0003 Japan; 3 Institute of Arts and Sciences, Yamagata University, 1-4-12 Kojirakawa-machi, Yamagata, Yamagata 990-8560 Japan; 4 Diffraction and Scattering Division, Japan Synchrotron Radiation Research Institute (JASRI), SPring-8, 1-1-1 Kouto, Sayo-cho, Sayo-gun, Hyogo 679-5198 Japan; 5 Industrial Application and Partnership Division, Japan Synchrotron Radiation Research Institute (JASRI), SPring-8, 1-1-1 Kouto, Sayo-cho, Sayo-gun, Hyogo 679-5198 Japan; 6 Institute of Materials Structure Science, High Energy Accelerator Research Organization (KEK), 203-1 Shirakata, Tokai, Ibaraki 319-1106 Japan; 7 J-PARC Center, High Energy Accelerator Research Organization (KEK), 2-4 Shirakata-Shirane, Tokai, Ibaraki 319-1106 Japan; 8 School of High Energy Accelerator Science, The Graduate University for Advanced Studies, 203-1 Shirakata, Tokai, Ibaraki 319-1106 Japan; 9 Graduate School of Science and Engineering, Ibaraki University, 162-1 Shirakata, Tokai, Ibaraki 319-1106 Japan

**Keywords:** Fuel cells, Characterization and analytical techniques, Structure of solids and liquids

## Abstract

The chemical order and disorder of solids have a decisive influence on the material properties. There are numerous materials exhibiting chemical order/disorder of atoms with similar X-ray atomic scattering factors and similar neutron scattering lengths. It is difficult to investigate such order/disorder hidden in the data obtained from conventional diffraction methods. Herein, we quantitatively determined the Mo/Nb order in the high ion conductor Ba_7_Nb_4_MoO_20_ by a technique combining resonant X-ray diffraction, solid-state nuclear magnetic resonance (NMR) and first-principle calculations. NMR provided direct evidence that Mo atoms occupy only the *M*2 site near the intrinsically oxygen-deficient ion-conducting layer. Resonant X-ray diffraction determined the occupancy factors of Mo atoms at the *M*2 and other sites to be 0.50 and 0.00, respectively. These findings provide a basis for the development of ion conductors. This combined technique would open a new avenue for in-depth investigation of the hidden chemical order/disorder in materials.

## Abstract (web summary)

Chemical order/disorder in materials can be difficult to determine for atoms with similar X-ray scattering factors and neutron scattering lengths. Here authors use resonant XRD and NMR to elucidate hidden Mo/Nb chemical order in disordered hexagonal perovskite Ba_7_Nb_4_MoO_20_, with Mo atoms found to be localized near the ion-conducting oxygen deficient layer.

## Introduction

Structural order and disorder have attracted considerable attention because of their correlation with material properties^1–16^. Chemical (occupational) order and disorder have been studied mainly by crystal structure analysis using diffraction data. Such order and disorder are often observed among elements demonstrating similar X-ray atomic scattering factors and similar neutron scattering lengths. Here, we consider the chemical order between two elements *X* and *Y* (*X/Y* order) and define the Scattering Contrast Score of elements *X* and *Y*, SCS(*X*, *Y*) as a measure of the contrasts in X-ray and neutron scattering powers between the *X* and *Y* elements.

1 $${{{{{\rm{SCS}}}}}}\left(X,Y\right)=\left | \frac{N\left(X\right)-N\left(Y\right)}{N\left(X\right)+N\left(Y\right)}\right |+\left | \frac{{{{{{\rm{Re}}}}}}\left[b\left(X\right)\right]-{{{{{\rm{Re}}}}}}\left[b\left(Y\right)\right]}{{{{{{\rm{Re}}}}}}\left[b\left(X\right)\right]+{{{{{\rm{Re}}}}}}\left[b\left(Y\right)\right]}\right | $$

**Fig. 1:**
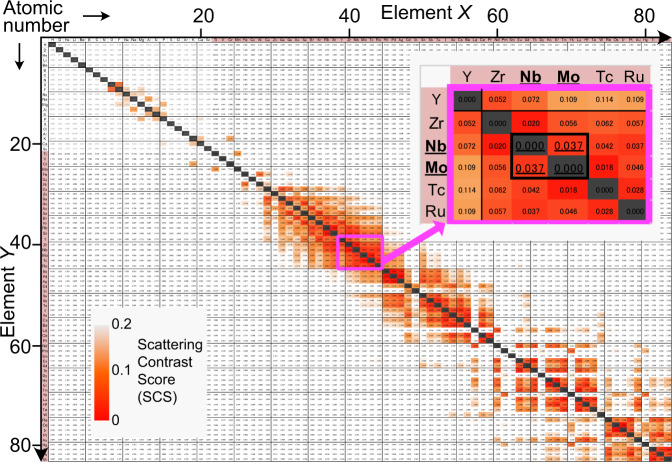
Numerous pairs of *X* and *Y* elements having low scattering contrast score SCS(*X*, *Y*). Each number stands for the SCS(*X*, *Y*) value. Neutron scattering lengths are taken from the NIST website^68^. All the SCS(*X*, *Y*) values are available in Supplementary Data 1 and Supplementary Table 16.

Here *N*(*X*) and Re[*b*(*X*)] are the number of electrons and real part of the coherent neutron scattering length *b* of atom *X*, respectively. There are numerous pairs of *X* and *Y* elements with low SCS values (ex. ~300 *X/Y* pairs with SCS lower than 0.15; red parts in Fig. 1). However, it is difficult to investigate the *X/Y* chemical order hidden in conventional X-ray and neutron diffraction. Thus, the chemical order is an important unresolved issue with numerous materials (Supplementary Table 1). Herein, we propose a technique to elucidate the chemical order, which is a combination of resonant X-ray powder diffraction (RXRD)^17–21^ and solid-state nuclear magnetic resonance (NMR)^22–25^ assisted by density functional theory (DFT) calculations^26–33^. Most materials are polycrystalline or powdered. In contrast to single-crystal X-ray and neutron diffraction, this combined technique can be widely applied to both polycrystalline and powdered samples. Direct evidence of the chemical order can be obtained by NMR^23^; however, it is difficult to quantitatively analyse the chemical order among the constituent elements. In contrast, RXRD enables the quantitative determination of the chemical order by the refinement of occupancy factors, although the refinement results using powder diffraction data are often dependent on the initial structural model. A reliable, quantitative chemical order can be obtained by the present combined technique of NMR and RXRD. We call this combined technique as RXRD/NMR method.

**Fig. 2:**
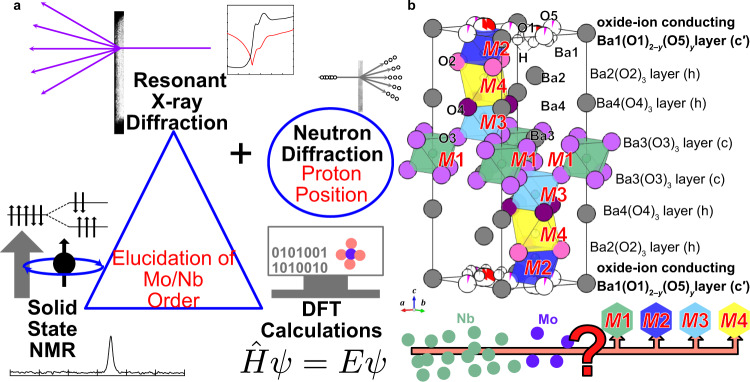
Strategies for the elucidation of the Mo/Nb order and complete crystal structure of Ba_7_Nb_4_MoO_20_·0.15 H_2_O. **a** Combined technique to determine the crystal structure and Mo/Nb order of Ba_7_Nb_4_MoO_20_·0.15 H_2_O. **b** Refined crystal structure showing the *M*1, *M*2, *M*3 and *M*4 sites of Mo and Nb atoms in Ba_7_Nb_4_MoO_20_·0.15 H_2_O where the Mo and Nb atoms are assumed to be completely disordered.

In this study, we aim to elucidate the Mo/Nb order/disorder in a high ion conductor Ba_7_Nb_4_MoO_20_·0.15 H_2_O using the RXRD/NMR method (Fig. 2a). We chose Ba_7_Nb_4_MoO_20_·0.15 H_2_O, because Ba_7_Nb_4_MoO_20_-based oxides and related compounds are emerging materials with high ion conduction, structural disorder and high chemical stability^11,34–40^. The crystal structures of Ba_7_Nb_4_MoO_20_-based oxides have been extensively investigated. However, all the structural refinements were performed assuming the complete Mo/Nb disorder^11,34–36,38,39^, because the Mo^6+^ and Nb^5+^ cations have both (i) the same number of electrons leading to almost the same X-ray atomic scattering factors and (ii) almost the same neutron scattering lengths (6.715 and 7.054 fm for Mo and Nb, respectively). This indicates a small SCS value for the Mo/Nb pair of 0.037. Because the ions migrate in the oxygen-deficient c′ layers of Ba_7_Nb_4_MoO_20_-based oxides^34,36,38,39^, the determination of the chemical order/disorder of Mo and Nb atoms at the crystallographic *M*2 site near the c′ layer is essential (Fig. 2b). Thus, the chemical order of Mo and Nb atoms at the *M*2 site is an important unsolved issue. Herein, we report the chemical order of Mo atoms at the *M*2 site near the c′ layer, which offers unprecedented insight into the understanding of the ion diffusion mechanism in hexagonal perovskite-related oxides.

## Results

A single hexagonal phase of Ba_7_Nb_4_MoO_20_·0.15 H_2_O was prepared by the solid-state reactions (Supplementary Fig. 1). The lattice parameters of Ba_7_Nb_4_MoO_20_·0.15 H_2_O were determined to be *a* = 5.8654(3) and *c* = 16.5390(3) Å using the X-ray diffraction data of the mixture of Ba_7_Nb_4_MoO_20_·0.15 H_2_O sample and internal standard silicon. To determine the occupancy factors of Nb_0.8_Mo_0.2_, Ba and O atoms, preliminary Rietveld analyses of Ba_7_Nb_4_MoO_20_·0.15 H_2_O were performed using neutron diffraction (ND) data and conventional synchrotron X-ray diffraction (SXRD) data recorded with 0.6994806(5) Å X-ray far from the Nb *K*-edge, based on the Mo/Nb disordered model (Supplementary Note 1 and Supplementary Fig. 2 for details). Ba_7_Nb_4_MoO_20_·0.15 H_2_O was confirmed to be a $P\bar{3}m1$ hexagonal perovskite polytype 7H with four Mo/Nb cation sites (*M*1, *M*2, *M*3 and *M*4) (Fig. 2b). The occupancy factors were determined as follows:

2 $$	g ({{{\rm{Nb}}}}; Mi)+g({{{\rm{Mo}}}};Mi)=g ({{{\rm{Ba}}}};{{{\rm{Ba}}}} j)=g({{{\rm{O}}}};{{{\rm{O}}}} k)=1,\\ 	g({{{\rm{Nb}}}}; M 2)+g({{{\rm{Mo}}}};M 2)=0.92,\\ 	g ({{{\rm{Nb}}}}; M 4)+g ({{{\rm{Mo}}}}; M 4)=0.08,\\ 	(i=1 \, {{{\rm{and}}}} \,3; \, j=1,\, 2,\, 3,\, {{{\rm{and}}}} \, 4; k=2,\, 3,\, {{{\rm{and}}}} \, 4)$$

Here, *g*(Nb; *Mi*) + *g*(Mo; *Mi*) = *g*(Nb_0.8_Mo_0.2_; *Mi*), and the *g*(*X*; *s*) denotes the occupancy factor of *X* atoms at *s* site. The refined crystal parameters of Ba_7_Nb_4_MoO_20_·0.15 H_2_O were consistent with those reported in the literature^11,38^.

### Direct experimental evidence for Mo order at *M*2 site by NMR

**Fig. 3:**
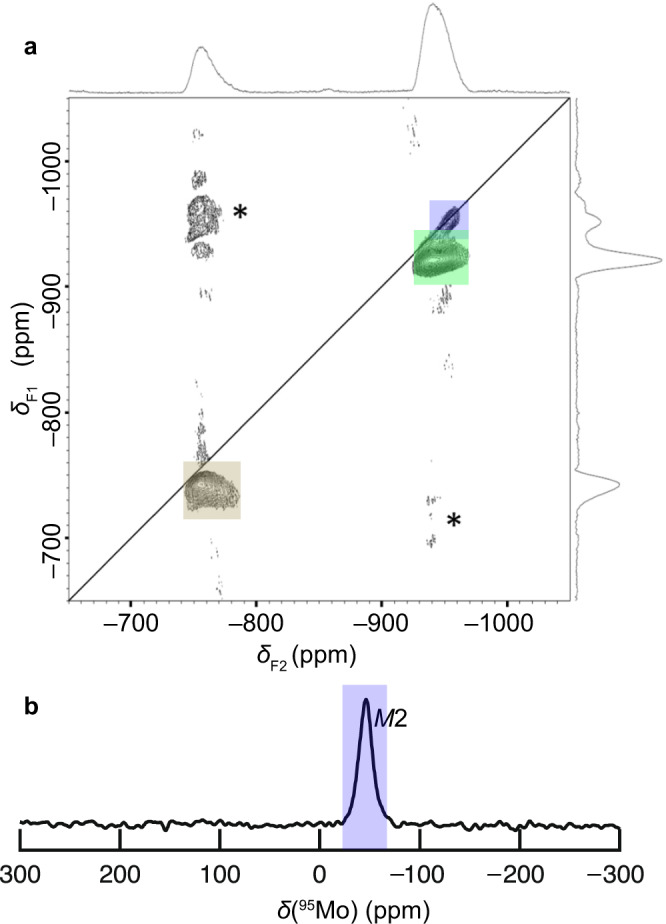
Solid-state NMR spectra of Ba_7_Nb_4_MoO_20_·0.15 H_2_O, showing the site assignment and Mo order. **a** 2D ^93^Nb 3QMAS NMR spectrum and **b** 1D ^95^Mo MAS NMR spectrum of Ba_7_Nb_4_MoO_20_·0.15 H_2_O. An asterisk * denotes spinning sidebands.

We performed ^93^Nb and ^95^Mo solid-state NMR experiments on Ba_7_Nb_4_MoO_20_·0.15 H_2_O at a high magnetic field (18.8 T), which enabled the selective observation of Nb and Mo cations, respectively^23^. Figure 3a and Supplementary Fig. 3 show two-dimensional (2D) ^93^Nb triple-quantum magic angle spinning (3QMAS) and one-dimensional (1D) ^93^Nb magic angle spinning (MAS) NMR spectra, respectively. Three peaks are observed in each ^93^Nb spectrum, indicating the presence of three Nb sites in Ba_7_Nb_4_MoO_20_·0.15 H_2_O. In contrast, in the 1D ^95^Mo MAS NMR spectrum, only one peak is observed (Fig. 3b), indicating a single Mo site and Mo order in Ba_7_Nb_4_MoO_20_·0.15 H_2_O.

To assign the NMR peaks to different crystallographic sites, we performed gauge-including projector augmented wave (GIPAW) DFT calculations of NMR parameters^26–29^ with the VASP programme^41^. To validate this method, the calculated ^93^Nb and ^95^Mo NMR parameters were computed for 13 niobates and 11 molybdates (Supplementary Tables 2, 3). The experimental and calculated ^93^Nb and ^95^Mo NMR parameters show good correlations (Supplementary Fig. 5). Thus, we can assign the Nb and Mo peaks by comparing the experimental and calculated NMR parameters of Ba_7_Nb_4_MoO_20_·0.15 H_2_O. For this purpose, the atomic positions in ten possible structural models with different Nb and Mo configurations were optimised by DFT calculations with the *P*1 space group (Supplementary Figs. 6, 7). The NMR parameters of the optimised structures were estimated by the GIPAW DFT calculations. The calculated peak positions for (Mo2)O_4_ tetrahedron of Ba_7_Nb_4_MoO_20_ ranged from –29 to –36 ppm depending on the structural model, which is close to the experimental peak position of –47 ppm for Ba_7_Nb_4_MoO_20_·0.15 H_2_O. (Table 1), where Mo2 is the Mo atom at the *M*2 site. The calculated quadrupolar coupling constant |*C*_Q_ | values ranged from 0.36 to 0.90 depending on the structural model, which is consistent with the observed value (≤ 2 MHz). In contrast, the peaks calculated for different sites were not observed in the experimental ^95^Mo NMR spectrum. Thus, the single ^95^Mo NMR peak was assigned to the *M*2 site. Similarly, observed ^93^Nb peaks at isotropic chemical shifts *δ*_iso_ = –748, –952 and –928 ppm can be assigned to the *M*1, *M*2 and *M*3 sites, respectively (Supplementary Table 4). These results lead us to conclude that the Mo cations are located at the *M*2 site near the ion-conducting c′ layer, indicating Mo order in Ba_7_Nb_4_MoO_20_·0.15 H_2_O.

**Table 1:** Calculated and experimental ^95^Mo NMR parameters of Ba_7_Nb_4_MoO_20_, showing the peak assignment to the *M*2 site

			DFT-calculated	DFT-calculated	Experimental	Experimental
Models ^a^	Site	Polyhedron ^b^	peak position (ppm)^c^	|*C*_Q_ | (MHz)^d^	peak position (ppm)	|*C*_Q_ | (MHz)^d^
(4), (5)	*M*1	(Mo1)O_6_	+269 ~ +270	1.47, 2.00	not observed	
(1)–(3), (5)	*M*2	(Mo2)O_4_	–29.1 ~ –36.8	0.36 ~ 0.90	–47	≤ 2
(7)–(10)	*M*2	(Mo2)O_5_	–57 ~ +80	5.1 ~ 10.7	not observed	
(6)	*M*3	(Mo3)O_6_	+145	2.87	not observed	
(7), (8)	*M*4	(Mo4)O_6_	+47, +49	5.17, 5.23	not observed	

^a^Each model is shown in Supplementary Figs. 6, 7.

^b^Mo*i* denotes the Mo atom at the *Mi* site.

^c^DFT-calculated peak position under 18.79 T was obtained using the correlation in Supplementary Fig. 5a, including the second-order quadrupolar shift.

^d^Quadrupolar coupling constant |*C*_Q_|. Experimental |*C*_Q_| was estimated from the NMR spectrum (Fig. 3b) with DMFIT^69^.

### Quantitative determination of the occupancy factors of Mo and Nb atoms by resonant X-ray diffraction

**Fig. 4:**
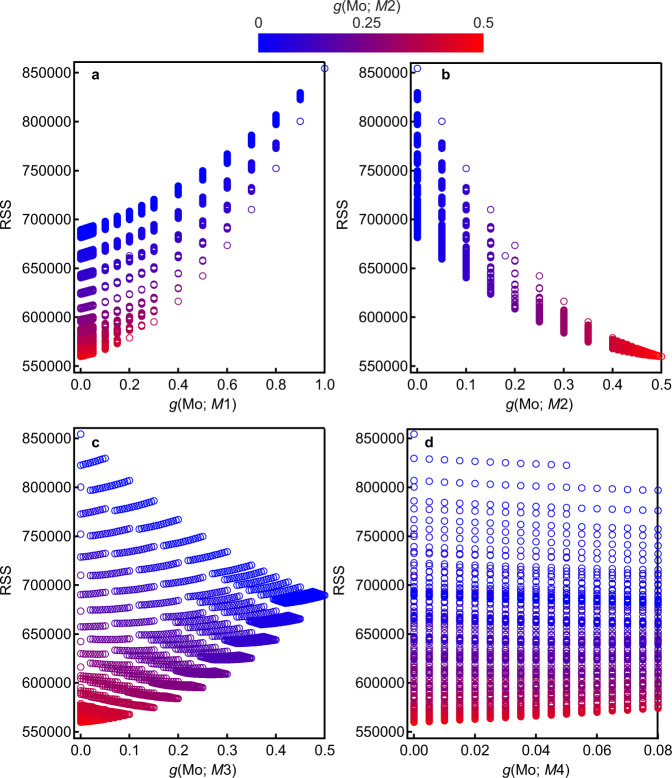
Determination of the Mo occupancy factors in Ba_7_Nb_4_MoO_20_·0.15 H_2_O. Variation of the residual sum of squares (RSS; see the definition of Eq. (3)) with the occupancy factor of Mo atom at the **a** *M*1, **b** *M*2, **c** *M*3 and **d** *M*4 sites in the Rietveld analyses for the RXRD data measured with 0.6527887(5) Å X-ray at the BL02B2 beamline.

We used resonant X-ray diffraction (RXRD) to quantify the occupancy factors of the Mo and Nb atoms in Ba_7_Nb_4_MoO_20_·0.15 H_2_O. We measured the X-ray absorption near edge structure (XANES) spectra of Ba_7_Nb_4_MoO_20_·0.15 H_2_O and the resonant (anomalous) scattering factors of Nb atoms (Supplementary Table 5) were determined by Kramers–Kronig transformation from the XANES spectra^42^ (Supplementary Fig. 8). In the Rietveld analyses of the RXRD data of Ba_7_Nb_4_MoO_20_·0.15 H_2_O, we used the linear constraints Eq. (2), which were obtained in the preliminary analyses of the ND and conventional SXRD data. The occupancy factors of the Nb and Mo atoms at the *M*1, *M*2, *M*3 and *M*4 sites were not simultaneously refined because of strong correlations. Therefore, we carefully examined the residual sum of squares (RSS) in the Rietveld analysis for fixed occupancy values of Mo atoms at the *Mi* site *g*(Mo; *Mi*) step-by-step (0.005 step interval for the finest case). Here the RSS is defined as

3 $${{{{{\rm{RSS}}}}}}=\mathop{\sum }\limits_{i=1}^{N}\frac{1}{{y}_{i}^{{{{{{\rm{obs}}}}}}}}{\left({y}_{i}^{{{{{{\rm{obs}}}}}}}-{y}_{i}^{{{{{{\rm{cal}}}}}}}\right)}^{2}$$

where *N*, ${y}_{i}^{{{{{{\rm{obs}}}}}}}$ and ${y}_{i}^{{{{{{\rm{cal}}}}}}}$ are the total number of intensity data, the observed and calculated intensities for the *i*^th^ step, respectively, of the RXRD data. Figure 4 shows RXRD results obtained with 0.6527887(5) Å X-ray at the BL02B2 beamline of SPring-8, which indicates that the occupancies of Mo atoms are 0.00 at the *M*1, *M*3 and *M*4 sites and 0.50 at the *M*2 site:

4 $$g ({{{\rm{Mo}}}} ; M 1)=g ({{{\rm{Mo}}}}; M 3)=g ({{{\rm{Mo}}}}; M 4)=0.00,\,\, g ({{{\rm{Mo}}}}; M 2)=0.50$$

4′ $${{{\mathrm{Thus}}}} ,\ 	 g({{{\mathrm{Nb}}}}; M1)=g({{{\mathrm{Nb}}}}; M3)=1.00,\\ 	 g({{{\mathrm{Nb}}}}; M2)=0.42,\,\, g({{{\mathrm{Nb}}}}; M4)=0.08$$

The same values were also obtained also in the Rietveld analyses for the RXRD data taken with a 0.6523630(5) Å X-ray at the different beamline BL19B2 of SPring-8 (Supplementary Fig. 9), validating the Mo occupancy values of Eq. (4). In preliminary analyses, the refined occupancy factors *g*(Mo; *Mi*) (*i* = 1, 3 and 4) were negative (Supplementary Tables 6–8), supporting the Mo occupancy factors of Eq. (4). These results clearly indicate the Mo chemical order at the *M*2 site near the ion-conducting c′ layer, which is consistent with the NMR results previously discussed.

### Complete crystal structure of Ba_7_Nb_4_MoO_20_·0.15 H_2_O

**Fig. 5:**
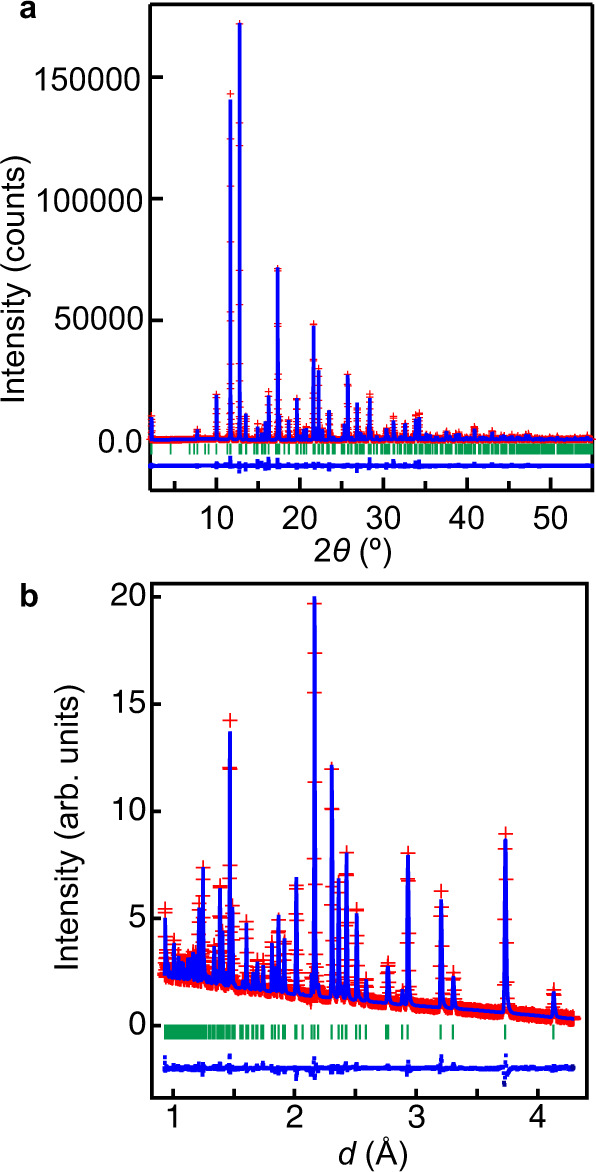
Rietveld fitting patterns of Ba_7_Nb_4_MoO_20_·0.15 H_2_O. **a** Resonant X-ray diffraction (RXRD) data measured at 297 K with 0.6527887(5) Å X-ray at the BL02B2 beamline. **b** Neutron diffraction data at 300 K. The observed and calculated intensities and difference plots are shown by red cross marks, blue solid lines, and blue dots, respectively. Green tick marks denote the calculated Bragg peak positions.

To accurately refine the structural parameters of hydrogen and oxygen atoms, we have analysed the crystal structure of Ba_7_Nb_4_MoO_20_·0.15 H_2_O using neutron diffraction (ND) data collected at 30 and 300 K. During this process, the occupancy factors of Mo and Nb atoms were fixed to the values of Eqs. (4) and (4′), respectively, which were obtained from the analysis of the RXRD data. Excellent fittings were obtained for both ND and RXRD data (Fig. 5 and Supplementary Fig. 10). The crystallographic parameters refined using ND and RXRD data were consistent with each other (Table 2 and Supplementary Table 9). The water content *x* in bulk crystalline Ba_7_Nb_4_MoO_20–*x*_(OH)_2*x*_ (= Ba_7_Nb_4_MoO_20+*x*_H_2*x*_ = Ba_7_Nb_4_MoO_20_·*x* H_2_O) was calculated to be *x* = 0.151(5) using the refined occupancy factors at 30 K (Supplementary Table 10), which is consistent with the water content estimated from the thermogravimetric-mass spectroscopic (TG-MS) analyses (Supplementary Fig. 11). The O1–H distance was estimated to be 1.07(4) Å using the refined crystal structure of Ba_7_Nb_4_MoO_20_·*x* H_2_O at 300 K, which agrees with the O–H distance of 0.99738(8) Å obtained from its Raman spectrum (Supplementary Fig. 12), indicating the presence of hydroxide ions formed by the hydration. The bond-valence sums (BVSs) of cations and anions for the refined structure of Ba_7_Nb_4_MoO_20_·0.15 H_2_O agree with their formal charges (Table 2). These results confirm the validity of the refined crystal structure of Ba_7_Nb_4_MoO_20_·0.15 H_2_O.

**Table 2:** Refined crystal parameters and reliability factors in Rietveld analysis of the neutron diffraction data of Ba_7_Nb_4_MoO_19.849_(OH)_0.302_ (= Ba_7_Nb_4_MoH_0.302_O_20.151_ = Ba_7_Nb_4_MoO_20.151_H_0.302_ = Ba_7_Nb_4_MoO_20_·0.151 H_2_O) at 300 K

Site / Atom label	Atom	Wyckoff position	*g*^f^	*x*	*y*	*z*	*U*_iso_ (Å^2^)^g^	BVS^d^
Ba1	Ba	1*a*	1^e^	0	0	0	0.0165(7)	2.02
Ba2	Ba	2*d*	1^e^	1/3	2/3	0.82374(7)	0.0138(3)	2.19
Ba3	Ba	2*d*	1^e^	1/3	2/3	0.57420(9)	0.0091(3)	2.29
Ba4	Ba	2*c*	1^e^	0	0	0.27870(8)	0.0078(4)	1.91
*M*1	Nb	1*b*	1^e^	0	0	1/2	0.0060(3)	4.57
*M*2	Nb	2*d*	0.42	1/3	2/3	0.09489(6)	0.0060(3)	4.91
*M*2	Mo	2*d*	0.5	1/3	2/3	0.09489(6)	0.0060(3)	5.54
*M*3	Nb	2*d*	1^e^	1/3	2/3	0.34909(6)	0.0060(3)	4.61
*M*4	Nb	2*d*	0.08	1/3	2/3	0.1926^a^	0.0060(3)	3.65
O1	O	6*i*	1/3	0.3532(4)	0.7064(9)	–0.01209(6)	0.0196(8)	1.84
O2	O	6*i*	1^e^	0.16652(14)	0.3330(2)	0.13082(4)	0.01167(16)	1.94
O3	O	6*i*	1^e^	0.16323(14)	0.3265(2)	0.43098(4)	0.01086(18)	1.95
O4	O	6*i*	1^e^	0.49502(9)	0.50498(9)	0.29455(3)	0.00791(18)	1.98
O5	O	3*e*	0.0504^b^	1/2	0	0	0.0196(8)	1.28
H	H	12*j*	0.0252^b^	0.346(4)	0.500(5)	0.9748(16)	0.041^c^	0.85

Crystal system: trigonal. Space group: $P\bar{3}m1$ (No.164, setting 1). Lattice parameters: *a* = *b* = 5.865653(4) Å, *c* = 16.53699(3) Å. The number of formula per unit cell: *Z* = 1. Reliability factors: *R*_wp_ = 2.719%, *R*_p_ = 2.254%, *R*_B_ = 4.577%, *R*_*F*_ = 3.872%, GoF = 19.857.

^a^*z* coordinate of the Nb4 atom was fixed to those from preliminary analyses.

^b^Occupancy factors of O5 and H atoms were fixed to those from ND analysis at 30 K.

^c^Atomic displacement parameter of the H atom was fixed to those from preliminary analyses.

^d^BVS bond-valence sums. Here the bond-valence parameters after ref. ^70^ were used for the calculations of BVSs. The low BVS values of O5 and H atoms than the formal charges of –2 and +1 are consistent with the low occupancy values of 0.0504 and 0.0252, respectively.

^e^The occupancy factors of Ba1–Ba4, *M*1, *M*3, and O2–O4 atoms were fixed to unity, because the refined values agreed with unity within three times of estimated standard deviations (see Supplementary Note 1 for details).

^f^*g* = *g*(*X*; *s*): Occupancy factor of *X* atom at the *s* site. *g*(Ba; Ba1) = *g*(Ba; Ba2) = *g*(Ba; Ba3) = *g*(Ba; Ba4) = *g*(Nb; *M*1) = *g*(Nb; *M*3) = *g*(O; O2) = *g*(O; O3) = *g*(O; O4) = 1; *g*(Nb; *M*2) = 0.42, *g*(Mo; *M*2) = 0.5, *g*(Nb; *M*4) = 0.08; *g*(O; O1) = 1/3, *g*(O; O5) = 0.0504; *g*(H; H) = 0.0252. *x*, *y*, and *z*: atomic coordinates.

^g^*U*_iso_(*Xn*) Isotropic atomic displacement parameter of *X* atom at the *Xn* site. Linear constraints in the Rietveld analysis: *U*_iso_(Nb1) = *U*_iso_(Nb2) = *U*_iso_(Mo2) = *U*_iso_(Nb3) = *U*_iso_(Nb4).

**Fig. 6:**
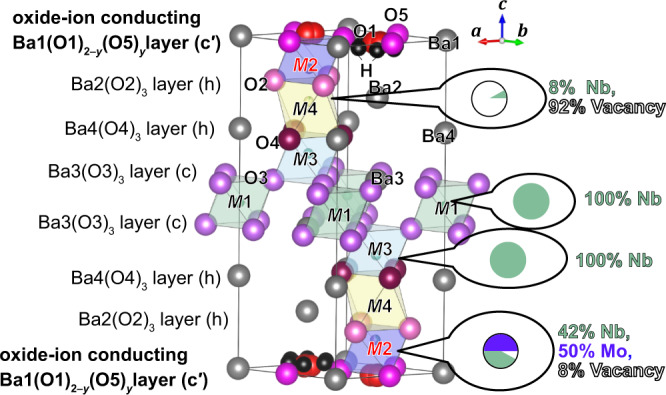
Crystal structure of Ba_7_Nb_4_MoO_19.849_(OH)_0.302_. Refined crystal structure of Ba_7_Nb_4_MoO_19.849_(OH)_0.302_ at 300 K, which shows the Mo chemical order and site occupancies of Nb and Mo atoms.

Figure 6 shows the refined crystal structure of Ba_7_Nb_4_MoO_20_·0.15 H_2_O, with the sequence c′hhcchh. Oxygen-deficient lattice O1 and interstitial O5 sites exist in the c′ layer. At high temperatures, oxide ions can migrate via O1–O5 diffusion pathways and the interstitialcy diffusion mechanism as shown by the maximum-entropy method (MEM) neutron scattering length density (NSLD) distribution of Ba_7_Nb_3.9_Mo_1.1_O_20.05_ at 1073 K^36^. Similar O1–O5 paths were visualised in MEM NSLD distribution of wet Ba_7_Nb_4_MoO_20_·0.87 H_2_O at 368 K^34^.

Structural disorders have been reported in Ba_7_Nb_4_MoO_20_-based materials^11,34,36,38,39^. In contrast, a striking feature is the presence of Mo atoms only at the *M*2 site near the ion-conducting c′ layer, indicating Mo chemical order. DFT-optimised structures with Mo order at the *M*2 site have slightly lower energies than those with Mo disorder and Mo atoms at *M*1 and *M*3 sites, which supports the Mo chemical order at the *M*2 site (Supplementary Table 11). This is the first report on the chemical order of Mo atoms in Ba_7_Nb_4_MoO_20_-based materials. In the literature^11,34–36,38,39^, all structural analyses were performed based on complete Mo/Nb disorder. Meanwhile, in this study, the Mo order was indicated not only by structural refinements using RXRD data but also by NMR measurements and DFT calculations. An important question is why the Mo order occurs. The probable explanation is as follows: the *M*2 site has a smaller space compared with other *Mi* sites (*i* = 1, 3 and 4) (Supplementary Table 12), and the size of the Mo cation is smaller than that of the Nb cation; thus, Mo order occurs. Indeed, the BVS of Mo at *M*2 site 5.54 agrees with the formal charge 6 of Mo^6+^, which is higher than the BVS values of Mo atoms at *M*1 (4.63), at *M*3 (4.76) and at *M*4 (3.51) sites indicating the underbonding and instability of Mo atoms at *Mi* sites (*i* = 1, 3 and 4).

## Discussion

The present work has demonstrated the chemical order of Mo atoms at the *M*2 site near the ion-conducting c′ layer in Ba_7_Nb_4_MoO_20_·0.15 H_2_O by the combined technique of solid-state NMR, resonant XRD and DFT calculations, in addition to the neutron diffraction and conventional SXRD. The NMR spectra provided direct experimental evidence for the Mo order, while the structural analyses using the RXRD data enabled the quantitative determination of the occupancy factors of Mo and Nb atoms. This combined technique can be used to investigate the hidden chemical order in various ion-conducting hexagonal perovskite derivatives such as Ba_7_Nb_4−*x*_Mo_1+*x*_O_20+*x*/2_^36^, Ba_7_Nb_4−*x*_W_*x*_MoO_20+*x*/2_^38^, Ba_7_Nb_4−*x*_Cr_*x*_MoO_20+*x*/2_^39^ and Ba_3_MoNbO_8.5_^6,43,44^ where the Mo occupancies at the *Mi* sites (*i* = 1, 2, 3 and 4) are unknown. Here *x* is the dopant or excess Mo content. Beyond the limits of the combined technique of conventional X-ray diffraction and NMR (’SMARTER’ crystallography^45,46^), this RXRD/NMR method can be applied to numerous compounds such as thermoelectric Ag_1–*x*_Cd_*x*_SbTe_2_^16^ and superconducting Zr_5_Ir_2_Os^47^ exhibiting chemical order/disorder of atoms with both similar X-ray atomic scattering factors and similar neutron scattering lengths (Fig. 1 and Supplementary Table 1). In contrast to the single-crystal X-ray diffraction^19,48^ and X-ray fluorescence holography^49^, the RXRD/NMR method uses powders or polycrystalline samples, making it versatile and easily applicable to in situ measurements (e.g., at high temperatures). The combined technique would be useful for investigating not only the periodic average structure but also the short- and intermediate-range order/disorder hidden in conventional diffraction and total scattering.

Next, we discuss the influences of Mo chemical order on the material properties of Ba_7_Nb_4_MoO_20_. The flexibility of the coordination of *M*2 atoms near the c′ layer was suggested to determine the high ion conduction in Ba_7_Nb_4_MoO_20_·0.5 H_2_O from the ab initio molecular dynamics simulations^34^. Since the present work has indicated that Mo cations are localised at the *M*2 site near the ion-conducting c′ layer in Ba_7_Nb_4_MoO_20_·0.15 H_2_O, the flexibility of Mo atoms is important for the high ion conduction in Ba_7_Nb_4_MoO_20_-based materials as well as in other Mo-containing ionic conductors such as La_2_Mo_2_O_9_^50^. Therefore, the bulk conductivity of Ba_7_Nb_4−*x*_Mo_1+*x*_O_20+*x*/2_ increases with increasing the excess amount of Mo atoms *x* from *x* = 0 to 0.1^36^, which is ascribed to not only a larger amount of excess oxygen atoms but also the larger amount of Mo atoms.

The energy barriers for oxide-ion migration *E*_b/O_ of Mo-ordered and virtual Mo-disordered Ba_7_Nb_4_MoO_20_·0.15 H_2_O were calculated using the bond-valence method^51,52^. The *E*_b/O_ along the *c* axis in Mo-ordered Ba_7_Nb_4_MoO_20_·0.15 H_2_O (1.93 eV) is higher than that in the virtual Mo-disordered Ba_7_Nb_4_MoO_20_·0.15 H_2_O (1.60 eV) [Supplementary Table 13], which is attributable to the narrower bottleneck for oxide-ion migration along the *c* axis due to the higher occupancy factor of larger Nb cations at the bottleneck triangle (Supplementary Fig. 14).

The substitution of Nb with Mo improves the oxide-ion conductivity because of the larger number of interstitial oxygen atoms (higher carrier concentration). The formation energies Δ*H*_f_ of the Mo-ordered and virtual Mo-disordered Ba_7_Nb_3.5_Mo_1.5_O_20.25_ oxides were calculated using the DFT method. The calculated Δ*H*_f_ values of Mo-ordered models are lower than those of virtual Mo-disordered ones (Supplementary Table 15), which indicates that Mo ordering stabilises Ba_7_Nb_3.5_Mo_1.5_O_20.25_ with interstitial oxygen atoms more efficiently than Mo disordering, leading to higher oxide-ion conductivity. The hydration enthalpies Δ*H*_hyd_ of Mo-ordered and Mo-disordered Ba_7_Nb_4_MoO_20_ were also investigated by DFT calculations, because the hydration is important for proton conduction in Ba_7_Nb_4_MoO_20_. Compared with the calculated Δ*H*_hyd_ of the Mo-disordered Ba_7_Nb_4_MoO_20_ (1.70 kJ mol^−1^), that of Mo-ordered Ba_7_Nb_4_MoO_20_ (−22.7 kJ mol^−1^) is close to the experimental value below 300 °C (−24 kJ mol^−1^)^34^. The calculated Δ*H*_hyd_ for the Mo-ordered system (−22.7 kJ mol^−1^) is lower than that of the Mo-disordered one (1.70 kJ mol^−1^), indicating that the Mo ordering also stabilises the hydrated Ba_7_Nb_4_MoO_20_ more efficiently compared with Mo disordering. These results demonstrate that the Mo order in Ba_7_Nb_4_MoO_20_ affects the material properties. The present findings represent a major advance in the fundamental understanding of the correlation between the crystal structure and material properties of ionic conductors.

## Methods

### Synthesis and characterisation

The Ba_7_Nb_4_MoO_20_·0.15 H_2_O samples were prepared by the solid-state reaction method. High-purity (>99.9%) BaCO_3_, Nb_2_O_5_ and MoO_3_ were mixed as ethanol slurries and ground as dry powders using an agate mortar and pestle. The obtained powders were calcined at 900 °C for 12 h for decarbonation. The materials thus obtained were crushed and ground into fine powders in an agate mortar for 1 h as dried powders and ethanol slurries. The resultant powders were uniaxially pressed at 150 MPa and then sintered in air at 1100 °C for 24 h. The sintered pellets were crushed and ground into fine powders for X-ray powder diffraction (XRD), inductively coupled plasma atomic emission spectroscopy (ICP-AES, Shimadzu ICPS-8100 spectrometer), and TG-MS measurements. The ICP-AES results indicated that the cation molar ratio of Ba_7_Nb_4_MoO_20_·0.15 H_2_O was Ba: Nb: Mo = 6.89(12): 4.078(18): 1.034(10), which is consistent with the nominal composition. TG-MS analyses of Ba_7_Nb_4_MoO_20_·0.15 H_2_O were performed using RIGAKU Thermo Mass Photo under He flows at a heating rate of 20 K min^–1^ up to 900 °C. The Raman spectrum of Ba_7_Nb_4_MoO_20_·0.15 H_2_O was collected using an NRS-4100 (JASCO Co.) instrument with an excitation wavelength of 532 nm.

### Synchrotron X-ray and neutron diffraction experiments and data analysis

Synchrotron X-ray diffraction (SXRD) experiments were performed at beamlines BL02B2 (297 K)^53^ and BL19B2 (300 K)^54^ of SPring-8. X-ray wavelengths for resonant X-ray diffraction experiments were selected from the spectrum of Nb *K*-edge X-ray absorption near edge structure (XANES) for a Ba_7_Nb_4_MoO_20_·0.15 H_2_O powder. X-ray wavelengths were determined from the X-ray diffraction data of standard silicon powder (SRM 640c) using FullProf software^55^. Conventional SXRD data were recorded with a 0.6994806(5) Å X-ray. RXRD measurements were performed using a 0.6527887(5) Å X-ray at the BL02B2 beamline and a 0.6523630(5) Å X-ray at BL19B2. Both the conventional SXRD and RXRD data were analysed using the Rietveld method with the computer programme RIETAN-FP^56^. We used atomic scattering factors in the form of *f* = *f*_0_ + *f’*′ + *if”*, where *f*_0_ is the Thomson scattering factor and *f’* and *f”* are the resonant (anomalous) scattering factors. The *f’* and *f”* factors of the Nb atom were calculated from the XANES spectrum (Supplementary Fig. 8) recorded at BL19B2 with the programme DiffKK^57^, and the *f’* and *f”* factors of Ba, Mo and O atoms were obtained from the theoretical values reported by Cromer and Libermann^58^ (Supplementary Table 5).

Time-of-flight (TOF) neutron diffraction data of Ba_7_Nb_4_MoO_20_·0.15 H_2_O were obtained at 30 and 300 K using a high-intensity total diffractometer NOVA (BL-21) in the MLF of J-PARC. Rietveld analyses were performed using Z-Rietveld^59,60^ using neutron diffraction data obtained from the backscattering bank of the NOVA.

The bond-valence-based energy (BVE) landscapes for a test oxide ion and proton in Ba_7_Nb_4_MoO_20_·0.15 H_2_O were calculated using refined crystal parameters at 300 K using the SoftBV programme^51,52^. The refined structures and BVE landscapes were depicted using the *VESTA 3*^61^.

### Solid-state NMR experiments

NMR experiments of Ba_7_Nb_4_MoO_20_·0.15 H_2_O were performed with a 3.2-mm homemade MAS probe at a spinning speed of 20 kHz under a magnetic field of 18.79 T, corresponding to ^95^Mo and ^93^Nb Larmor frequencies of 52.16 and 195.84 MHz, respectively. 1D ^95^Mo and 2D ^93^Nb NMR spectra were recorded using a JEOL JNM-ECA 800 spectrometer, whereas 1D ^93^Nb NMR spectra were obtained using a JEOL JNM-ECZ 800 R spectrometer. ^95^Mo chemical shifts were referenced to 2.0 M aqueous solution of Na_2_MoO_4_ at 0.00 ppm (refs. ^62,63^), and ^93^Nb chemical shifts were externally referenced to NaNbO_3_ at −1093 ppm (ref. ^64^). ^95^Mo NMR spectra of α-MoO_3_ and BaMoO_4_ were also obtained to investigate the relationships between the experimental and DFT-calculated NMR parameters (Supplementary Figs. 4, 5). The 1D ^95^Mo NMR spectra were acquired by accumulating 22,000 scans using a 1.2 μs single-pulse sequence with a relaxation delay of 20 s. The 1D ^93^Nb spectra were measured using a spin-echo sequence (2.0 and 4.0 μs), accumulating 1024 scans with a relaxation delay of 1 s. The 2D ^93^Nb 3QMAS NMR spectra were measured by the conventional three-pulse sequence with z-filter^65^ (2.0, 0.9 and 15 μs), and recorded with 264 transients averaged for each of the 1024 *t*1 points with a relaxation delay of 0.2 s. Shearing transformation^66^ was applied to the spectra. Here, the centre of the F1 axis was set to the centre of the F2 axis.

### Density functional theory (DFT)-based calculations

Generalised gradient approximation (GGA) electronic calculations were performed using Vienna Ab initio Simulation Package (VASP)^41^. We used projector augmented wave (PAW) potentials for Ba, Nb, Mo and O atoms, plane-wave basis sets with a cutoff of 500 eV and the Perdew–Burke–Ernzerhof (PBE) GGA functionals. The crystal parameters refined using the neutron diffraction data of Ba_7_Nb_4_MoO_20_·0.15 H_2_O at 300 K were used as the initial parameters in the DFT structural optimisations. Atomic coordinates of Ba_7_Nb_4_MoO_20_ were optimised in the space group *P*1, with the convergence condition of 0.02 eV Å^–1^. The supercell programme^67^ was used to generate supercell models. The formation energies of Ba_7_Nb_3.5_Mo_1.5_O_20.25_ $\Delta {H}_{{{{{{\rm{f}}}}}}}$ for the Mo-ordered and virtual Mo-disordered models were calculated according to the following equation:

$${{{{{{\rm{Ba}}}}}}}_{7}{{{{{{\rm{Nb}}}}}}}_{4}{{{{{{\rm{MoO}}}}}}}_{20}+1/2{{{{{{\rm{MoO}}}}}}}_{3}\to {{{{{{\rm{Ba}}}}}}}_{7}{{{{{{\rm{Nb}}}}}}}_{3.5}{{{{{{\rm{Mo}}}}}}}_{1.5}{{{{{{\rm{O}}}}}}}_{20.25}+1/4{{{{{{\rm{Nb}}}}}}}_{2}{{{{{{\rm{O}}}}}}}_{5}$$

The optimised structures are shown in Supplementary Fig. 15. The hydration enthalpies $\Delta {H}_{{{{{{\rm{hyd}}}}}}}$ were also estimated for the Mo-ordered and virtual Mo-disordered models (Supplementary Fig. 16) according to the following reaction:

$${\left({{{{{{\rm{Ba}}}}}}}_{7}{{{{{{\rm{Nb}}}}}}}_{4}{{{{{{\rm{MoO}}}}}}}_{20}\right)}_{4}+{{{{{{\rm{H}}}}}}}_{2}{{{{{\rm{O}}}}}}\to {\left({{{{{{\rm{Ba}}}}}}}_{7}{{{{{{\rm{Nb}}}}}}}_{4}{{{{{{\rm{Mo}}}}}}}{{{{{{\rm{O}}}}}}}_{20.25}{{{{{{\rm{H}}}}}}}_{0.5}\right)}_{4}$$

DFT calculations of the ^93^Nb and ^95^Mo chemical magnetic shielding and electric field gradient tensors were performed using the VASP code with a cutoff energy of 700 eV for the plane-wave basis sets, where the total energy converged within 10^–8^ eV/atom. The GIPAW formalism^28^ was utilised for the calculations of the NMR chemical shielding tensors.

## Supplementary information

Supplementary Information

Peer Review File

Description of Additional Supplementary Files

Supplementary Data 1

## Data Availability

The datasets generated during and/or analysed during the current study are available from the corresponding author on request.
