# Author Correction: Hidden chemical order in disordered Ba_7_Nb_4_MoO_20_ revealed by resonant X-ray diffraction and solid-state NMR

**DOI:** 10.1038/s41467-023-40292-z

**Published:** 2023-07-26

**Authors:** Yuta Yasui, Masataka Tansho, Kotaro Fujii, Yuichi Sakuda, Atsushi Goto, Shinobu Ohki, Yuuki Mogami, Takahiro Iijima, Shintaro Kobayashi, Shogo Kawaguchi, Keiichi Osaka, Kazutaka Ikeda, Toshiya Otomo, Masatomo Yashima

**Affiliations:** 1 Department of Chemistry, School of Science, Tokyo Institute of Technology, 2-12-1-W4-17, O-okayama, Meguro-ku, Tokyo 152-8551 Japan; 2 NMR Station, National Institute for Materials Science (NIMS), 3-13 Sakura, Tsukuba, Ibaraki 305-0003 Japan; 3 Institute of Arts and Sciences, Yamagata University, 1-4-12 Kojirakawa-machi, Yamagata, Yamagata 990-8560 Japan; 4 Diffraction and Scattering Division, Japan Synchrotron Radiation Research Institute (JASRI), SPring-8, 1-1-1 Kouto, Sayo-cho, Sayo-gun, Hyogo 679-5198 Japan; 5 Industrial Application and Partnership Division, Japan Synchrotron Radiation Research Institute (JASRI), SPring-8, 1-1-1 Kouto, Sayo-cho, Sayo-gun, Hyogo 679-5198 Japan; 6 Institute of Materials Structure Science, High Energy Accelerator Research Organization (KEK), 203-1 Shirakata, Tokai, Ibaraki 319-1106 Japan; 7 J-PARC Center, High Energy Accelerator Research Organization (KEK), 2-4 Shirakata-Shirane, Tokai, Ibaraki 319-1106 Japan; 8 School of High Energy Accelerator Science, The Graduate University for Advanced Studies, 203-1 Shirakata, Tokai, Ibaraki 319-1106 Japan; 9 Graduate School of Science and Engineering, Ibaraki University, 162-1 Shirakata, Tokai, Ibaraki 319-1106 Japan

**Keywords:** Fuel cells, Characterization and analytical techniques, Structure of solids and liquids

Correction to: *Nature Communications* 10.1038/s41467-023-37802-4, published online 24 April 2023

The original version of this Article contained errors in Table 1, where: the second, third, fourth, fifth and sixth entries of the fourth column (DFT-calculated peak position (ppm)) were incorrectly given as ‘+261 ~ +291’, ‘–29.1 ~ –36.0’, ‘+135 ~ +601’, ‘+192.2’ and ‘+208, +202’ instead of the correct values ‘+269 ~ +270’, ‘–29.1 ~ –36.8’, ‘–57 ~ +80’, ‘+145’ and ‘+47, +49’; the third and fourth entry of the fifth column (DFT-calculated |C_Q_| (MHz)) were incorrectly given as ‘0.36–0.90’ and ‘5.1–10.7’ instead of the correct values ‘0.36 ~ 0.90’ and ‘5.1 ~ 10.7’. This has been corrected in both the PDF and HTML versions of the Article.

The original version of the Supplementary Information associated with this Article contained errors in the legend of Supplementary Figure 7 and in Supplementary Table 4. In the legend of Supplementary Figure 7 the figure panels were incorrectly labelled as ‘**a,b** Nb’ and ‘**c,d** Mo’, rather than the correct ‘**a,b** Mo’ and ‘**c,d** Nb’. In Supplementary Table 4, the second and fourth entry of the fourth column (DFT-calculated δ_iso_ (ppm)) were incorrectly given as ‘–848 ~ –890’ and ‘–867 ~ –898’ instead of the correct values ‘–848 ~ –862’ and ‘–867 ~ –897’; the first and fourth entry of the fifth column (|P_Q_| (MHz)) were incorrectly given as ‘18 ~ 27’ and ‘41 ~ 68’ instead of the correct values ‘18 ~ 20’ and ‘41 ~ 50’. The HTML has been updated to include a corrected version of the Supplementary Information.

## Supplementary information

Updated Supplementary Information

