# Supplementary material for: Hidden chemical order in disordered Ba7Nb4MoO20 revealed by resonant X-ray diffraction and solid-state NMR
Source: Nat Commun. 2023 Apr 24;14:2337. doi: 10.1038/s41467-023-37802-4 (PMC10126145; doi:10.1038/s41467-023-37802-4)
Supplement: Supplementary file 1 — Supplementary Information [file 41467_2023_37802_MOESM1_ESM.pdf]

Supplementary information

Hidden chemical order in disordered $Ba_7Nb_4MoO_{20}$ revealed by resonant X-ray diffraction and solid-state NMR

Yuta Yasui et al.

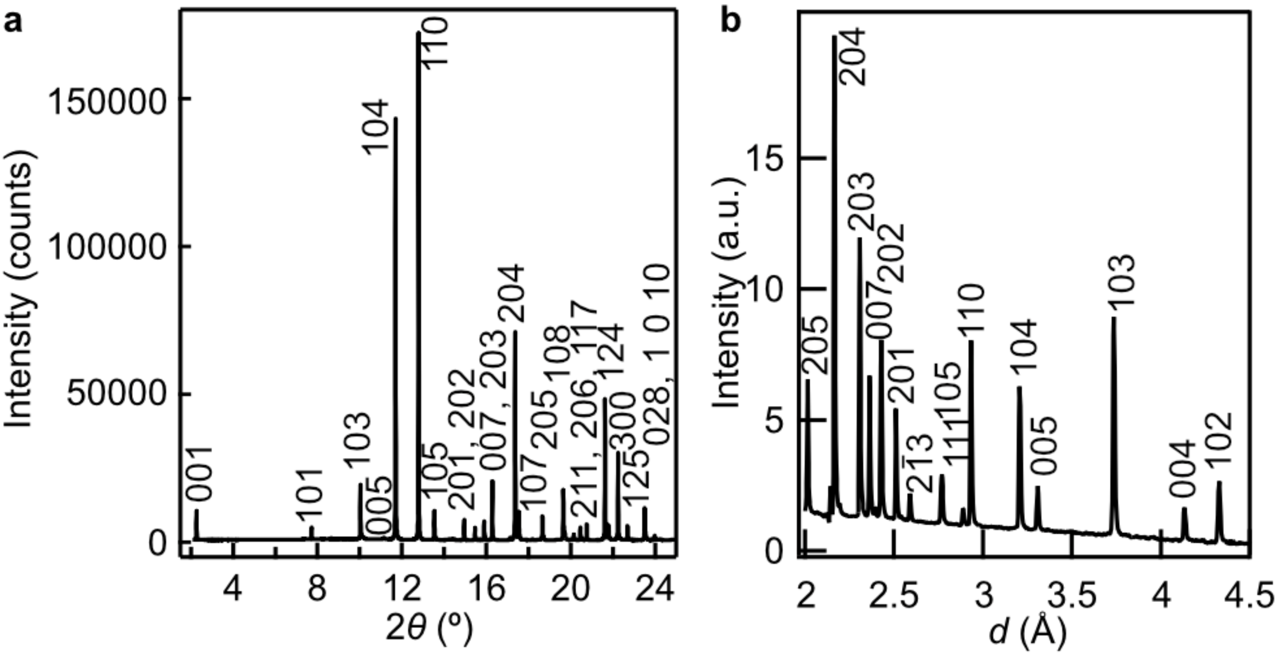

Supplementary Figure 1. Powder diffraction patterns of $Ba_7Nb_4MoO_{20}\cdot 0.15\ H_2O$. a Resonant synchrotron X-ray diffraction pattern of $Ba_7Nb_4MoO_{20}\cdot 0.15\ H_2O$ taken at 297 K at the beamline BL02B2 of SPring-8 (Wavelength of X-ray: 0.6523630(5) Å). b Neutron diffraction pattern of $Ba_7Nb_4MoO_{20}$ measured at 300 K. Each number *hkl* denotes the reflection index for the hexagonal $Ba_7Nb_4MoO_{20}$.

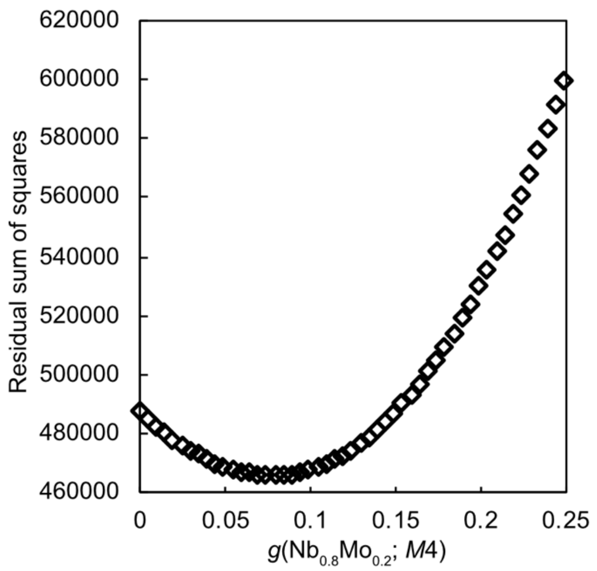

Supplementary Figure 2. Variation of the residual sum of squares (RSS; see the definition of Eq. (3) in the text) with the fixed occupancy factor of $Nb_{0.8}Mo_{0.2}$ atom at the *M*4 site *g*($Nb_{0.8}Mo_{0.2}$; *M*4) in the Rietveld analyses of conventional SXRD data measured at 297 K with 0.6994806(5) Å X-ray at the beamline BL02B2. *g*($Nb_{0.8}Mo_{0.2}$; *M*4) was estimated to be 0.08.

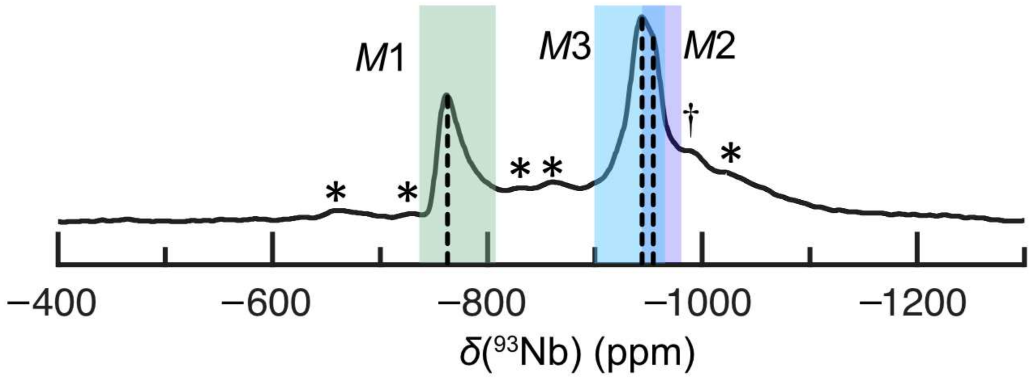

Supplementary Figure 3. 1D ^{93}Nb MAS NMR spectrum of $Ba_7Nb_4MoO_{20}\cdot 0.15\ H_2O$. Black dashed lines are eye guides for the NMR peak positions. Each asterisk * denotes a spinning sideband. Dagger † stands for a peak, which could be assigned to $(Nb2)O_5$ or $(Nb4)O_6$.

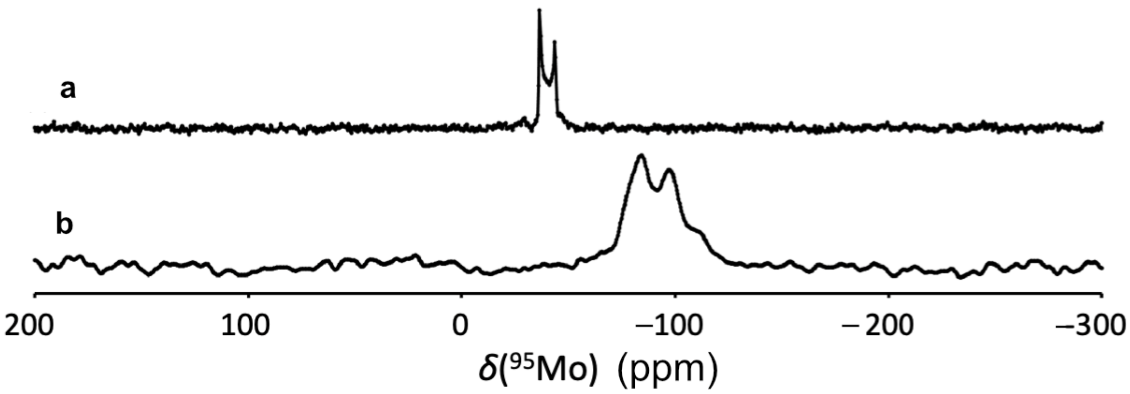

Supplementary Figure 4. ^{95}Mo MAS NMR spectra of a $BaMoO_4$ and b α-MoO_3 measured for comparison with $Ba_7Nb_4MoO_{20}$·0.15 H_2O. The chemical shift δ_{iso}, quadrupolar coupling constant C_Q and asymmetry parameter η of $BaMoO_4$ and α-MoO_3 were obtained by DMFIT software[1]. The estimated values were δ_{iso} = –35 ppm, C_Q = 1.68 MHz and η = 0.1 for $BaMoO_4$ and δ_{iso} = –73 ppm, C_Q = 2.8 MHz and η = 0.3 for α-MoO_3, which are consistent with literature,[2–6] and used for the plots in the Supplementary Figs. 5a, 5b and 5c.

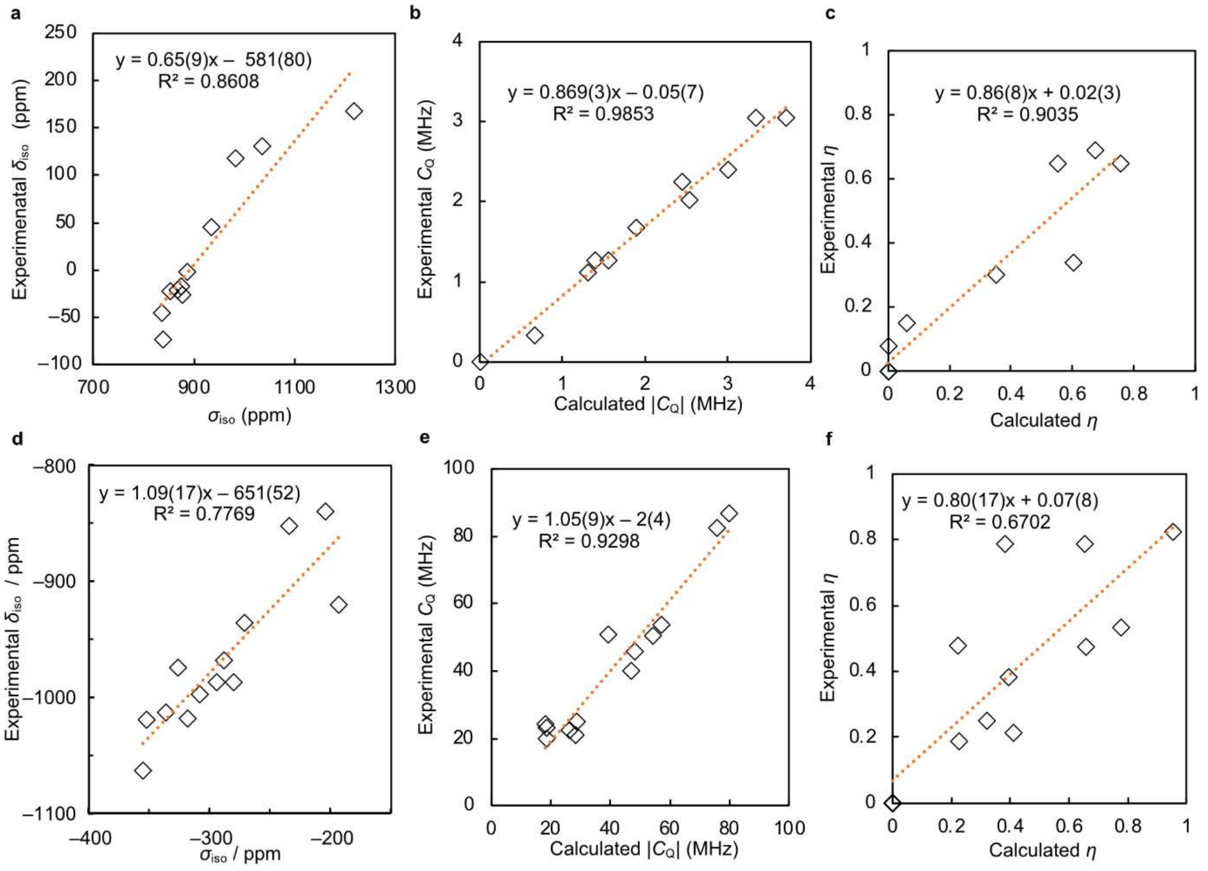

Supplementary Figure 5. Correlation between experimental and calculated NMR parameters for a-c ^{95}Mo and d-f ^{93}Nb nuclei. The calculated NMR parameters were obtained by GIPAW DFT calculations with VASP. Here δ_{iso} is experimental isotropic chemical shift, and σ_{iso} is magnetic shielding obtained by GIPAW DFT[7–10] calculations with VASP[11], C_Q is the quadrupolar coupling constant, and η is the asymmetry parameter. R is the correlation coefficient. The orange fitted dotted lines for (b, c, e), (a, d) and f indicate very good, good and modest correlations, respectively, between the experimental and calculated parameters. The details of the data are shown in Supplementary Tables 2 and 3.

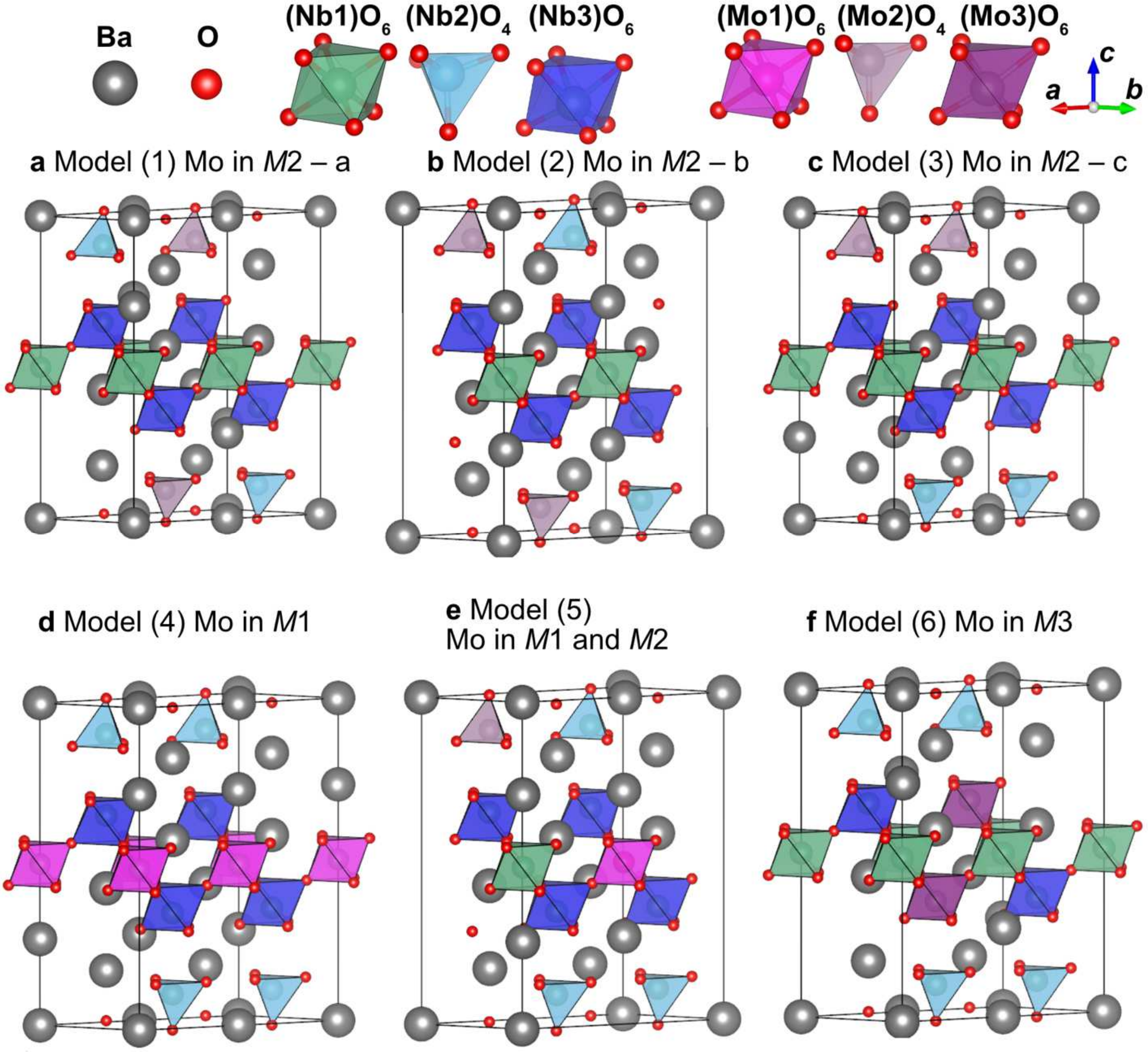

Supplementary Figure 6. Optimized structures for the structural models (1)-(6) with different configurations of Nb and Mo atoms in the 2×1×1 cell of $Ba_7Nb_4MoO_{20}$ used for the total energy and NMR calculations. In the structural optimizations, we fixed the cell parameters a = 11.7308 Å, b = 5.8654 Å, and c = 16.5390 Å, which were obtained by the Rietveld analysis of X-ray diffraction data for the mixture of as prepared $Ba_7Nb_4MoO_{20}$ and internal standard silicon.

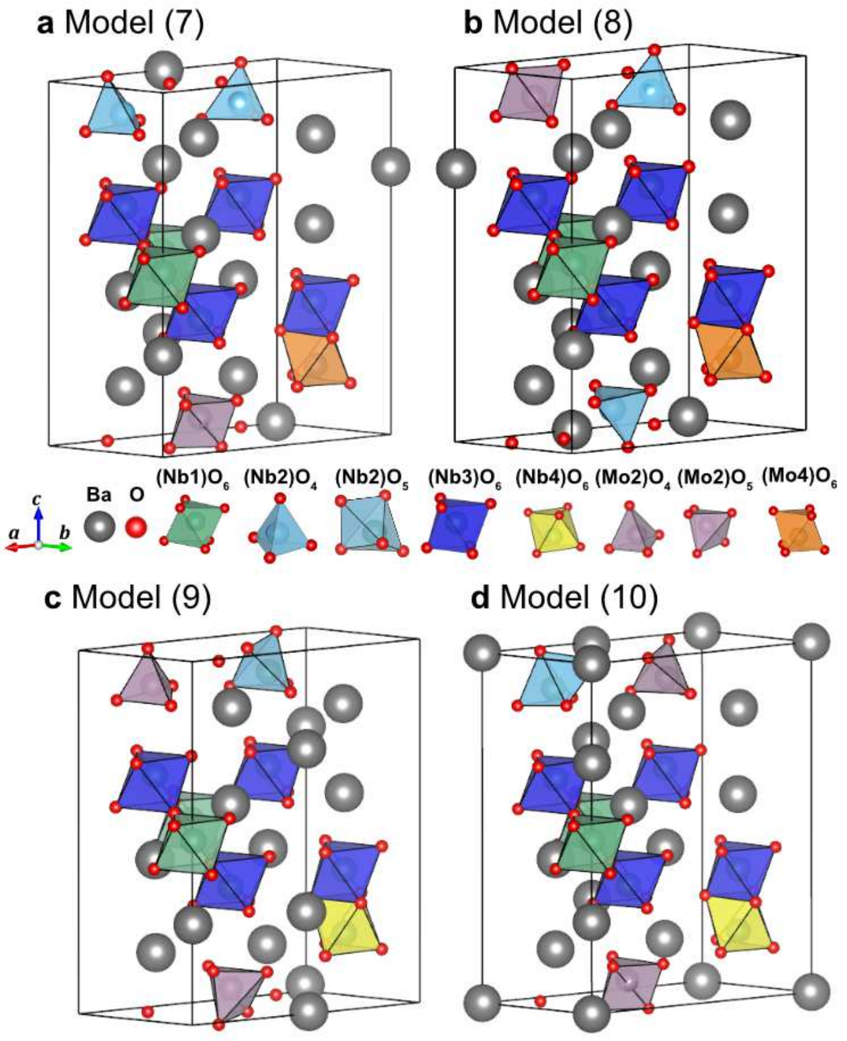

Supplementary Figure 7. Optimized structures for the structural models (7)-(10) with different configurations of Nb and Mo atoms in the 2×1×1 cell of $Ba_7Nb_4MoO_{20}$ used for the calculations of the NMR parameters of the $M2O_5$, where **a,b** Mo and **c,d** Nb are in *M*4 sites. In the structural optimizations, we fixed the cell parameters a = 11.7308 Å, b = 5.8654 Å, and c = 16.5390 Å, which were obtained by the Rietveld analysis of X-ray diffraction data for the mixture of as prepared $Ba_7Nb_4MoO_{20}$ and internal standard silicon.

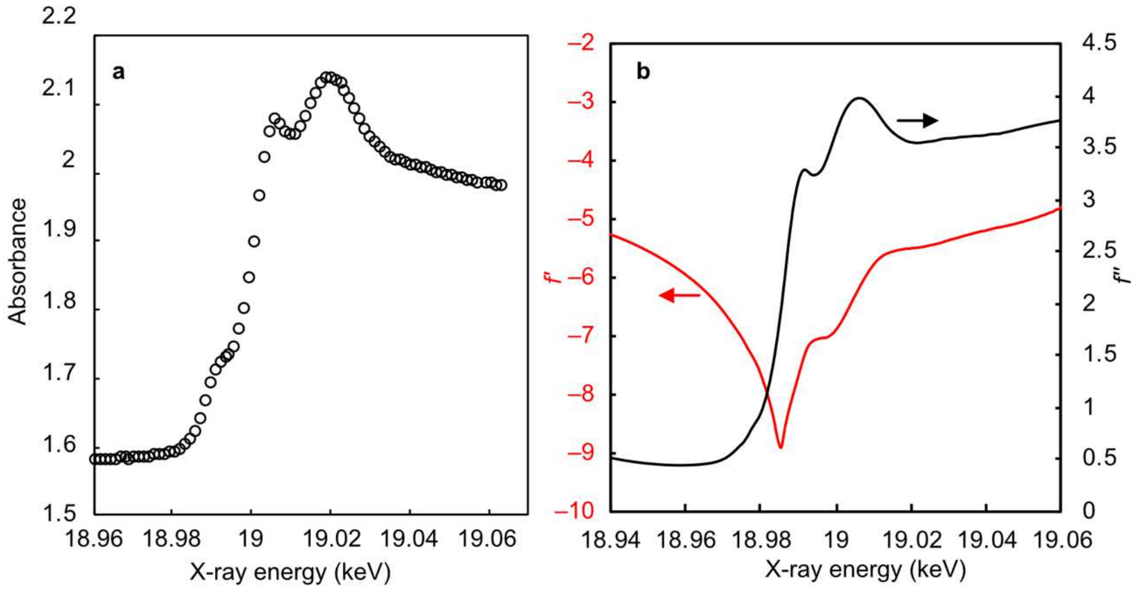

Supplementary Figure 8. a XANES spectrum of $Ba_7Nb_4MoO_{20}\cdot 0.15\ H_2O$ and b resonant (anomalous) scattering factors of Nb atom obtained by Kramer–Kronig transformation from the XANES spectrum, which was carried out with the code DiffKK[12]. We used atomic scattering factors in the form of

$f = f_0 + f' + if''$, where f_0 is the Thomson scattering factor, and f' and f'' are resonant (anomalous) scattering factors. The XANES spectrum at the Nb K-edge was recorded at the beamline BL19B2 in SPring-8.

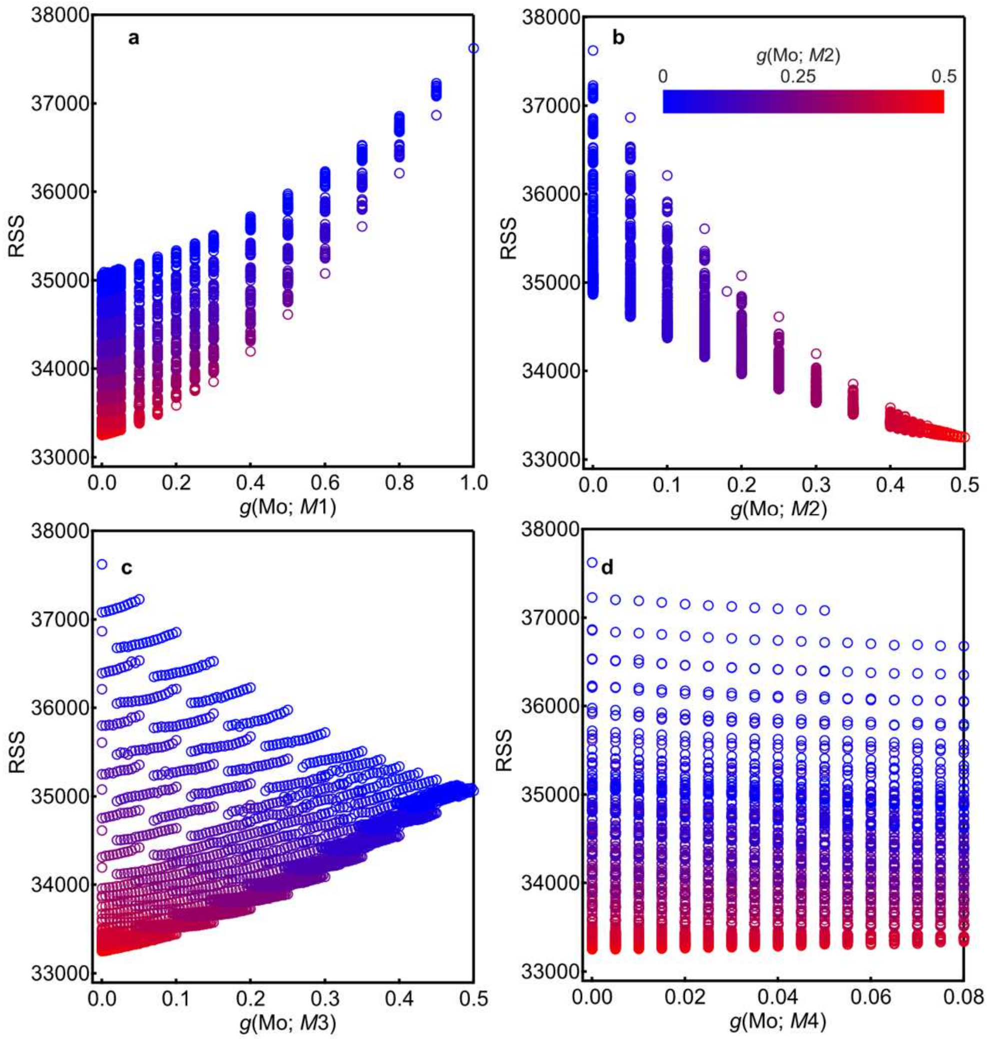

Supplementary Figure 9. Variation of the residual sum of squares (RSS; see the definition of Eq. (3) in the text) with the occupancy factor of Mo atoms at the a *M*1, b *M*2, c *M*3 and d *M*4 sites in the Rietveld analyses for the RXRD data taken with 0.6523630(5) Å X-ray at the BL19B2 beamline of SPring-8. The result indicates that the occupancies of Mo atoms are 0.00 at *M*1, *M*3, and *M*4 sites and 0.50 at *M*2 site: *g*(Mo; *M*1) = *g*(Mo; *M*3) = *g*(Mo; *M*4) = 0.00, and *g*(Mo; *M*2) = 0.50.

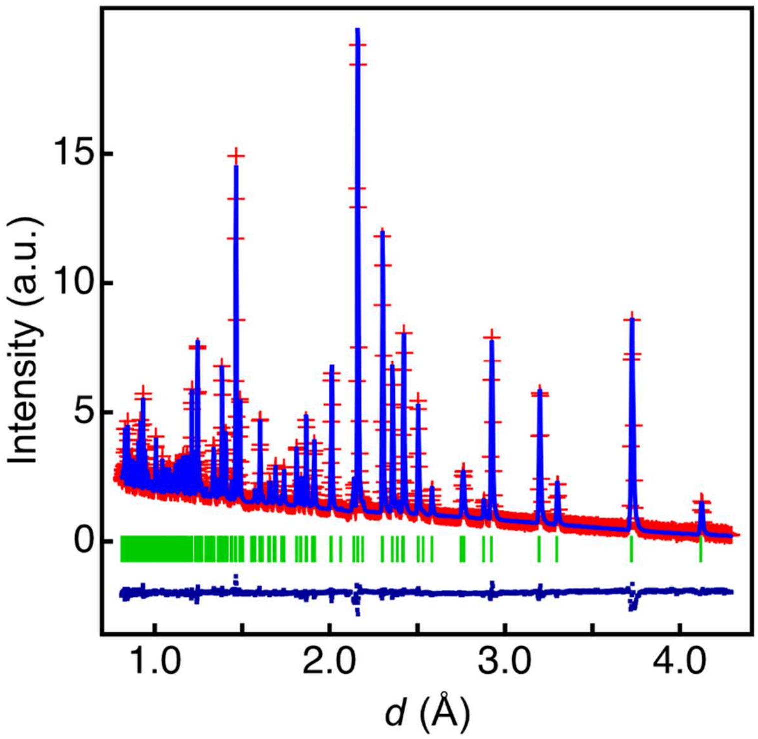

Supplementary Figure 10. Rietveld pattern of ND data of $Ba_7Nb_4MoO_{20}{\cdot}0.15\ H_2O$ at 30 K. The observed and calculated intensities and difference plot are shown by red cross marks, blue solid lines, and blue dots, respectively. Green tick marks denote the calculated Bragg peak positions.

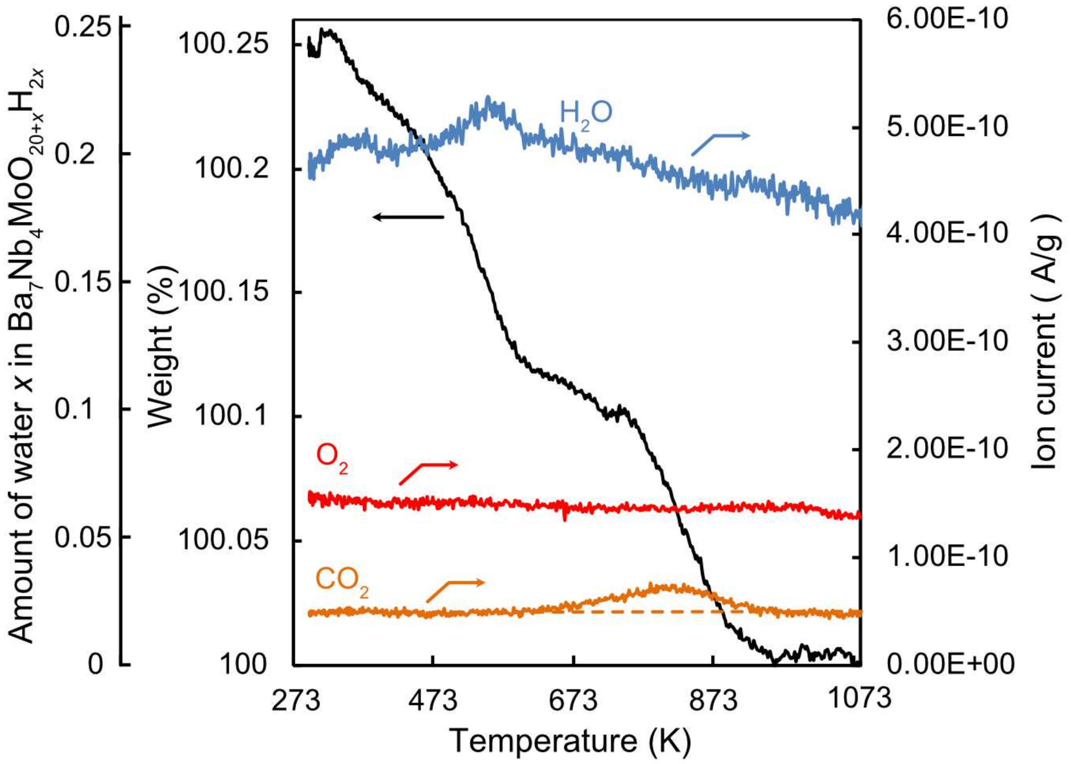

Supplementary Figure 11. TG-MS (thermogravimetric-mass spectroscopic) data of $Ba_7Nb_4MoO_{20}\cdot 0.15\ H_2O$ measured on heating at a rate of 20 K min^{-1} under dry He flow. Blue, red and orange lines denote the intensities of H_2O (m/z = 18), O_2 (m/z = 32) and CO_2 (m/z = 44) molecules. The blue and orange dashed lines are a guide to the eye, which shows possible baselines of MS intensities. Upon heating, the weight of the sample decreases between room temperature and 670 °C. MS measurements confirmed that the released gas between 294 and 673 K is mainly H_2O molecules. The water content x in the bulk crystal $Ba_7Nb_4MoO_{20+x}H_{2x}$ (= $Ba_7Nb_4MoO_{20}\cdot x\ H_2O$) was estimated as follows. Assuming that there is no water ($x = 0$) at 1073 K and that weight loss is due to only dehydration, we obtain the water content $x = 0.25$ at room temperature. Since the CO_2 desorption is observed at around and above 673 K, we can assume that there is no water ($x = 0$) at 673 K. In this case, assuming that the weight loss from 373 to 673 K is ascribed to only water desorption from the bulk, we obtain bulk water content $x = 0.13$. Therefore, the bulk water content is ranges from $x = 0.13$ to 0.25, which is consistent with the calculated water content $x = 0.151(5)$ using the refined occupancy factors at 30 K.

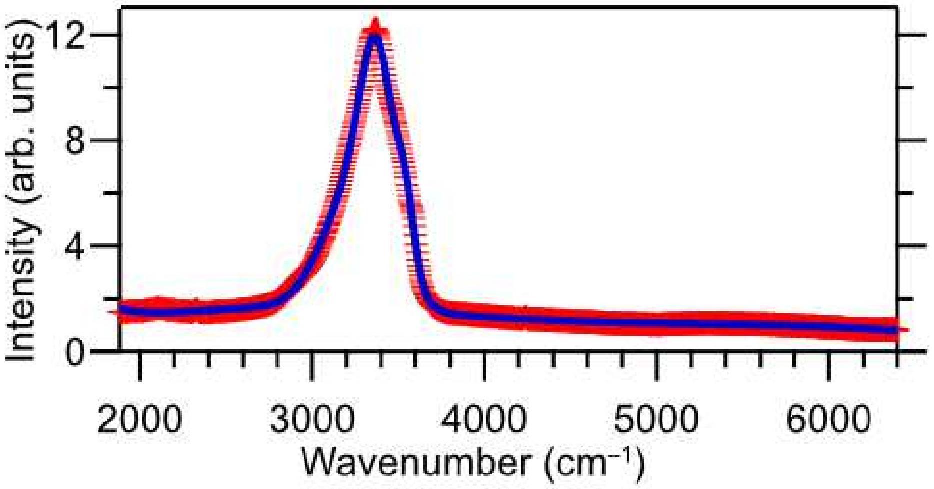

Supplementary Figure 12. Raman spectrum of O–H stretching vibrations of $Ba_7Nb_4MoO_{20}\cdot0.15\ H_2O$. Red cross marks stand for experimental data, and blue solid line was obtained by fitting. The O–H distance was calculated to be 0.99738(8) Å using the empirical equation after Novak.[13]

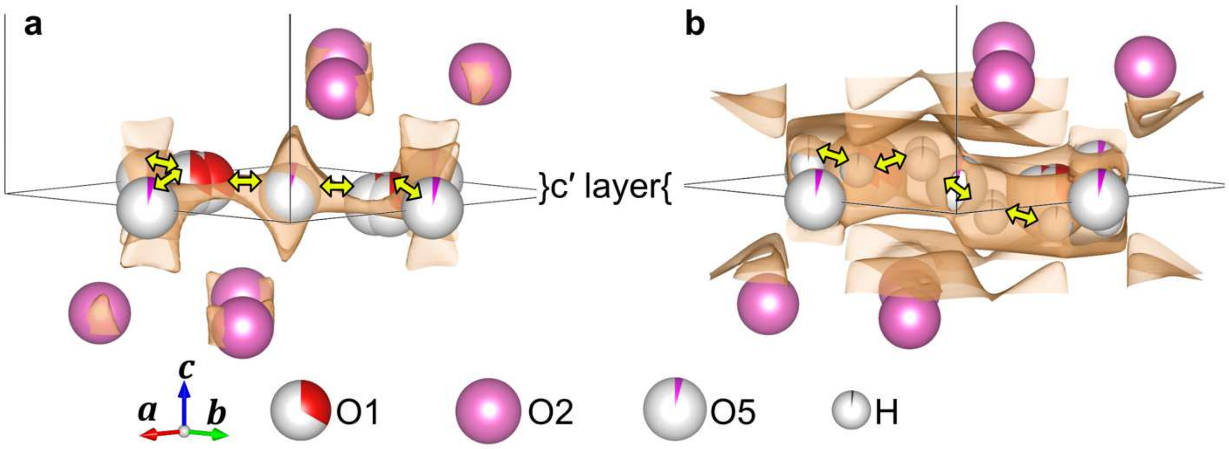

Supplementary Figure 13. Bond-valence-based energy (BVE) landscapes for a an oxide ion and b proton with orange isosurfaces at a 0.22 and b 0.65 eV. The solid lines represent the unit cell. Yellow arrows represent the possible ion migration paths.

The energy barriers for proton migration $E_{b/H}$ were also estimated using the bond-valence method. The $E_{b/H}$ along the *c* axis in Mo-ordered $Ba_7Nb_4MoO_{20}$·0.15 H_2O was slightly higher than that in virtual Mo-disordered $Ba_7Nb_4MoO_{20}$·0.15 H_2O (Supplementary Table 14).

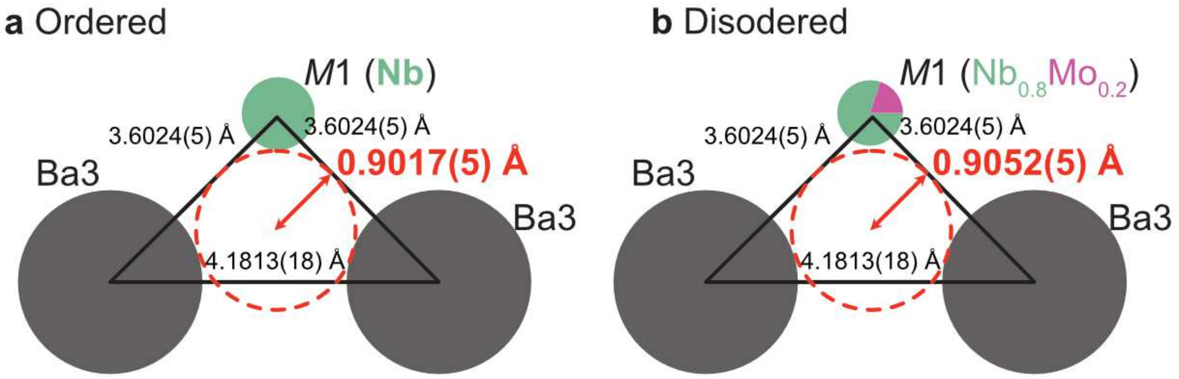

Supplementary Figure 14. The bottlenecks for oxide-ion migration along *c* axis in the cases of a all Mo atoms in the *M*2 sites (ordered $Ba_7Nb_4MoO_{20}$) and b occupationally disordered $Ba_7Nb_4MoO_{20}$. The bottleneck (critical radius) for oxide-ion migration along the *c* axis in the ordered $Ba_7Nb_4MoO_{20}$ 0.9017(5) Å is smaller than that in the disordered $Ba_7Nb_4MoO_{20}$ 0.9052(5) Å. This is the reason for the higher $E_{b/O}$ along the *c* axis in Mo-ordered $Ba_7Nb_4MoO_{20}$·0.15 H_2O compared with Mo-disordered $Ba_7Nb_4MoO_{20}$·0.15 H_2O (Supplementary Table 13).

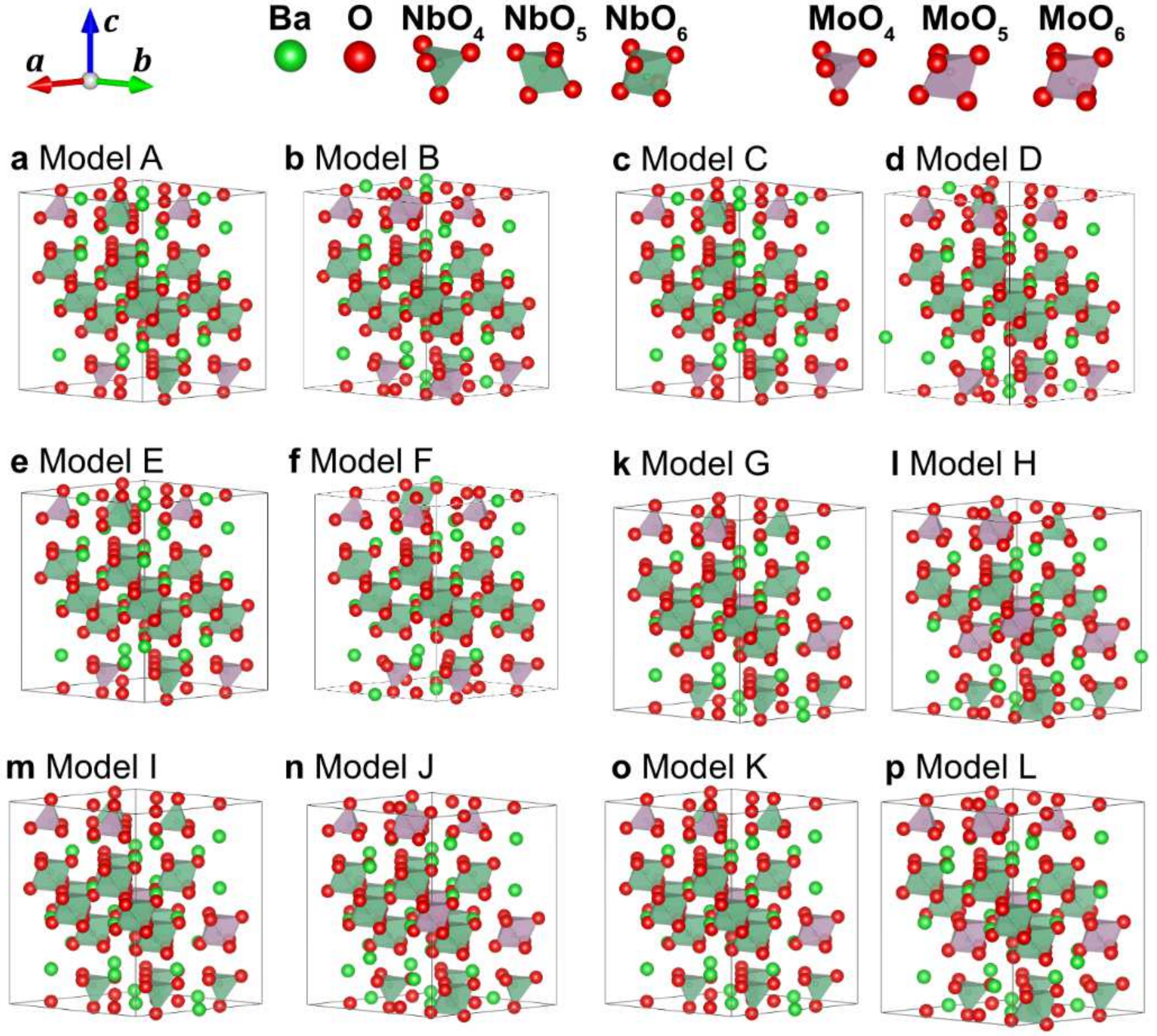

Supplementary Figure 15. Structural models of 2×2×1 supercells $(Ba_7Nb_4MoO_{20})_4$ and $(Ba_7Nb_{3.5}Mo_{1.5}O_{20.25})_4$ to investigate the formation energy of $Ba_7Nb_{3.5}Mo_{1.5}O_{20.25}$. $(Ba_7Nb_{3.5}Mo_{1.5}O_{20.25})_4$ has an interstitial O5 atom.

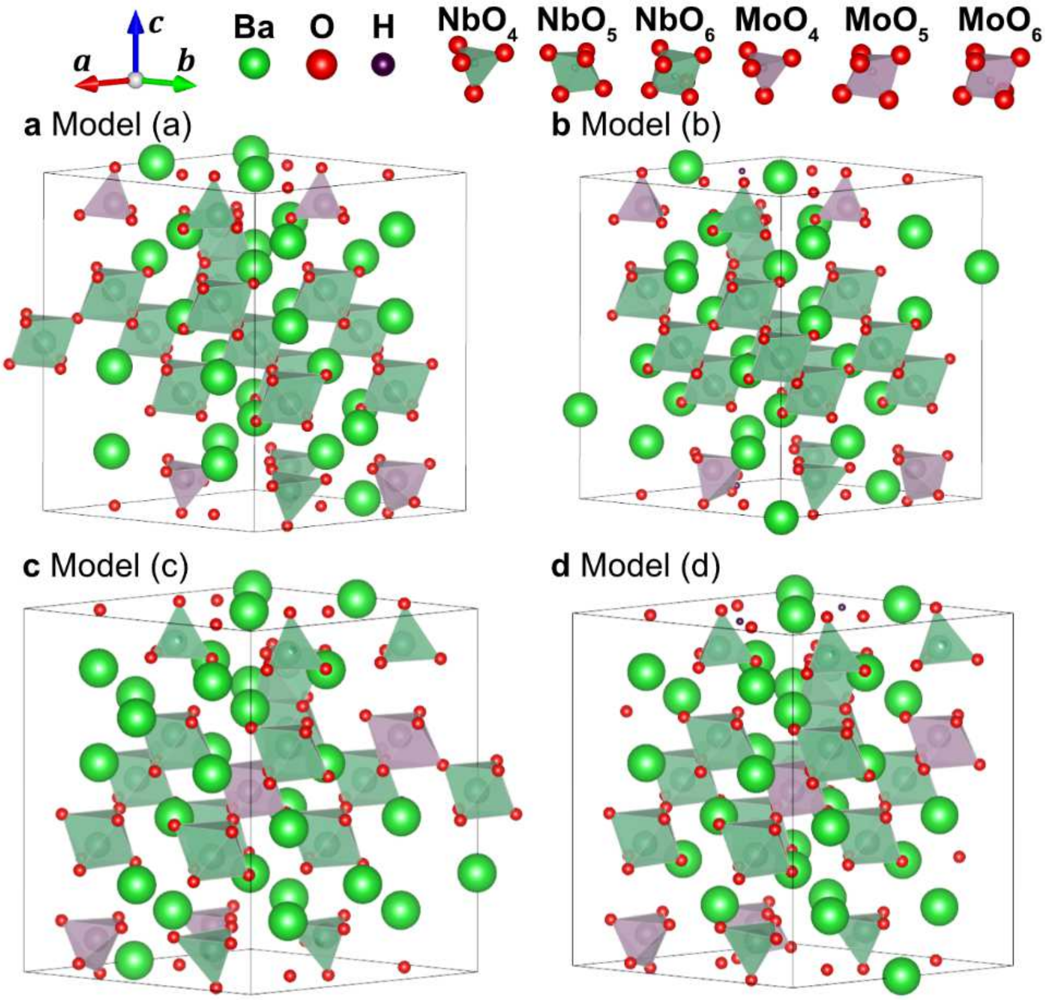

Supplementary Figure 16. Structural models of 2×2×1 supercells $(Ba_7Nb_4MoO_{20})_4$ and $(Ba_7Nb_4MoO_{20.25}H_{0.5})_4$. to investigate the hydration enthalpy. Each model has a Nb atom at the *M*4 site, which stabilizes the hydrated $(Ba_7Nb_4MoO_{20.5}H_{0.5})_4$, which is consistent with the literature.[14]

Supplementary Table 1. Materials containing pair(s) of elements with low Scattering Contrast Score (SCS), which is defined by Eq. (1) in the text.

Material	Element pair	SCS	Properties	References
$Ba_7Nb_4MoO_{20}$	Mo/Nb	0.037	proton and oxide-ion conductivity	14–18
$Ba_3MoNbO_{8.5}$	Mo/Nb	0.037	oxide-ion conductivity	19–21
$Sr_{11}Mo_3NbO_{22.5}$	Mo/Nb	0.037	oxide-ion conductivity	22
$BaGdInO_4$	Ba/In	0.18	oxide-ion conductivity	23
$Mo_3Nb_{14}O_{44}$	Mo/Nb	0.037	lithium-ion conductivity	24
$Ba_7Y_2Ti_3Mn_2O_{20}$	Ti/Mn	0.10	ionic-electronic mixed conductivity	25
$Pr_{0.5}Ba_{0.5}CoO_{3-\delta}$	Pr/Ba	0.077	catalysis	26
Mo-Nb-V-Te-O oxide	Mo/Nb	0.037	catalysis	27,28
$Ag_{1-x}Cd_xSbTe_2$	Ag/Cd, Sb/Te	0.11, 0.030	thermoelectricity	29
$Cd_{0.2}Sn_{0.8}Sb_2Te_4$	Cd/Sn, Sb/Te	0.14, 0.030	thermoelectricity	30
Zr_5Ir_2Os	Ir/Os	0.011	superconductivity	31
$SrSn_{0.95}Sb_{0.05}O_3$	Sn/Sb	0.065	transparency and electronic conductivity	32
$PbTiO_3$-$BiFeO_3$	Pb/Bi	0.055	ferroelectricity	33

Supplementary Table 2. Inorganic crystal structure database (ICSD) code, chemical composition, calculated and experimental ^{95}Mo NMR parameters for molybdenum-containing oxides.

ICSD Code[a]	Coordination polyhedron	Composition	σ_{iso} (ppm)[b]	Calculated δ_{iso} (ppm)[c]	Experimental δ_{iso} (ppm)	Calculated $\|C_Q\|$ (MHz)	Experimental $\|C_Q\|$ (MHz)	References for experimental δ_{iso} and $\|C_Q\|$
**26784**	MoO_4	$PbMoO_4$	982.6	57.7	118.5	2.534	2.03	34
**84455**	MoO_4	$CdMoO_4$	1035.6	92.2	131.3	3.707	3.05	34
**50821**	MoO_4	$BaMoO_4$	835.4	–38.0	–45	1.896	1.68	This work (Supplementary Figure 4.)
**24904**	MoO_4	Rb_2MoO_4	867.0	–17.4	–21	1.3	1.12	34
**11773**	MoO_4	Li_2MoO_4	877.6	–10.6	–26	1.389	1.27	34
**16154**	MoO_4	K_2MoO_4	873.4	–13.3	–17	1.553	1.27	34
**180590**	MoO_6	α–MoO_3	838.0	–36.3	–73	3.001	2.4	This work (Supplementary Figure 4.)
**9278**	MoO_4	Cs_2MoO_4	853.2	–26.4	–22	0.657	0.34	34
**262319**	MoO_6	Ba_2CaMoO_6	1217.2	210.2	168.5	0	0	4
**28025**	MoO_4	$SrMoO_4$	886.8	–4.6	–1	2.453	2.26	34
**62219**	MoO_4	$CaMoO_4$	933.2	25.6	46	3.344	3.05	34

[a] ICSD code of the structure data used as the initial structure before the geometry optimizations.

[b] δ_{iso}[VASP]: Isotropic chemical "shift" obtained by VASP[11]

[c] (Calculated δ_{iso}) = (Calculated isotropic chemical shift δ_{iso}) = 0.65 σ_{iso} – 581 (Supplementary Fig. 5a).

Supplementary Table 3. Inorganic crystal structure database (ICSD) code, chemical composition, calculated and experimental ^{93}Nb NMR parameters of niobium-containing oxides.

ICSD Code[a]	Coordination polyhedron	Composition	σ_{iso} (ppm)[b]	Calculated δ_{iso} (ppm)[c]	Experimental δ_{iso} (ppm)	Calculated $\|C_Q\|$ (MHz)	Experimental $\|C_Q\|$ (MHz)	References for experimental δ_{iso} and $\|C_Q\|$
**20335**	NbO_4	$YNbO_4$	–204	–873	–840	75.89	82.23	7
**81616**	NbO_4	$LaNbO_4$	–234	–906	–853	79.89	86.55	35
**202827**	NbO_6	$SnNb_2O_6$	–336	–1017	–1014	47.24	40.06	7
**23239**	NbO_6	$NaNbO_3$	–355	–1038	–1064	18.9	19.96	36
**91748**	NbO_6	$Nb_2Mg_4O_9$	–193	–861	–921	48.38	45.55	7
**85008**	NbO_6	Nb_2MgO_6	–352	–1035	–1020	57.13	53.81	7
**94493**	NbO_6	$LiNbO_3$	–294	–971	–988	26.36	22.25	7
**79481**	NbO_6	La_3NbO_7	–308	–987	–998	39.38	50.87	7
**9533**	NbO_6	$KNbO_3$	–318	–998	–1019	18.92	22.99	7
**75601**	NbO_6	$Cd_2Nb_2O_7$	–280	–956	–988	28.86	24.79	7
**15208**	NbO_6	$CaNb_2O_6$	–326	–1006	–975	54.52	50.4	7
**74338**	NbO_6	$NbBiO_4$	–288	–965	–969	28.51	20.83	7
**96510**	NbO_6	$CsNb_2BiO_7$	–271	–946	–936	18.27	24.06	7

[a] ICSD code of the crystal structure data used as the initial structure before the geometry optimizations.

[b] δ_{iso}[VASP]: chemical "shift" obtained by VASP[11]

[c] (Calculated δ_{iso}) = (Calculated isotropic chemical shift δ_{iso}) = 1.09 σ_{iso} – 651 (Supplementary Fig. 5d).

Supplementary Table 4. ^{93}Nb NMR parameters and probable assignment of the signals to the sites of $Ba_7Nb_4MoO_{20}$·0.15 H_2O.

Models[a]	Site	Coordination Polyhedron	DFT-calculated δ_{iso} (ppm)[b]	DFT-calculated $\lvert P_Q \rvert$ (MHz)[c]	Experimental δ_{iso} (ppm)[d]	Experimental $\lvert P_Q \rvert$ (MHz)[e]
(1), (2), (3)	*M*1	NbO_6	–800 ~ –810	18 ~ 20	–748	15
(1), (2), (3)	*M*2	NbO_4	–848 ~ –862	6.8 ~ 18	–952	6
(9), (10)	*M*2	NbO_5	–803, –812	75, 43	uncertain	
(1), (2), (3)	*M*3	NbO_6	–867 ~ –897	41 ~ 50	–928	19
(9), (10)	M4	NbO_6	–845, –858	71, 73	uncertain	

[a] Each model is shown in the Supplementary Figures 6 and 7. The models with Mo atoms at the *M*2 site was used.

[b] Calculated isotropic chemical shift: $\delta_{iso} = 1.09 \times \sigma_{iso} - 651$ (Supplementary Fig. 5d).

[c] DFT-calculated P_Q: Quadrupolar product was estimated by the equation: $P_Q = C_Q(1 + \eta^2/3)^{1/2}$ where C_Q and η are DFT-calculated NMR parameters.

[d] Experimental isotropic chemical shift δ_{iso} was estimated using the following equation: $\delta_{iso} = (17\delta_{F1} + 10\delta_{F2})/27$ (Ref. 36–38). Because it is difficult to determine C_Q and η for each peak with unclear shape probably due to the structural disorder,[39] P_Q value was calculated from the positions of the experimental resonances (δ_{F1} and δ_{F2}) in the F1 and F2 dimensions of the 3QMAS spectrum (Fig. 3a).

[e] Experimental $|P_Q|$ was estimated by the following equation:

$$|P_Q| = \left(\frac{10}{27} \cdot \frac{17}{3}\right)^{1/2} \frac{[4I(2I-1)]}{[4I(I+1)-3]^{1/2}} \nu_L \cdot 10^{-3} (\delta_{F1} - \delta_{F2})^{\frac{1}{2}}$$

where ν_L is Larmor frequency and I (= 9/2) is the nuclear spin.[36,40–42]

Supplementary Table 5. Wavelengths and resonant scattering factors used in the Rietveld analyses of the RXRD data.

	Beamline	Wavelength (Å)	f' (Nb)	f''(Nb)
Using the data from XANES spectrum (Supplementary Figure 8)	BL02B2	0.6527887(5)	–7.1808	3.2712
	BL19B2	0.6523630(5)	–6.3453	3.9525

	Beamline	Wavelength (Å)	f' (Ba)	f''(Ba)	f' (Mo)	f''(Mo)	f' (O)	f''(O)
Using theoretical values[43,44]	BL02B2	0.6527887(5)	–0.7251	2.0659	–2.6981	0.6044	0.0052	0.0050
	BL19B2	0.6523630(5)	–0.7263	2.0635	–2.6990	0.6037	0.0052	0.0050

Supplementary Table 6. Refined occupancy factors in a preliminary Rietveld analysis using RXRD data of $Ba_7Nb_4MoO_{20}\cdot 0.15\ H_2O$, which were measured with 0.6527887(5) Å X-ray at the BL02B2 beamline of SPring-8. Linear constraints of Eqs. (2) were used in the analysis. The result is consistent with g(Mo; M1) = g(Mo; M3) = g(Mo; M4) = 0.000.

Site s	g(Nb; s)	g(Mo; s)
M1	1.13(3)	–0.13(3)
M2	0.184(14)	0.736(14)
M3	0.95(2)	0.05(2)
M4	0.30(2)	–0.22(2)

Supplementary Table 7. Refined occupancy factors in a preliminary Rietveld analysis using RXRD data of $Ba_7Nb_4MoO_{20}\cdot 0.15\ H_2O$, which were measured with 0.6527887(5) Å X-ray at the BL02B2 beamline of SPring-8. Linear constraints of Eqs. (2) were used and the g(Nb; M4) and g(Mo; M4) were fixed to the values 0.08 and 0.00, respectively, in the analysis. The result is consistent with g(Mo; M1) = g(Mo; M3) = g(Mo; M4) = 0.000.

Site s	g(Nb; s)	g(Mo; s)
M1	1.152(19)	–0.152(19)
M2	0.340(11)	0.736(14)
M3	1.000(11)	0.000(11)
M4	0.08 (fixed)	0.00 (fixed)

Supplementary Table 8. Refined occupancy factors in a preliminary Rietveld analysis using RXRD data of $Ba_7Nb_4MoO_{20}\cdot 0.15\ H_2O$, which were measured with 0.6527887(5) Å X-ray at the BL02B2 beamline of SPring-8. In the analysis, linear constraints of Eqs. (2) were used and the g(Nb; M1) and g(Mo; M1) were fixed to the values 1.00 and 0.00, respectively, and the g(Nb; M4) and g(Mo; M4) were fixed to the values 0.08 and 0.00, respectively. The result is consistent with g(Mo; M1) = g(Mo; M3) = g(Mo; M4) = 0.000.

Site s	g(Nb; s)	g(Mo; s)
M1	1.00 (fixed)	0.00 (fixed)
M2	0.322(4)	0.598(4)
M3	1.09(4)	–0.09(4)
M4	0.08 (fixed)	0.00 (fixed)

Supplementary Table 9 Refined crystal parameters and reliability factors in the Rietveld analysis of the resonant synchrotron X-ray diffraction data (0.6527887(5) Å) of $Ba_7Nb_4MoO_{19.849}(OH)_{0.302}$ (= $Ba_7Nb_4MoH_{0.302}O_{20.151}$ = $Ba_7Nb_4MoO_{20.151}H_{0.302}$ = $Ba_7Nb_4MoO_{20}$·0.151 H_2O) at 297 K at the BL02B2 line in SPring-8.

Site/Atom label	Atom	Wyckoff position	*g*	*x*	*y*	*z*	U_{iso} (Å^2)	BVS[d]
Ba1	Ba	1*a*	1[e]	0	0	0	0.0170(15)	2.02
Ba2	Ba	2*d*	1[e]	1/3	2/3	0.8264(2)	0.0140(11)	2.18
Ba3	Ba	2*d*	1[e]	1/3	2/3	0.5734(2)	0.0079(9)	2.30
Ba4	Ba	2*c*	1[e]	0	0	0.2800(2)	0.0088(9)	1.93
*M*1	Nb	1*b*	1[e]	0	0	1/2	0.0064(3)	4.59
*M*2	Nb	2*d*	0.420	1/3	2/3	0.0932(4)	0.0064(3)	4.95
	Mo	2*d*	0.500	1/3	2/3	0.0932(4)	0.0064(3)	5.59
*M*3	Nb	2*d*	1[e]	1/3	2/3	0.3478(4)	0.0064(3)	4.65
*M*4	Nb	2*d*	0.08	1/3	2/3	0.1954[b]	0.0064(3)	3.63
O1	O	6*i*	1/3	0.3532[a]	0.7064[a]	–0.01209[a]	0.014(2)	1.96
O2	O	6*i*	1[e]	0.166652[a]	0.33304[a]	0.13082[a]	0.014(2)	1.93
O3	O	6*i*	1[e]	0.16323[a]	0.32646[a]	0.43098[a]	0.014(2)	1.95
O4	O	6*i*	1[e]	0.49502[a]	0.50498[a]	0.29455[a]	0.014(2)	1.98
O5	O	3*e*	0.0504[c]	1/2	0	0	0.014(2)	1.33
H	H	12*j*	0.0252[c]	0.345	0.5	0.9748	0.04106[f]	0.84

Crystal system: trigonal. Space group: $P\bar{3}m1$ (No.164, setting 1). Lattice parameters: $a = b$ = 5.8604(4) Å, c = 16.5250(9) Å

Number of formula per unit cell: Z = 1. $g(X; s)$: Occupancy factor of X atom at the s site. g(Ba; Ba1) = g(Ba; Ba2) = g(Ba; Ba3) = g(Ba; Ba4) = g(Nb; M1) = g(Nb; M3) = g(O; O2) = g(O; O3) = g(O; O4) = 1; g(Nb; M2) = 0.42, g(Mo; M2) = 0.5, g(Nb; M4) = 0.08; g(O; O1)=1/3, g(O; O5) = 0.0504.

$U_{iso}(Xn)$ Isotropic atomic displacement parameter of X atom at the Xn site. Linear constraints in the Rietveld analysis: U_{iso}(Nb1) = U_{iso}(Nb2) = U_{iso}(Mo2) = U_{iso}(Nb3) = U_{iso}(Nb4), U_{iso}(O1) = U_{iso}(O2) = U_{iso}(O3) = U_{iso}(O4) = U_{iso}(O5).

Reliability factors: R_{wp} = 6.732%, R_p = 4.503%, R_B = 3.874%, R_F = 2.235%. Goodness of Fit GoF = 65.5773

[a] Atomic coordinates of Oi (i = 1–5) atoms were fixed to those from ND analysis at 300 K.

[b] z coordinate of Nb4 atom was fixed to those from a preliminary analysis.

[c] Occupational parameters of O5 and H atom were fixed to those from ND analysis at 300 K (Table 2).

[d] BVS: Bond valence sums. Here the bond valence parameters after Adams and Rao[45] were used for the calculations of BVSs. BVS values are consistent with the formal charge of Ba^{2+}, Nb^{5+}, Mo^{6+}, O^{2-} and H^+.

[e] The occupancy factors of Ba1–4, *M*1, *M*3, O2–O4 were fixed to unity, because the refined values agreed with unity within three times of estimated standard deviations (see Supplementary Note 1 for details).

[f] Atomic displacement parameter of H atom was fixed to those from preliminary analyses.

Supplementary Table 10 Refined crystal parameters and reliability factors in Rietveld analysis of the neutron diffraction data of $Ba_7Nb_4MoO_{19.849(5)}(OH)_{0.302(8)}$ (= $Ba_7Nb_4MoH_{0.302(8)}O_{20.151(5)}$ = $Ba_7Nb_4MoO_{20.151(5)}H_{0.302(8)}$ = $Ba_7Nb_4MoO_{20}\cdot0.151(5)\ H_2O$) at 30 K.

Site / Atom label	Atom	Wyckoff Position	*g*	*x*	*y*	*z*	U_{iso} (Å^2)	BVS^c
Ba1	Ba	1*a*	1^b	0	0	0	0.00092^a	2.03
Ba2	Ba	2*d*	1^b	1/3	2/3	0.82204(5)	0.00092^a	2.24
Ba3	Ba	2*d*	1^b	1/3	2/3	0.57321(7)	0.00092^a	2.31
Ba4	Ba	2*c*	1^b	0	0	0.27876(6)	0.00092^a	1.94
*M*1	Nb	1*b*	1^b	0	0	1/2	0.002^a	4.63
*M*2	Nb	2*d*	0.42	1/3	2/3	0.09437(5)	0.002^a	4.80
	Mo	2*d*	0.5	1/3	2/3	0.09437(5)	0.002^a	5.37
*M*3	Nb	2*d*	1^b	1/3	2/3	0.34875(4)	0.002^a	4.67
*M*4	Nb	2*d*	0.08	1/3	2/3	0.1954	0.002^a	3.6
O1	O	6*i*	1/3	0.3183(5)	0.6366(10)	–0.01323(5)	0.0169(7)	1.83
O2	O	6*i*	1^b	0.16527(11)	0.3305(2)	0.13137(3)	0.00764(12)	1.91
O3	O	6*i*	1^b	0.16307(12)	0.3262(2)	0.43120(3)	0.00506(14)	1.96
O4	O	6*i*	1^b	0.49448(8)	0.50552(8)	0.29476(2)	0.00484(14)	2.02
O5	O	3*e*	0.0504(15)	1/2	0	0	0.0169(7)	1.29
H	H	12*j*	0.0252(7)	0.346(3)	0.500(3)	0.9748(11)	0.005^a	0.84

Crystal system: trigonal. Space group: $P\bar{3}m1$ (No.164, setting 1). Lattice parameters: $a = b = 5.856523(4)$ Å, $c = 16.51565(3)$ Å

Number of formula per unit cell: $Z = 1$. $g(X; s)$: Occupancy factor of X atom at the s site. g(Ba; Ba1) = g(Ba; Ba2) = g(Ba; Ba3) = g(Ba; Ba4) = g(Nb; M1) = g(Nb; M3) = g(O; O2) = g(O; O3) = g(O; O4) = 1; g(Nb; M2) = 0.42, g(Mo; M2) = 0.5, g(Nb; M4) = 0.08; g(O; O1)=1/3, g(O; O5) = 2g(H; H1).

$U_{iso}(Xn)$: Isotropic atomic displacement parameter (ADP) of X atom at the Xn site.

Reliability factors: $R_{wp} = 2.844\%$, $R_p = 2.713\%$, $R_B = 4.996\%$, $R_F = 4.201\%$. Goodness of Fit GoF = 22.790.

[a] ADPs of Ba*i*, *Mi* (*i* = 1–4) and H atom were fixed to those from preliminary analyses.

[b] The occupancy factors of Ba1–4, *M*1, *M*3, O2–O4 were fixed to unity, because the refined values agreed with unity within three times of estimated standard deviations (see Supplementary Note 1 for details).

[c] BVS: Bond valence sums. Here the bond valence parameters after Adams and Rao[45] were used for the calculations of BVSs. BVS values are consistent with the formal charge of Ba^{2+}, Nb^{5+}, Mo^{6+}, O^{2-} and H^+.

Supplementary Table 11 Total energies of $(Ba_7Nb_4MoO_{20})_2$ (2×1×1 cell) with different arrangements of the Nb and Mo atoms (Supplementary Figure 6), which were obtained by DFT calculations. The total energies for the models where Mo atoms are located only at the *M*2 site are a little lower than those for other models, which suggests that the Mo chemical order at the *M*2 site is energetically favorable.

Model	Mo arrangement	Relative energy (meV per atom)
(1)	Mo atoms in only *M*2 site	+1.68
(2)	Mo atoms in only *M*2 site	+1.45
(3)	Mo atoms in only *M*2 site	0.00
(4)	Mo atoms in only *M*1 site	+29.96
(5)	Mo atoms in *M*1 and *M*2 sites	+12.50
(6)	Mo atoms in only *M*3 site	+17.94

Supplementary Table 12. Polyhedral volumes and bond valence sums (BVSs) of Mo^{6+} atom at each site in the crystallographic parameters refined using neutron diffraction data at 300 K.

Site	Polyhedron	Polyhedral volume (Å^3)	BVS of Mo^{6+}
*M*1	MoO_6	10.8855	4.63
*M*2	MoO_4	3.6908	5.53
*M*3	MoO_6	11.1086	4.76
*M*4	MoO_6	13.0783	3.51

Supplementary Table 13. Bond-valence-based energy barriers for oxide-ion migration $E_{b/O}$ in the Mo-ordered and Mo-disordered $Ba_7Nb_4MoO_{20}$. $E_{b/O}$ in the *ab* plane is much lower than that along *c* axis, which indicates two-dimensional oxide-ion diffusion (Supplementary Fig. 13). $E_{b/O}$ (eV) along *c* axis for the Mo-ordered $Ba_7Nb_4MoO_{20}\cdot0.15\ H_2O$ 1.93 eV is higher than that for the Mo-disordered model 1.60 eV.

Mo order/disorder	$E_{b/O}$ (eV) in *ab* plane	$E_{b/O}$ (eV) along *c* axis direction
Mo-ordered $Ba_7Nb_4MoO_{20}$*	0.22	1.93
Virtual Mo-disordered $Ba_7Nb_4MoO_{20}$**	0.18	1.60

* $E_{b/O}$ was calculated by the bond-valence method for the crystal structure refined using the neutron-diffraction data at 300 K (Table 2) where the occupancy factors of Mo and Nb atoms in Table 2 were used for the Mo-ordered $Ba_7Nb_4MoO_{20}\cdot0.15\ H_2O$.

** $E_{b/O}$ was calculated by the bond-valence method for the crystal structure refined using the neutron-diffraction data at 300 K (Table 2) where g(Nb; M1) = g(Nb; M3) = 0.8, g(Mo; M1) = g(Mo; M3) = 0.2, g(Nb; M2) = 0.736, g(Mo; M2) = 0.184, g(Nb; M4) = 0.064, g(Mo; M4) = 0.016 for the virtual Mo-disordered $Ba_7Nb_4MoO_{20}\cdot0.15\ H_2O$.

Supplementary Table 14. Energy barriers for proton migration $E_{b/H}$ in $Ba_7Nb_4MoO_{20}$. The $E_{b/H}$ along the *c* axis in Mo-ordered $Ba_7Nb_4MoO_{20}\cdot0.15\ H_2O$ (0.87 eV) is slightly higher than that in virtual Mo-disordered $Ba_7Nb_4MoO_{20}\cdot0.15\ H_2O$ (0.83 eV).

Mo order/disorder	$E_{b/H}$ (eV) in *ab* plane	$E_{b/H}$ (eV) along *c* axis direction
Mo-ordered $Ba_7Nb_4MoO_{20}$*	0.60	0.87
Virtual Mo-disordered $Ba_7Nb_4MoO_{20}$**	0.61	0.83

* $E_{b/H}$ was calculated by the bond-valence method for the crystal structure refined using the neutron-diffraction data at 300 K (Table 2) where the occupancy factors of Mo and Nb atoms in Table 2 were used for the Mo-ordered $Ba_7Nb_4MoO_{20}\cdot0.15\ H_2O$.

** $E_{b/H}$ was calculated by the bond-valence method for the crystal structure refined using the neutron-diffraction data at 300 K (Table 2) where g(Nb; M1) = g(Nb; M3) = 0.8, g(Mo; M1) = g(Mo; M3) = 0.2, g(Nb; M2) = 0.736, g(Mo; M2) = 0.184, g(Nb; M4) = 0.064, g(Mo; M4) = 0.016 for the virtual Mo-disordered $Ba_7Nb_4MoO_{20}\cdot0.15\ H_2O$.

Supplementary Table 15. Formation energies of $Ba_7Nb_{3.5}Mo_{1.5}O_{20.25}$ with interstitial oxygen O5 atoms (ΔH_f), which were calculated by the DFT method. ΔH_f values for the Mo ordered models are lower than those for the Mo disordered ones.

Reactions[a]	Mo configuration of the mother composition[b]	Mo configuration of $Ba_7Nb_{3.5}Mo_{1.5}O_{20.25}$	ΔH_f (kJ per mol of $Ba_7Nb_{3.5}Mo_{1.5}O_{20.25}$)[b]	Note
Model A + MoO_3 → Model B + $NbO_{2.5}$	Four Mo2	Six Mo2	−69.6	Mo ordered model
Model C + MoO_3 → Model D + $NbO_{2.5}$	Four Mo2	Six Mo2	−71.3	Mo ordered model
Model E + MoO_3 → Model F + $NbO_{2.5}$	Four Mo2	Six Mo2	−61.1	Mo ordered model
Model G + MoO_3 → Model H + $NbO_{2.5}$	One Mo1, two Mo2 and one Mo3	Two Mo1, two Mo2 and two Mo3	+14.2	Mo disordered model
Model I + MoO_3 → Model J + $NbO_{2.5}$	One Mo1, two Mo2 and one Mo3	Two Mo1, three Mo2, and one Mo3	−14.8	Mo disordered model
Model K + MoO_3 → Model L + $NbO_{2.5}$	One Mo1, two Mo2 and one Mo3	One Mo1, three Mo2 and two Mo3	−35.2	Mo disordered model

[a] Each model is shown in the Supplementary Fig. 15.

[b] Mo*i* (*i* = 1, 2, 3): a Mo atom located at a *M*2 site.

Supplementary Note 1 Preliminary Analyses of SXRD and ND data of $Ba_7Nb_4MoO_{20}$.

The occupancy factors of atom X at site s $g(X; s)$ of $Ba_7Nb_4MoO_{20}\cdot 0.15\ H_2O$ were refined in preliminary Rietveld analyses using neutron diffraction (ND) data at 300 K and conventional synchrotron X-ray diffraction (SXRD) data at 297 K with 0.6994806(5) Å X-ray far from the Nb K edge as follows.

In the preliminary analyses, the Nb and Mo atoms were assumed to be completely disordered. In the preliminary Rietveld analyses, the following linear constraint was used to maintain the chemical composition:

$g(Nb_{0.8}Mo_{0.2}; M1) + 2g(Nb_{0.8}Mo_{0.2}; M2) + 2g(Nb_{0.8}Mo_{0.2}; M3) + 2g(Nb_{0.8}Mo_{0.2}; M4) = 5$ Eq. (S1).

The occupancy factors of Ba atoms were refined to be $g(Ba; Ba1) = 0.997(7)$, $g(Ba; Ba2) = 0.991(6)$, $g(Ba; Ba3) = 0.995(5)$, $g(Ba; Ba4) = 1.000(5)$ in a preliminary Rietveld analysis for the conventional SXRD data. The occupancy factors of the oxygen atoms were refined using the ND data as $g(O; O2) = 1.009(10)$, $g(O; O3) = 0.942(12)$ and $g(O; O4) = 0.985(11)$. The occupancy factors of the Ba1, Ba2, Ba3, Ba4, Nb1, Nb3, O2, O3, and O4 atoms agreed with 1 within 5*esd* where *esd* is the estimated standard deviation. Therefore, the occupancy factors of Ba1, Ba2, Ba3, Ba4, Nb1, Nb3, O2, O3, and O4 atoms were fixed to unity in the following and final refinements:

$g(Ba; Baj) = g(O; Ok) = 1$ (j =1, 2, 3, and 4; k =2, 3, and 4) Eq. (S2).

In another preliminary analysis, the occupancy factors $g(Nb_{0.8}Mo_{0.2}; Mi)$ (i = 1 and 3) were determined to be $g(Nb_{0.8}Mo_{0.2}; M1) = 1.015(7)$ and $g(Nb_{0.8}Mo_{0.2}; M3) = 0.998(8)$. Thus, we fixed these values to unity in the following analysis

$g(Nb_{0.8}Mo_{0.2}; M1) = g(Nb_{0.8}Mo_{0.2}; M3) = 1$ Eq. (S3),

which indicates that the sum of the occupancy factors of Nb and Mo atoms at $M1$ and $M3$ sites are also unity,

$g(Nb; M1) + g(Mo; M1) = 1$, $g(Nb; M3) + g(Mo; M3) = 1$ Eq. (S4).

It was difficult to refine the occupancy factor of $Nb_{0.8}Mo_{0.2}$ at the $M4$ site $g(Nb_{0.8}Mo_{0.2}; M4)$, because of the small occupancy and strong correlation. Thus, we carefully examined the residual sum of squares (RSS) in the Rietveld analyses of SXRD data for various fixed $g(Nb_{0.8}Mo_{0.2}; M4)$ values:

$$\mathrm{RSS} = \sum_i \frac{1}{y_i^{\mathrm{obs}}}\left[y_i^{\mathrm{obs}} - y_i^{\mathrm{cal}}\right]^2.$$

Here y_i^{obs} and y_i^{cal} are the observed and calculated intensities, respectively, at the ith step. The minimum RSS value was obtained for $g(Nb_{0.8}Mo_{0.2}; M4) = 0.08$ among $g(Nb_{0.8}Mo_{0.2}; M4) = 0, 0.005, ..., 0.30$ (Supplementary Fig. 2), which indicates that the sum of the Nb and Mo occupancies at the $M4$ site is 0.08: $g(Nb_{0.8}Mo_{0.2}; M4) = g(Nb; M4)+g(Mo; M4) = 0.08$ Eq. (S5).

Using Eqs. (S1), (S3), (S4), (S5), we obtain

$g(Nb_{0.8}Mo_{0.2}; M2) = g(Nb; M2) + g(Mo; M2) =0.92$ Eq. (S6).

From Eqs. (S2), (S4), (S5) and (S6), we obtain Eq. (2) in the main text.

Supplementary Data. Detailed Scattering Contrast Score (SCS) values.

	H	D	He	Li	Be	B	C	N	O
H	0.000	3.550	14.945	0.826	3.446	6.457	4.287	3.080	5.401
D	3.550	0.000	0.677	2.296	0.677	0.781	0.716	0.918	0.847
He	14.945	0.677	0.000	3.994	0.743	0.667	0.842	1.039	0.881
Li	0.826	2.296	3.994	0.000	1.788	2.368	2.134	1.909	2.428
Be	3.446	0.677	0.743	1.788	0.000	0.301	0.279	0.364	0.480
B	6.457	0.781	0.667	2.368	0.301	0.000	0.204	0.444	0.276
C	4.287	0.716	0.842	2.134	0.279	0.204	0.000	0.246	0.211
N	3.080	0.918	1.039	1.909	0.364	0.444	0.246	0.000	0.301
O	5.401	0.847	0.881	2.428	0.480	0.276	0.211	0.301	0.000
F	5.705	0.883	0.905	2.512	0.543	0.318	0.281	0.372	0.072
Ne	10.861	1.006	0.834	2.964	0.689	0.408	0.436	0.521	0.230
Na	68.439	1.129	0.746	3.768	0.831	0.562	0.588	0.663	0.388
Mg	6.417	0.954	0.959	2.694	0.683	0.419	0.439	0.534	0.238
Al	25.643	1.176	0.762	4.078	0.916	0.656	0.685	0.761	0.493
Si	20.101	1.100	0.870	3.337	0.861	0.595	0.631	0.719	0.439
P	7.251	1.006	0.988	2.843	0.785	0.516	0.557	0.656	0.366
S	8.266	1.284	0.845	5.697	1.065	0.825	0.855	0.925	0.675
Cl	3.170	1.068	1.282	2.195	0.722	0.833	0.659	0.428	0.605
Ar	3.981	1.450	1.061	423.937	1.243	1.036	1.054	1.101	0.890
K	108.277	1.190	0.869	3.874	1.012	0.765	0.808	0.898	0.633
Ca	9.686	1.078	0.999	3.096	0.914	0.660	0.710	0.813	0.534
Sc	2.784	1.205	1.407	2.116	0.904	1.013	0.854	0.635	0.807
Ti	0.955	4.040	38.463	1.048	3.272	5.322	3.715	2.678	4.374
V	1.731	2.038	2.106	1.434	1.807	1.798	1.708	1.619	1.625
Cr	71.824	1.215	0.901	3.968	1.078	0.842	0.893	0.989	0.730
Mn	0.924	4.460	15.724	1.111	3.562	6.418	4.171	2.888	5.114
Fe	3.235	1.098	1.344	2.296	0.830	0.959	0.799	0.581	0.769
Co	5.916	1.385	0.996	8.241	1.257	1.048	1.091	1.168	0.942
Ni	3.071	1.145	1.386	2.259	0.889	1.017	0.863	0.648	0.835
Cu	3.813	1.006	1.277	2.466	0.762	0.892	0.732	0.707	0.709
Zn	5.788	1.016	1.146	2.823	0.921	0.749	0.745	0.866	0.590
Ga	4.045	0.982	1.261	2.529	0.805	0.880	0.722	0.756	0.703
Ge	3.621	1.041	1.313	2.433	0.803	0.944	0.788	0.708	0.770
As	4.573	0.948	1.223	2.645	0.868	0.845	0.697	0.824	0.673
Se	3.710	1.032	1.308	2.464	0.801	0.945	0.791	0.739	0.776
Br	4.391	0.954	1.243	2.618	0.863	0.874	0.718	0.825	0.707
Kr	3.783	1.025	1.306	2.489	0.801	0.948	0.795	0.765	0.784
Rb	4.179	0.978	1.267	2.582	0.852	0.906	0.753	0.820	0.744
Sr	4.228	0.974	1.266	2.596	0.862	0.907	0.755	0.832	0.747
Y	3.814	1.025	1.310	2.507	0.817	0.960	0.810	0.790	0.803
Zr	4.137	0.987	1.279	2.583	0.860	0.927	0.776	0.835	0.771
Nb	4.208	0.980	1.275	2.601	0.872	0.925	0.774	0.849	0.771
Mo	4.466	0.957	1.255	2.656	0.900	0.905	0.755	0.879	0.753
Tc	4.398	0.964	1.263	2.645	0.898	0.916	0.767	0.878	0.765
Ru	4.228	0.982	1.279	2.613	0.885	0.936	0.788	0.868	0.788
Rh	5.449	1.020	1.202	2.830	0.976	0.852	0.826	0.959	0.705
Pd	5.402	1.018	1.206	2.825	0.977	0.858	0.828	0.962	0.713
Ag	5.384	1.018	1.208	2.825	0.979	0.863	0.831	0.966	0.719
Cd	8.571	1.115	1.118	3.162	1.077	0.854	0.932	1.061	0.802
In	24.899	1.203	1.031	3.640	1.163	0.947	1.023	1.144	0.895
Sn	4.969	0.995	1.236	2.765	0.964	0.898	0.818	0.956	0.759
Sb	6.046	1.051	1.186	2.924	1.021	0.846	0.878	1.012	0.749
Te	5.591	1.032	1.206	2.865	1.004	0.870	0.861	0.998	0.734

Supplementary Data- continued

	H	D	He	Li	Be	B	C	N	O
I	6.816	1.079	1.164	3.017	1.052	0.829	0.911	1.045	0.785
Xe	8.296	1.115	1.132	3.153	1.088	0.868	0.949	1.081	0.824
Cs	6.413	1.068	1.179	2.976	1.044	0.845	0.905	1.041	0.780
Ba	7.583	1.101	1.148	3.097	1.044	0.858	0.941	1.075	0.817
La	3.627	1.071	1.365	2.499	0.897	1.056	0.917	0.845	0.927
Ce	8.758	1.125	1.128	3.194	1.105	0.887	0.970	1.103	0.848
Pr	10.858	1.153	1.103	3.321	1.133	0.917	0.999	1.131	0.879
Nd	3.860	1.038	1.340	2.561	0.881	1.030	0.891	0.889	0.905
Pm	2.812	1.275	1.525	2.261	1.113	1.256	1.130	0.942	1.137
Sm	2.513	1.754	1.543	3.362	1.693	1.588	1.609	1.640	1.529
Eu	4.117	1.008	1.316	2.623	0.919	1.006	0.867	0.929	0.883
Gd	4.678	0.982	1.271	2.737	0.973	0.957	0.840	0.983	0.834
Tb	4.024	1.020	1.328	2.605	0.911	1.021	0.883	0.924	0.900
Dy	2.538	1.404	1.618	2.166	1.255	1.382	1.269	1.095	1.273
Ho	3.721	1.062	1.364	2.536	0.901	1.065	0.929	0.889	0.946
Er	3.817	1.048	1.353	2.561	0.889	1.053	0.917	0.905	0.936
Tm	4.216	1.000	1.312	2.652	0.939	1.008	0.871	0.955	0.891
Yb	2.832	1.273	1.529	2.279	1.121	1.269	1.145	0.959	1.158
Lu	4.127	1.011	1.322	2.635	0.932	1.021	0.885	0.950	0.906
Hf	3.861	1.044	1.351	2.575	0.901	1.055	0.920	0.920	0.940
Ta	4.331	0.991	1.306	2.680	0.956	1.004	0.868	0.976	0.890
W	8.644	1.130	1.144	3.206	1.129	0.917	1.005	1.144	0.893
Re	3.343	1.133	1.425	2.444	0.982	1.144	1.013	0.838	1.034
Os	3.048	1.206	1.482	2.356	1.057	1.214	1.087	0.898	1.106
Ir	3.064	1.202	1.479	2.362	1.054	1.211	1.085	0.895	1.104
Pt	3.251	1.155	1.443	2.419	1.007	1.168	1.039	0.848	1.060
Au	3.897	1.042	1.352	2.590	0.914	1.061	0.928	0.939	0.952
Hg	2.811	1.286	1.542	2.280	1.144	1.293	1.173	0.990	1.191
Tl	3.460	1.112	1.410	2.481	0.965	1.131	1.000	0.873	1.024
Pb	3.296	1.146	1.438	2.436	1.001	1.164	1.036	0.845	1.059
Bi	3.536	1.099	1.400	2.503	0.954	1.120	0.989	0.891	1.015

Supplementary Data. continued

	F	Ne	Na	Mg	Al	Si	P	S	Cl
H	5.705	10.861	68.439	6.417	25.643	20.101	7.251	8.266	3.170
D	0.883	1.006	1.129	0.954	1.176	1.100	1.006	1.284	1.068
He	0.905	0.834	0.746	0.959	0.762	0.870	0.988	0.845	1.282
Li	2.512	2.964	3.768	2.694	4.078	3.337	2.843	5.697	2.195
Be	0.543	0.689	0.831	0.683	0.916	0.861	0.785	1.065	0.722
B	0.318	0.408	0.562	0.419	0.656	0.595	0.516	0.825	0.833
C	0.281	0.436	0.588	0.439	0.685	0.631	0.557	0.855	0.659
N	0.372	0.521	0.663	0.534	0.761	0.719	0.656	0.925	0.428
O	0.072	0.230	0.388	0.238	0.493	0.439	0.366	0.675	0.605
F	0.000	0.159	0.318	0.168	0.424	0.371	0.299	0.610	0.565
Ne	0.159	0.000	0.162	0.172	0.270	0.215	0.258	0.463	0.614
Na	0.318	0.162	0.000	0.237	0.109	0.187	0.325	0.306	0.665
Mg	0.168	0.172	0.237	0.000	0.258	0.206	0.134	0.450	0.453
Al	0.424	0.270	0.109	0.258	0.000	0.129	0.267	0.199	0.604
Si	0.371	0.215	0.187	0.206	0.129	0.000	0.140	0.253	0.492
P	0.299	0.258	0.325	0.134	0.267	0.140	0.000	0.318	0.365
S	0.610	0.463	0.306	0.450	0.199	0.253	0.318	0.000	0.572
Cl	0.565	0.614	0.665	0.453	0.604	0.492	0.365	0.572	0.000
Ar	0.829	0.696	0.552	0.676	0.449	0.495	0.549	0.256	0.696
K	0.570	0.419	0.272	0.414	0.219	0.213	0.284	0.212	0.501
Ca	0.471	0.348	0.419	0.317	0.366	0.239	0.187	0.357	0.423
Sc	0.770	0.813	0.856	0.664	0.797	0.695	0.578	0.759	0.229
Ti	4.522	7.471	37.146	4.844	626.348	10.892	5.253	10.792	2.248
V	1.583	1.577	1.588	1.467	1.527	1.446	1.372	1.490	1.233
Cr	0.672	0.525	0.372	0.526	0.324	0.329	0.401	0.322	0.620
Mn	5.348	10.352	73.989	5.886	25.864	19.082	6.579	7.668	2.466
Fe	0.737	0.793	0.850	0.643	0.799	0.690	0.565	0.775	0.216
Co	0.889	0.754	0.607	0.751	0.511	0.567	0.632	0.323	0.815
Ni	0.805	0.859	0.915	0.714	0.864	0.759	0.637	0.840	0.281
Cu	0.681	0.744	0.810	0.594	0.763	0.650	0.520	0.750	0.368
Zn	0.541	0.609	0.684	0.456	0.640	0.519	0.384	0.637	0.532
Ga	0.676	0.742	0.811	0.593	0.767	0.652	0.522	0.757	0.427
Ge	0.744	0.808	0.874	0.662	0.829	0.719	0.591	0.817	0.384
As	0.647	0.716	0.789	0.567	0.747	0.631	0.499	0.743	0.505
Se	0.751	0.817	0.885	0.673	0.843	0.732	0.605	0.834	0.425
Br	0.683	0.752	0.825	0.606	0.785	0.670	0.540	0.782	0.516
Kr	0.760	0.827	0.897	0.685	0.857	0.746	0.619	0.850	0.460
Rb	0.721	0.791	0.864	0.648	0.825	0.713	0.583	0.823	0.520
Sr	0.725	0.795	0.869	0.653	0.831	0.719	0.590	0.830	0.536
Y	0.781	0.850	0.922	0.710	0.884	0.774	0.648	0.881	0.498
Zr	0.750	0.821	0.896	0.681	0.859	0.748	0.620	0.860	0.548
Nb	0.750	0.822	0.897	0.682	0.862	0.750	0.622	0.864	0.565
Mo	0.733	0.806	0.883	0.666	0.849	0.736	0.607	0.853	0.599
Tc	0.746	0.819	0.897	0.681	0.863	0.751	0.623	0.867	0.603
Ru	0.769	0.842	0.919	0.705	0.886	0.775	0.648	0.890	0.596
Rh	0.686	0.762	0.844	0.624	0.812	0.698	0.568	0.823	0.691
Pd	0.695	0.771	0.853	0.634	0.822	0.708	0.579	0.834	0.697
Ag	0.702	0.778	0.861	0.642	0.831	0.717	0.588	0.843	0.705
Cd	0.759	0.687	0.773	0.649	0.745	0.628	0.550	0.762	0.803
In	0.853	0.719	0.690	0.745	0.663	0.566	0.647	0.684	0.889
Sn	0.743	0.820	0.903	0.686	0.874	0.763	0.635	0.888	0.705
Sb	0.707	0.771	0.856	0.637	0.829	0.715	0.587	0.846	0.765
Te	0.718	0.796	0.881	0.663	0.854	0.742	0.614	0.871	0.753

Supplementary Data- continued

	F	Ne	Na	Mg	Al	Si	P	S	Cl
I	0.744	0.755	0.841	0.640	0.816	0.702	0.573	0.836	0.804
Xe	0.784	0.725	0.812	0.681	0.788	0.673	0.586	0.810	0.842
Cs	0.740	0.778	0.864	0.646	0.840	0.727	0.599	0.861	0.805
Ba	0.778	0.749	0.837	0.676	0.813	0.700	0.583	0.836	0.842
La	0.913	0.988	1.065	0.863	1.038	0.936	0.816	1.048	0.616
Ce	0.809	0.735	0.824	0.710	0.802	0.688	0.618	0.827	0.875
Pr	0.840	0.712	0.801	0.742	0.780	0.666	0.651	0.807	0.906
Nd	0.892	0.969	1.049	0.844	1.025	0.921	0.800	1.039	0.668
Pm	1.123	1.186	1.247	1.073	1.219	1.131	1.027	1.216	0.700
Sm	1.499	1.424	1.337	1.417	1.277	1.308	1.341	1.151	1.415
Eu	0.872	0.951	1.034	0.826	1.011	0.906	0.785	1.029	0.715
Gd	0.823	0.904	0.990	0.779	0.969	0.862	0.738	0.991	0.772
Tb	0.889	0.969	1.051	0.846	1.030	0.926	0.805	1.048	0.715
Dy	1.259	1.311	1.361	1.210	1.332	1.256	1.164	1.321	0.867
Ho	0.936	1.014	1.094	0.893	1.073	0.972	0.853	1.090	0.684
Er	0.925	1.005	1.086	0.883	1.065	0.963	0.844	1.084	0.703
Tm	0.881	0.962	1.046	0.840	1.027	0.923	0.802	1.049	0.755
Yb	1.147	1.213	1.276	1.104	1.252	1.166	1.063	1.255	0.739
Lu	0.896	0.978	1.062	0.857	1.043	0.940	0.820	1.066	0.755
Hf	0.931	1.012	1.094	0.892	1.075	0.974	0.855	1.096	0.727
Ta	0.880	0.963	1.049	0.843	1.032	0.928	0.807	1.057	0.784
W	0.859	0.793	0.886	0.771	0.871	0.761	0.690	0.906	0.953
Re	1.024	1.101	1.178	0.987	1.159	1.064	0.951	1.176	0.651
Os	1.097	1.169	1.240	1.059	1.220	1.130	1.022	1.232	0.690
Ir	1.095	1.168	1.240	1.057	1.220	1.130	1.022	1.232	0.689
Pt	1.052	1.128	1.204	1.015	1.186	1.092	0.981	1.202	0.643
Au	0.944	1.027	1.111	0.910	1.095	0.994	0.877	1.120	0.759
Hg	1.181	1.249	1.313	1.144	1.293	1.209	1.109	1.300	0.789
Tl	1.016	1.096	1.176	0.982	1.159	1.063	0.950	1.180	0.697
Pb	1.051	1.129	1.206	1.017	1.190	1.096	0.985	1.209	0.666
Bi	1.007	1.088	1.169	0.974	1.153	1.057	0.943	1.176	0.718

Supplementary Data- continued

	Ar	K	Ca	Sc	Ti	V	Cr	Mn	Fe
H	3.981	108.277	9.686	2.784	0.955	1.731	71.824	0.924	3.235
D	1.450	1.190	1.078	1.205	4.040	2.038	1.215	4.460	1.098
He	1.061	0.869	0.999	1.407	38.463	2.106	0.901	15.724	1.344
Li	423.937	3.874	3.096	2.116	1.048	1.434	3.968	1.111	2.296
Be	1.243	1.012	0.914	0.904	3.272	1.807	1.078	3.562	0.830
B	1.036	0.765	0.660	1.013	5.322	1.798	0.842	6.418	0.959
C	1.054	0.808	0.710	0.854	3.715	1.708	0.893	4.171	0.799
N	1.101	0.898	0.813	0.635	2.678	1.619	0.989	2.888	0.581
O	0.890	0.633	0.534	0.807	4.374	1.625	0.730	5.114	0.769
F	0.829	0.570	0.471	0.770	4.522	1.583	0.672	5.348	0.737
Ne	0.696	0.419	0.348	0.813	7.471	1.577	0.525	10.352	0.793
Na	0.552	0.272	0.419	0.856	37.146	1.588	0.372	73.989	0.850
Mg	0.676	0.414	0.317	0.664	4.844	1.467	0.526	5.886	0.643
Al	0.449	0.219	0.366	0.797	626.348	1.527	0.324	25.864	0.799
Si	0.495	0.213	0.239	0.695	10.892	1.446	0.329	19.082	0.690
P	0.549	0.284	0.187	0.578	5.253	1.372	0.401	6.579	0.565
S	0.256	0.212	0.357	0.759	10.792	1.490	0.322	7.668	0.775
Cl	0.696	0.501	0.423	0.229	2.248	1.233	0.620	2.466	0.216
Ar	0.000	0.343	0.475	0.808	3.597	1.623	0.454	3.259	0.846
K	0.343	0.000	0.149	0.590	30.711	1.328	0.121	123.470	0.596
Ca	0.475	0.149	0.000	0.471	6.496	1.247	0.219	8.802	0.466
Sc	0.808	0.590	0.471	0.000	1.800	1.110	0.610	1.958	0.237
Ti	3.597	30.711	6.496	1.800	0.000	0.822	35.947	0.105	2.227
V	1.623	1.328	1.247	1.110	0.822	0.000	1.256	0.856	1.146
Cr	0.454	0.121	0.219	0.610	35.947	1.256	0.000	77.547	0.484
Mn	3.259	123.470	8.802	1.958	0.105	0.856	77.547	0.000	2.324
Fe	0.846	0.596	0.466	0.237	2.227	1.146	0.484	2.324	0.000
Co	0.332	0.365	0.456	0.788	6.355	1.443	0.246	5.055	0.602
Ni	0.905	0.666	0.540	0.231	2.122	1.175	0.555	2.192	0.080
Cu	0.837	0.564	0.427	0.389	2.744	1.220	0.454	2.945	0.155
Zn	0.747	0.439	0.294	0.544	4.221	1.276	0.331	4.917	0.321
Ga	0.850	0.570	0.432	0.448	2.956	1.259	0.462	3.204	0.217
Ge	0.902	0.636	0.501	0.408	2.634	1.262	0.528	2.797	0.175
As	0.844	0.553	0.412	0.525	3.388	1.302	0.446	3.755	0.298
Se	0.921	0.652	0.517	0.450	2.731	1.294	0.546	2.912	0.218
Br	0.882	0.595	0.455	0.538	3.276	1.326	0.489	3.601	0.311
Kr	0.940	0.670	0.534	0.486	2.814	1.323	0.565	3.009	0.256
Rb	0.921	0.639	0.501	0.544	3.137	1.347	0.535	3.414	0.317
Sr	0.930	0.647	0.508	0.561	3.186	1.361	0.543	3.474	0.335
Y	0.973	0.702	0.567	0.527	2.873	1.362	0.600	3.074	0.299
Zr	0.958	0.678	0.541	0.575	3.138	1.383	0.577	3.406	0.350
Nb	0.964	0.682	0.545	0.593	3.203	1.396	0.581	3.487	0.369
Mo	0.957	0.670	0.531	0.627	3.411	1.413	0.570	3.753	0.404
Tc	0.971	0.686	0.548	0.631	3.368	1.422	0.587	3.695	0.409
Ru	0.992	0.711	0.574	0.626	3.248	1.428	0.612	3.536	0.404
Rh	0.938	0.638	0.496	0.716	4.159	1.463	0.540	4.755	0.500
Pd	0.949	0.649	0.508	0.724	4.134	1.472	0.553	4.718	0.508
Ag	0.959	0.659	0.518	0.732	4.130	1.481	0.563	4.709	0.517
Cd	0.891	0.573	0.430	0.824	6.173	1.523	0.479	7.859	0.617
In	0.824	0.492	0.493	0.903	12.347	1.569	0.398	23.593	0.705
Sn	1.001	0.707	0.568	0.736	3.856	1.501	0.614	4.323	0.522
Sb	0.968	0.663	0.521	0.793	4.622	1.526	0.570	5.396	0.583
Te	0.990	0.690	0.549	0.783	4.316	1.528	0.598	4.955	0.573

Supplementary Data- continued

	Ar	K	Ca	Sc	Ti	V	Cr	Mn	Fe
I	0.962	0.652	0.510	0.831	5.146	1.551	0.561	6.172	0.625
Xe	0.941	0.625	0.482	0.868	6.061	1.571	0.535	7.636	0.665
Cs	0.986	0.679	0.538	0.835	4.898	1.562	0.590	5.789	0.629
Ba	0.966	0.654	0.512	0.870	5.649	1.581	0.565	6.950	0.668
La	1.144	0.884	0.754	0.659	2.875	1.522	0.795	3.044	0.442
Ce	0.961	0.644	0.502	0.903	6.354	1.604	0.557	8.118	0.704
Pr	0.944	0.623	0.507	0.932	7.478	1.621	0.537	10.181	0.735
Nd	1.141	0.873	0.741	0.712	3.081	1.550	0.787	3.296	0.498
Pm	1.281	1.074	0.963	0.500	2.220	1.515	0.987	2.260	0.545
Sm	0.959	1.173	1.221	1.372	2.083	3.290	1.081	1.971	1.253
Eu	1.137	0.863	0.729	0.760	3.300	1.577	0.779	3.569	0.550
Gd	1.107	0.820	0.685	0.814	3.734	1.596	0.737	4.131	0.607
Tb	1.155	0.883	0.751	0.761	3.239	1.587	0.801	3.488	0.552
Dy	1.368	1.196	1.100	0.675	2.011	1.529	1.113	2.017	0.718
Ho	1.192	0.930	0.801	0.734	3.010	1.589	0.848	3.200	0.523
Er	1.188	0.923	0.793	0.752	3.091	1.598	0.842	3.300	0.543
Tm	1.161	0.885	0.752	0.803	3.410	1.614	0.805	3.702	0.597
Yb	1.325	1.117	1.007	0.544	2.286	1.569	1.037	2.331	0.595
Lu	1.177	0.903	0.771	0.804	3.350	1.623	0.824	3.623	0.598
Hf	1.203	0.937	0.807	0.778	3.145	1.620	0.859	3.364	0.571
Ta	1.171	0.893	0.760	0.833	3.517	1.638	0.816	3.836	0.630
W	1.045	0.731	0.591	0.991	6.377	1.697	0.654	8.097	0.801
Re	1.269	1.025	0.903	0.706	2.740	1.617	0.949	2.864	0.499
Os	1.314	1.089	0.973	0.636	2.498	1.609	1.013	2.575	0.552
Ir	1.316	1.090	0.973	0.645	2.516	1.615	1.014	2.596	0.553
Pt	1.293	1.055	0.934	0.699	2.676	1.628	0.980	2.785	0.508
Au	1.229	0.963	0.834	0.814	3.205	1.655	0.889	3.432	0.611
Hg	1.371	1.168	1.060	0.600	2.312	1.616	1.093	2.356	0.656
Tl	1.279	1.030	0.906	0.755	2.861	1.649	0.957	3.007	0.551
Pb	1.303	1.062	0.941	0.725	2.729	1.647	0.990	2.847	0.521
Bi	1.278	1.026	0.901	0.777	2.931	1.660	0.954	3.091	0.574

Supplementary Data- continued

	Co	Ni	Cu	Zn	Ga	Ge	As	Se	Br
H	5.916	3.071	3.813	5.788	4.045	3.621	4.573	3.710	4.391
D	1.385	1.145	1.006	1.016	0.982	1.041	0.948	1.032	0.954
He	0.996	1.386	1.277	1.146	1.261	1.313	1.223	1.308	1.243
Li	8.241	2.259	2.466	2.823	2.529	2.433	2.645	2.464	2.618
Be	1.257	0.889	0.762	0.921	0.805	0.803	0.868	0.801	0.863
B	1.048	1.017	0.892	0.749	0.880	0.944	0.845	0.945	0.874
C	1.091	0.863	0.732	0.745	0.722	0.788	0.697	0.791	0.718
N	1.168	0.648	0.707	0.866	0.756	0.708	0.824	0.739	0.825
O	0.942	0.835	0.709	0.590	0.703	0.770	0.673	0.776	0.707
F	0.889	0.805	0.681	0.541	0.676	0.744	0.647	0.751	0.683
Ne	0.754	0.859	0.744	0.609	0.742	0.808	0.716	0.817	0.752
Na	0.607	0.915	0.810	0.684	0.811	0.874	0.789	0.885	0.825
Mg	0.751	0.714	0.594	0.456	0.593	0.662	0.567	0.673	0.606
Al	0.511	0.864	0.763	0.640	0.767	0.829	0.747	0.843	0.785
Si	0.567	0.759	0.650	0.519	0.652	0.719	0.631	0.732	0.670
P	0.632	0.637	0.520	0.384	0.522	0.591	0.499	0.605	0.540
S	0.323	0.840	0.750	0.637	0.757	0.817	0.743	0.834	0.782
Cl	0.815	0.281	0.368	0.532	0.427	0.384	0.505	0.425	0.516
Ar	0.332	0.905	0.837	0.747	0.850	0.902	0.844	0.921	0.882
K	0.365	0.666	0.564	0.439	0.570	0.636	0.553	0.652	0.595
Ca	0.456	0.540	0.427	0.294	0.432	0.501	0.412	0.517	0.455
Sc	0.788	0.231	0.389	0.544	0.448	0.408	0.525	0.450	0.538
Ti	6.355	2.122	2.744	4.221	2.956	2.634	3.388	2.731	3.276
V	1.443	1.175	1.220	1.276	1.259	1.262	1.302	1.294	1.326
Cr	0.246	0.555	0.454	0.331	0.462	0.528	0.446	0.546	0.489
Mn	5.055	2.192	2.945	4.917	3.204	2.797	3.755	2.912	3.601
Fe	0.602	0.080	0.155	0.321	0.217	0.175	0.298	0.218	0.311
Co	0.000	0.629	0.548	0.443	0.560	0.618	0.551	0.639	0.593
Ni	0.629	0.000	0.161	0.324	0.222	0.181	0.302	0.224	0.316
Cu	0.548	0.161	0.000	0.169	0.062	0.079	0.144	0.095	0.157
Zn	0.443	0.324	0.169	0.000	0.140	0.213	0.121	0.230	0.166
Ga	0.560	0.222	0.062	0.140	0.000	0.074	0.082	0.091	0.096
Ge	0.618	0.181	0.079	0.213	0.074	0.000	0.124	0.044	0.138
As	0.551	0.302	0.144	0.121	0.082	0.124	0.000	0.110	0.045
Se	0.639	0.224	0.095	0.230	0.091	0.044	0.110	0.000	0.094
Br	0.593	0.316	0.157	0.166	0.096	0.138	0.045	0.094	0.000
Kr	0.659	0.262	0.114	0.249	0.109	0.082	0.129	0.039	0.084
Rb	0.636	0.323	0.164	0.215	0.102	0.144	0.094	0.101	0.049
Sr	0.646	0.341	0.182	0.223	0.120	0.162	0.103	0.119	0.057
Y	0.695	0.305	0.149	0.285	0.145	0.126	0.165	0.082	0.120
Zr	0.678	0.356	0.197	0.258	0.136	0.178	0.138	0.135	0.093
Nb	0.684	0.375	0.216	0.263	0.155	0.198	0.143	0.154	0.098
Mo	0.676	0.411	0.253	0.250	0.192	0.234	0.130	0.191	0.097
Tc	0.693	0.416	0.258	0.268	0.197	0.239	0.148	0.196	0.103
Ru	0.716	0.411	0.252	0.295	0.191	0.234	0.176	0.191	0.131
Rh	0.655	0.506	0.351	0.217	0.291	0.333	0.210	0.290	0.197
Pd	0.667	0.514	0.359	0.230	0.299	0.341	0.218	0.298	0.205
Ag	0.678	0.523	0.369	0.242	0.309	0.350	0.228	0.308	0.215
Cd	0.603	0.621	0.473	0.308	0.414	0.454	0.335	0.412	0.322
In	0.530	0.707	0.566	0.406	0.509	0.546	0.431	0.505	0.418
Sn	0.727	0.529	0.373	0.296	0.313	0.356	0.233	0.313	0.220
Sb	0.690	0.589	0.437	0.269	0.378	0.419	0.297	0.377	0.285
Te	0.716	0.580	0.426	0.279	0.367	0.409	0.287	0.367	0.274

Supplementary Data- continued

	Co	Ni	Cu	Zn	Ga	Ge	As	Se	Br
I	0.684	0.631	0.480	0.314	0.422	0.463	0.342	0.421	0.330
Xe	0.661	0.671	0.523	0.357	0.465	0.505	0.386	0.464	0.374
Cs	0.712	0.636	0.484	0.318	0.426	0.468	0.347	0.426	0.335
Ba	0.691	0.674	0.525	0.359	0.467	0.508	0.388	0.467	0.376
La	0.893	0.452	0.358	0.494	0.357	0.284	0.379	0.269	0.335
Ce	0.685	0.709	0.563	0.398	0.505	0.546	0.427	0.505	0.415
Pr	0.668	0.741	0.596	0.433	0.539	0.579	0.462	0.539	0.450
Nd	0.890	0.509	0.350	0.484	0.346	0.336	0.368	0.294	0.325
Pm	1.056	0.471	0.596	0.719	0.593	0.524	0.612	0.509	0.570
Sm	0.907	1.234	1.175	1.101	1.136	1.141	1.088	1.109	1.068
Eu	0.887	0.560	0.403	0.474	0.345	0.389	0.359	0.348	0.316
Gd	0.853	0.617	0.462	0.429	0.405	0.448	0.326	0.408	0.315
Tb	0.908	0.563	0.405	0.499	0.360	0.392	0.384	0.352	0.341
Dy	1.163	0.647	0.762	0.872	0.758	0.694	0.773	0.679	0.733
Ho	0.951	0.536	0.414	0.552	0.415	0.364	0.438	0.329	0.396
Er	0.947	0.555	0.407	0.544	0.407	0.385	0.431	0.345	0.389
Tm	0.917	0.609	0.452	0.503	0.395	0.439	0.389	0.400	0.347
Yb	1.110	0.522	0.648	0.773	0.647	0.578	0.667	0.565	0.626
Lu	0.936	0.611	0.454	0.525	0.398	0.442	0.411	0.402	0.369
Hf	0.966	0.584	0.427	0.563	0.426	0.415	0.450	0.376	0.408
Ta	0.930	0.643	0.487	0.515	0.430	0.475	0.402	0.436	0.360
W	0.788	0.810	0.664	0.501	0.609	0.651	0.534	0.613	0.524
Re	1.045	0.513	0.530	0.665	0.531	0.460	0.555	0.448	0.514
Os	1.098	0.481	0.610	0.740	0.610	0.541	0.633	0.528	0.593
Ir	1.100	0.481	0.610	0.741	0.611	0.541	0.634	0.529	0.594
Pt	1.074	0.507	0.567	0.701	0.568	0.498	0.592	0.486	0.552
Au	0.998	0.626	0.469	0.596	0.459	0.459	0.485	0.420	0.444
Hg	1.167	0.586	0.712	0.836	0.712	0.644	0.733	0.632	0.694
Tl	1.058	0.566	0.537	0.674	0.539	0.468	0.564	0.457	0.524
Pb	1.086	0.536	0.576	0.711	0.578	0.508	0.603	0.496	0.563
Bi	1.057	0.589	0.532	0.670	0.535	0.464	0.560	0.453	0.520

Supplementary Data- continued

	Kr	Rb	Sr	Y	Zr	Nb	Mo	Tc	Ru
H	3.783	4.179	4.228	3.814	4.137	4.208	4.466	4.398	4.228
D	1.025	0.978	0.974	1.025	0.987	0.980	0.957	0.964	0.982
He	1.306	1.267	1.266	1.310	1.279	1.275	1.255	1.263	1.279
Li	2.489	2.582	2.596	2.507	2.583	2.601	2.656	2.645	2.613
Be	0.801	0.852	0.862	0.817	0.860	0.872	0.900	0.898	0.885
B	0.948	0.906	0.907	0.960	0.927	0.925	0.905	0.916	0.936
C	0.795	0.753	0.755	0.810	0.776	0.774	0.755	0.767	0.788
N	0.765	0.820	0.832	0.790	0.835	0.849	0.879	0.878	0.868
O	0.784	0.744	0.747	0.803	0.771	0.771	0.753	0.765	0.788
F	0.760	0.721	0.725	0.781	0.750	0.750	0.733	0.746	0.769
Ne	0.827	0.791	0.795	0.850	0.821	0.822	0.806	0.819	0.842
Na	0.897	0.864	0.869	0.922	0.896	0.897	0.883	0.897	0.919
Mg	0.685	0.648	0.653	0.710	0.681	0.682	0.666	0.681	0.705
Al	0.857	0.825	0.831	0.884	0.859	0.862	0.849	0.863	0.886
Si	0.746	0.713	0.719	0.774	0.748	0.750	0.736	0.751	0.775
P	0.619	0.583	0.590	0.648	0.620	0.622	0.607	0.623	0.648
S	0.850	0.823	0.830	0.881	0.860	0.864	0.853	0.867	0.890
Cl	0.460	0.520	0.536	0.498	0.548	0.565	0.599	0.603	0.596
Ar	0.940	0.921	0.930	0.973	0.958	0.964	0.957	0.971	0.992
K	0.670	0.639	0.647	0.702	0.678	0.682	0.670	0.686	0.711
Ca	0.534	0.501	0.508	0.567	0.541	0.545	0.531	0.548	0.574
Sc	0.486	0.544	0.561	0.527	0.575	0.593	0.627	0.631	0.626
Ti	2.814	3.137	3.186	2.873	3.138	3.203	3.411	3.368	3.248
V	1.323	1.347	1.361	1.362	1.383	1.396	1.413	1.422	1.428
Cr	0.565	0.535	0.543	0.600	0.577	0.581	0.570	0.587	0.612
Mn	3.009	3.414	3.474	3.074	3.406	3.487	3.753	3.695	3.536
Fe	0.256	0.317	0.335	0.299	0.350	0.369	0.404	0.409	0.404
Co	0.659	0.636	0.646	0.695	0.678	0.684	0.676	0.693	0.716
Ni	0.262	0.323	0.341	0.305	0.356	0.375	0.411	0.416	0.411
Cu	0.114	0.164	0.182	0.149	0.197	0.216	0.253	0.258	0.252
Zn	0.249	0.215	0.223	0.285	0.258	0.263	0.250	0.268	0.295
Ga	0.109	0.102	0.120	0.145	0.136	0.155	0.192	0.197	0.191
Ge	0.082	0.144	0.162	0.126	0.178	0.198	0.234	0.239	0.234
As	0.129	0.094	0.103	0.165	0.138	0.143	0.130	0.148	0.176
Se	0.039	0.101	0.119	0.082	0.135	0.154	0.191	0.196	0.191
Br	0.084	0.049	0.057	0.120	0.093	0.098	0.097	0.103	0.131
Kr	0.000	0.062	0.080	0.044	0.096	0.116	0.152	0.158	0.153
Rb	0.062	0.000	0.018	0.071	0.044	0.054	0.090	0.096	0.091
Sr	0.080	0.018	0.000	0.062	0.036	0.040	0.072	0.078	0.074
Y	0.044	0.071	0.062	0.000	0.052	0.072	0.109	0.114	0.109
Zr	0.096	0.044	0.036	0.052	0.000	0.020	0.056	0.062	0.057
Nb	0.116	0.054	0.040	0.072	0.020	0.000	0.037	0.042	0.037
Mo	0.152	0.090	0.072	0.109	0.056	0.037	0.000	0.018	0.046
Tc	0.158	0.096	0.078	0.114	0.062	0.042	0.018	0.000	0.028
Ru	0.153	0.091	0.074	0.109	0.057	0.037	0.046	0.028	0.000
Rh	0.252	0.191	0.173	0.209	0.157	0.137	0.101	0.095	0.100
Pd	0.260	0.199	0.181	0.217	0.165	0.146	0.109	0.104	0.109
Ag	0.270	0.209	0.191	0.227	0.175	0.155	0.119	0.113	0.119
Cd	0.375	0.315	0.297	0.332	0.281	0.262	0.226	0.220	0.225
In	0.468	0.411	0.393	0.426	0.377	0.358	0.323	0.317	0.321
Sn	0.276	0.214	0.196	0.233	0.181	0.161	0.125	0.119	0.125
Sb	0.340	0.279	0.261	0.297	0.246	0.226	0.190	0.185	0.190
Te	0.330	0.269	0.251	0.287	0.235	0.216	0.179	0.174	0.179

Supplementary Data- continued

	Kr	Rb	Sr	Y	Zr	Nb	Mo	Tc	Ru
I	0.384	0.324	0.306	0.342	0.291	0.271	0.235	0.230	0.235
Xe	0.427	0.367	0.350	0.385	0.334	0.315	0.279	0.274	0.279
Cs	0.389	0.329	0.311	0.347	0.296	0.277	0.241	0.235	0.240
Ba	0.430	0.370	0.353	0.388	0.338	0.318	0.282	0.277	0.282
La	0.253	0.288	0.280	0.218	0.245	0.241	0.253	0.236	0.208
Ce	0.469	0.410	0.392	0.427	0.377	0.358	0.322	0.317	0.322
Pr	0.503	0.444	0.427	0.461	0.412	0.393	0.357	0.352	0.357
Nd	0.258	0.278	0.270	0.216	0.236	0.231	0.244	0.226	0.199
Pm	0.492	0.525	0.517	0.458	0.483	0.478	0.489	0.472	0.446
Sm	1.079	1.050	1.035	1.041	1.015	1.000	0.979	0.970	0.965
Eu	0.312	0.269	0.262	0.271	0.227	0.223	0.236	0.219	0.191
Gd	0.372	0.311	0.293	0.330	0.279	0.260	0.224	0.219	0.224
Tb	0.315	0.295	0.287	0.274	0.253	0.249	0.262	0.245	0.217
Dy	0.662	0.690	0.682	0.628	0.650	0.645	0.654	0.637	0.612
Ho	0.314	0.349	0.342	0.281	0.308	0.304	0.317	0.300	0.272
Er	0.309	0.342	0.335	0.274	0.301	0.297	0.310	0.293	0.266
Tm	0.364	0.303	0.293	0.324	0.272	0.256	0.269	0.252	0.224
Yb	0.549	0.582	0.574	0.516	0.542	0.537	0.549	0.532	0.506
Lu	0.367	0.323	0.316	0.327	0.283	0.279	0.292	0.275	0.247
Hf	0.340	0.362	0.355	0.301	0.322	0.318	0.331	0.314	0.287
Ta	0.401	0.340	0.323	0.361	0.310	0.291	0.284	0.267	0.256
W	0.578	0.520	0.503	0.539	0.490	0.471	0.436	0.431	0.437
Re	0.433	0.469	0.462	0.401	0.429	0.425	0.438	0.421	0.394
Os	0.513	0.548	0.541	0.482	0.509	0.505	0.517	0.500	0.474
Ir	0.514	0.549	0.542	0.483	0.510	0.506	0.518	0.502	0.475
Pt	0.471	0.507	0.500	0.440	0.468	0.464	0.477	0.460	0.433
Au	0.386	0.399	0.392	0.347	0.360	0.356	0.370	0.353	0.325
Hg	0.617	0.651	0.644	0.586	0.612	0.608	0.619	0.603	0.577
Tl	0.443	0.479	0.473	0.412	0.440	0.437	0.450	0.433	0.406
Pb	0.482	0.518	0.512	0.452	0.480	0.476	0.489	0.473	0.446
Bi	0.439	0.476	0.469	0.409	0.437	0.434	0.447	0.430	0.404

Supplementary Data- continued

	Rh	Pd	Ag	Cd	In	Sn	Sb	Te	I
H	5.449	5.402	5.384	8.571	24.899	4.969	6.046	5.591	6.816
D	1.020	1.018	1.018	1.115	1.203	0.995	1.051	1.032	1.079
He	1.202	1.206	1.208	1.118	1.031	1.236	1.186	1.206	1.164
Li	2.830	2.825	2.825	3.162	3.640	2.765	2.924	2.865	3.017
Be	0.976	0.977	0.979	1.077	1.163	0.964	1.021	1.004	1.052
B	0.852	0.858	0.863	0.854	0.947	0.898	0.846	0.870	0.829
C	0.826	0.828	0.831	0.932	1.023	0.818	0.878	0.861	0.911
N	0.959	0.962	0.966	1.061	1.144	0.956	1.012	0.998	1.045
O	0.705	0.713	0.719	0.802	0.895	0.759	0.749	0.734	0.785
F	0.686	0.695	0.702	0.759	0.853	0.743	0.707	0.718	0.744
Ne	0.762	0.771	0.778	0.687	0.719	0.820	0.771	0.796	0.755
Na	0.844	0.853	0.861	0.773	0.690	0.903	0.856	0.881	0.841
Mg	0.624	0.634	0.642	0.649	0.745	0.686	0.637	0.663	0.640
Al	0.812	0.822	0.831	0.745	0.663	0.874	0.829	0.854	0.816
Si	0.698	0.708	0.717	0.628	0.566	0.763	0.715	0.742	0.702
P	0.568	0.579	0.588	0.550	0.647	0.635	0.587	0.614	0.573
S	0.823	0.834	0.843	0.762	0.684	0.888	0.846	0.871	0.836
Cl	0.691	0.697	0.705	0.803	0.889	0.705	0.765	0.753	0.804
Ar	0.938	0.949	0.959	0.891	0.824	1.001	0.968	0.990	0.962
K	0.638	0.649	0.659	0.573	0.492	0.707	0.663	0.690	0.652
Ca	0.496	0.508	0.518	0.430	0.493	0.568	0.521	0.549	0.510
Sc	0.716	0.724	0.732	0.824	0.903	0.736	0.793	0.783	0.831
Ti	4.159	4.134	4.130	6.173	12.347	3.856	4.622	4.316	5.146
V	1.463	1.472	1.481	1.523	1.569	1.501	1.526	1.528	1.551
Cr	0.540	0.553	0.563	0.479	0.398	0.614	0.570	0.598	0.561
Mn	4.755	4.718	4.709	7.859	23.593	4.323	5.396	4.955	6.172
Fe	0.500	0.508	0.517	0.617	0.705	0.522	0.583	0.573	0.625
Co	0.655	0.667	0.678	0.603	0.530	0.727	0.690	0.716	0.684
Ni	0.506	0.514	0.523	0.621	0.707	0.529	0.589	0.580	0.631
Cu	0.351	0.359	0.369	0.473	0.566	0.373	0.437	0.426	0.480
Zn	0.217	0.230	0.242	0.308	0.406	0.296	0.269	0.279	0.314
Ga	0.291	0.299	0.309	0.414	0.509	0.313	0.378	0.367	0.422
Ge	0.333	0.341	0.350	0.454	0.546	0.356	0.419	0.409	0.463
As	0.210	0.218	0.228	0.335	0.431	0.233	0.297	0.287	0.342
Se	0.290	0.298	0.308	0.412	0.505	0.313	0.377	0.367	0.421
Br	0.197	0.205	0.215	0.322	0.418	0.220	0.285	0.274	0.330
Kr	0.252	0.260	0.270	0.375	0.468	0.276	0.340	0.330	0.384
Rb	0.191	0.199	0.209	0.315	0.411	0.214	0.279	0.269	0.324
Sr	0.173	0.181	0.191	0.297	0.393	0.196	0.261	0.251	0.306
Y	0.209	0.217	0.227	0.332	0.426	0.233	0.297	0.287	0.342
Zr	0.157	0.165	0.175	0.281	0.377	0.181	0.246	0.235	0.291
Nb	0.137	0.146	0.155	0.262	0.358	0.161	0.226	0.216	0.271
Mo	0.101	0.109	0.119	0.226	0.323	0.125	0.190	0.179	0.235
Tc	0.095	0.104	0.113	0.220	0.317	0.119	0.185	0.174	0.230
Ru	0.100	0.109	0.119	0.225	0.321	0.125	0.190	0.179	0.235
Rh	0.000	0.014	0.025	0.126	0.225	0.081	0.090	0.079	0.135
Pd	0.014	0.000	0.012	0.118	0.217	0.068	0.081	0.071	0.127
Ag	0.025	0.012	0.000	0.108	0.207	0.056	0.071	0.061	0.117
Cd	0.126	0.118	0.108	0.000	0.100	0.143	0.097	0.127	0.090
In	0.225	0.217	0.207	0.100	0.000	0.220	0.176	0.206	0.169
Sn	0.081	0.068	0.056	0.143	0.220	0.000	0.065	0.055	0.111
Sb	0.090	0.081	0.071	0.097	0.176	0.065	0.000	0.030	0.046
Te	0.079	0.071	0.061	0.127	0.206	0.055	0.030	0.000	0.056

Supplementary Data- continued

	Rh	Pd	Ag	Cd	In	Sn	Sb	Te	I
I	0.135	0.127	0.117	0.090	0.169	0.111	0.046	0.056	0.000
Xe	0.180	0.171	0.162	0.064	0.144	0.156	0.091	0.101	0.045
Cs	0.141	0.132	0.123	0.121	0.201	0.117	0.051	0.062	0.032
Ba	0.183	0.175	0.165	0.097	0.177	0.159	0.094	0.104	0.048
La	0.285	0.271	0.260	0.343	0.415	0.205	0.249	0.220	0.255
Ce	0.223	0.215	0.205	0.097	0.171	0.199	0.134	0.145	0.089
Pr	0.259	0.251	0.241	0.133	0.152	0.235	0.170	0.181	0.125
Nd	0.276	0.263	0.251	0.336	0.409	0.196	0.241	0.212	0.248
Pm	0.515	0.502	0.490	0.562	0.621	0.438	0.476	0.449	0.480
Sm	0.919	0.910	0.900	0.845	0.788	0.879	0.846	0.845	0.815
Eu	0.269	0.256	0.244	0.330	0.405	0.189	0.234	0.205	0.241
Gd	0.224	0.211	0.200	0.286	0.363	0.144	0.190	0.160	0.198
Tb	0.295	0.282	0.270	0.355	0.430	0.215	0.260	0.231	0.268
Dy	0.673	0.660	0.649	0.710	0.760	0.600	0.632	0.608	0.633
Ho	0.350	0.337	0.325	0.409	0.482	0.271	0.315	0.286	0.322
Er	0.343	0.330	0.319	0.403	0.477	0.264	0.309	0.280	0.316
Tm	0.302	0.289	0.278	0.364	0.439	0.223	0.269	0.239	0.276
Yb	0.575	0.562	0.551	0.623	0.684	0.499	0.538	0.511	0.542
Lu	0.326	0.313	0.301	0.387	0.462	0.247	0.292	0.263	0.300
Hf	0.365	0.352	0.341	0.425	0.499	0.286	0.331	0.302	0.338
Ta	0.318	0.305	0.294	0.380	0.456	0.239	0.285	0.255	0.292
W	0.339	0.331	0.322	0.214	0.292	0.317	0.252	0.263	0.207
Re	0.470	0.457	0.446	0.527	0.597	0.393	0.436	0.408	0.443
Os	0.547	0.534	0.523	0.600	0.665	0.471	0.512	0.484	0.517
Ir	0.549	0.536	0.525	0.602	0.668	0.473	0.514	0.486	0.520
Pt	0.509	0.496	0.485	0.565	0.633	0.432	0.475	0.447	0.481
Au	0.404	0.391	0.380	0.465	0.539	0.326	0.371	0.342	0.379
Hg	0.647	0.634	0.624	0.695	0.755	0.573	0.611	0.585	0.615
Tl	0.483	0.471	0.460	0.542	0.613	0.407	0.451	0.422	0.458
Pb	0.522	0.509	0.499	0.579	0.648	0.446	0.489	0.461	0.496
Bi	0.481	0.468	0.457	0.540	0.612	0.404	0.449	0.420	0.456

Supplementary Data- continued

	Xe	Cs	Ba	La	Ce	Pr	Nd	Pm	Sm
H	8.296	6.413	7.583	3.627	8.758	10.858	3.860	2.812	2.513
D	1.115	1.068	1.101	1.071	1.125	1.153	1.038	1.275	1.754
He	1.132	1.179	1.148	1.365	1.128	1.103	1.340	1.525	1.543
Li	3.153	2.976	3.097	2.499	3.194	3.321	2.561	2.261	3.362
Be	1.088	1.044	1.078	0.897	1.105	1.133	0.881	1.113	1.693
B	0.868	0.845	0.858	1.056	0.887	0.917	1.030	1.256	1.588
C	0.949	0.905	0.941	0.917	0.970	0.999	0.891	1.130	1.609
N	1.081	1.041	1.075	0.845	1.103	1.131	0.889	0.942	1.640
O	0.824	0.780	0.817	0.927	0.848	0.879	0.905	1.137	1.529
F	0.784	0.740	0.778	0.913	0.809	0.840	0.892	1.123	1.499
Ne	0.725	0.778	0.749	0.988	0.735	0.712	0.969	1.186	1.424
Na	0.812	0.864	0.837	1.065	0.824	0.801	1.049	1.247	1.337
Mg	0.681	0.646	0.676	0.863	0.710	0.742	0.844	1.073	1.417
Al	0.788	0.840	0.813	1.038	0.802	0.780	1.025	1.219	1.277
Si	0.673	0.727	0.700	0.936	0.688	0.666	0.921	1.131	1.308
P	0.586	0.599	0.583	0.816	0.618	0.651	0.800	1.027	1.341
S	0.810	0.861	0.836	1.048	0.827	0.807	1.039	1.216	1.151
Cl	0.842	0.805	0.842	0.616	0.875	0.906	0.668	0.700	1.415
Ar	0.941	0.986	0.966	1.144	0.961	0.944	1.141	1.281	0.959
K	0.625	0.679	0.654	0.884	0.644	0.623	0.873	1.074	1.173
Ca	0.482	0.538	0.512	0.754	0.502	0.507	0.741	0.963	1.221
Sc	0.868	0.835	0.870	0.659	0.903	0.932	0.712	0.500	1.372
Ti	6.061	4.898	5.649	2.875	6.354	7.478	3.081	2.220	2.083
V	1.571	1.562	1.581	1.522	1.604	1.621	1.550	1.515	3.290
Cr	0.535	0.590	0.565	0.795	0.557	0.537	0.787	0.987	1.081
Mn	7.636	5.789	6.950	3.044	8.118	10.181	3.296	2.260	1.971
Fe	0.665	0.629	0.668	0.442	0.704	0.735	0.498	0.545	1.253
Co	0.661	0.712	0.691	0.893	0.685	0.668	0.890	1.056	0.907
Ni	0.671	0.636	0.674	0.452	0.709	0.741	0.509	0.471	1.234
Cu	0.523	0.484	0.525	0.358	0.563	0.596	0.350	0.596	1.175
Zn	0.357	0.318	0.359	0.494	0.398	0.433	0.484	0.719	1.101
Ga	0.465	0.426	0.467	0.357	0.505	0.539	0.346	0.593	1.136
Ge	0.505	0.468	0.508	0.284	0.546	0.579	0.336	0.524	1.141
As	0.386	0.347	0.388	0.379	0.427	0.462	0.368	0.612	1.088
Se	0.464	0.426	0.467	0.269	0.505	0.539	0.294	0.509	1.109
Br	0.374	0.335	0.376	0.335	0.415	0.450	0.325	0.570	1.068
Kr	0.427	0.389	0.430	0.253	0.469	0.503	0.258	0.492	1.079
Rb	0.367	0.329	0.370	0.288	0.410	0.444	0.278	0.525	1.050
Sr	0.350	0.311	0.353	0.280	0.392	0.427	0.270	0.517	1.035
Y	0.385	0.347	0.388	0.218	0.427	0.461	0.216	0.458	1.041
Zr	0.334	0.296	0.338	0.245	0.377	0.412	0.236	0.483	1.015
Nb	0.315	0.277	0.318	0.241	0.358	0.393	0.231	0.478	1.000
Mo	0.279	0.241	0.282	0.253	0.322	0.357	0.244	0.489	0.979
Tc	0.274	0.235	0.277	0.236	0.317	0.352	0.226	0.472	0.970
Ru	0.279	0.240	0.282	0.208	0.322	0.357	0.199	0.446	0.965
Rh	0.180	0.141	0.183	0.285	0.223	0.259	0.276	0.515	0.919
Pd	0.171	0.132	0.175	0.271	0.215	0.251	0.263	0.502	0.910
Ag	0.162	0.123	0.165	0.260	0.205	0.241	0.251	0.490	0.900
Cd	0.064	0.121	0.097	0.343	0.097	0.133	0.336	0.562	0.845
In	0.144	0.201	0.177	0.415	0.171	0.152	0.409	0.621	0.788
Sn	0.156	0.117	0.159	0.205	0.199	0.235	0.196	0.438	0.879
Sb	0.091	0.051	0.094	0.249	0.134	0.170	0.241	0.476	0.846
Te	0.101	0.062	0.104	0.220	0.145	0.181	0.212	0.449	0.845

Supplementary Data- continued

	Xe	Cs	Ba	La	Ce	Pr	Nd	Pm	Sm
I	0.045	0.032	0.048	0.255	0.089	0.125	0.248	0.480	0.815
Xe	0.000	0.058	0.033	0.279	0.044	0.080	0.272	0.499	0.789
Cs	0.058	0.000	0.042	0.224	0.083	0.119	0.217	0.450	0.803
Ba	0.033	0.042	0.000	0.247	0.041	0.077	0.240	0.469	0.778
La	0.279	0.224	0.247	0.000	0.269	0.303	0.060	0.243	0.865
Ce	0.044	0.083	0.041	0.269	0.000	0.036	0.244	0.470	0.750
Pr	0.080	0.119	0.077	0.303	0.036	0.000	0.262	0.483	0.727
Nd	0.272	0.217	0.240	0.060	0.244	0.262	0.000	0.250	0.828
Pm	0.499	0.450	0.469	0.243	0.470	0.483	0.250	0.000	0.889
Sm	0.789	0.803	0.778	0.865	0.750	0.727	0.828	0.889	0.000
Eu	0.266	0.210	0.234	0.116	0.239	0.257	0.056	0.288	0.808
Gd	0.223	0.166	0.190	0.176	0.196	0.214	0.116	0.343	0.797
Tb	0.292	0.236	0.260	0.121	0.265	0.283	0.061	0.293	0.828
Dy	0.649	0.605	0.620	0.418	0.619	0.630	0.422	0.185	0.941
Ho	0.346	0.291	0.314	0.095	0.319	0.336	0.076	0.270	0.857
Er	0.341	0.285	0.308	0.116	0.313	0.330	0.069	0.290	0.860
Tm	0.301	0.245	0.269	0.172	0.274	0.292	0.112	0.343	0.850
Yb	0.562	0.513	0.532	0.305	0.533	0.547	0.313	0.075	0.940
Lu	0.325	0.269	0.292	0.176	0.297	0.315	0.116	0.348	0.868
Hf	0.363	0.308	0.331	0.150	0.336	0.353	0.092	0.324	0.886
Ta	0.318	0.261	0.285	0.211	0.291	0.309	0.151	0.381	0.874
W	0.162	0.202	0.160	0.388	0.123	0.142	0.330	0.540	0.806
Re	0.466	0.412	0.434	0.191	0.438	0.455	0.201	0.259	0.935
Os	0.539	0.488	0.509	0.273	0.511	0.526	0.281	0.191	0.962
Ir	0.542	0.490	0.511	0.275	0.514	0.529	0.283	0.202	0.968
Pt	0.504	0.451	0.473	0.232	0.477	0.493	0.241	0.257	0.960
Au	0.404	0.348	0.372	0.200	0.377	0.395	0.141	0.374	0.931
Hg	0.635	0.587	0.606	0.381	0.607	0.621	0.388	0.138	1.008
Tl	0.482	0.428	0.450	0.205	0.455	0.471	0.215	0.320	0.966
Pb	0.519	0.466	0.488	0.246	0.492	0.508	0.255	0.292	0.982
Bi	0.480	0.426	0.449	0.203	0.453	0.470	0.213	0.345	0.973

Supplementary Data- continued

	Eu	Gd	Tb	Dy	Ho	Er	Tm	Yb	Lu
H	4.117	4.678	4.024	2.538	3.721	3.817	4.216	2.832	4.127
D	1.008	0.982	1.020	1.404	1.062	1.048	1.000	1.273	1.011
He	1.316	1.271	1.328	1.618	1.364	1.353	1.312	1.529	1.322
Li	2.623	2.737	2.605	2.166	2.536	2.561	2.652	2.279	2.635
Be	0.919	0.973	0.911	1.255	0.901	0.889	0.939	1.121	0.932
B	1.006	0.957	1.021	1.382	1.065	1.053	1.008	1.269	1.021
C	0.867	0.840	0.883	1.269	0.929	0.917	0.871	1.145	0.885
N	0.929	0.983	0.924	1.095	0.889	0.905	0.955	0.959	0.950
O	0.883	0.834	0.900	1.273	0.946	0.936	0.891	1.158	0.906
F	0.872	0.823	0.889	1.259	0.936	0.925	0.881	1.147	0.896
Ne	0.951	0.904	0.969	1.311	1.014	1.005	0.962	1.213	0.978
Na	1.034	0.990	1.051	1.361	1.094	1.086	1.046	1.276	1.062
Mg	0.826	0.779	0.846	1.210	0.893	0.883	0.840	1.104	0.857
Al	1.011	0.969	1.030	1.332	1.073	1.065	1.027	1.252	1.043
Si	0.906	0.862	0.926	1.256	0.972	0.963	0.923	1.166	0.940
P	0.785	0.738	0.805	1.164	0.853	0.844	0.802	1.063	0.820
S	1.029	0.991	1.048	1.321	1.090	1.084	1.049	1.255	1.066
Cl	0.715	0.772	0.715	0.867	0.684	0.703	0.755	0.739	0.755
Ar	1.137	1.107	1.155	1.368	1.192	1.188	1.161	1.325	1.177
K	0.863	0.820	0.883	1.196	0.930	0.923	0.885	1.117	0.903
Ca	0.729	0.685	0.751	1.100	0.801	0.793	0.752	1.007	0.771
Sc	0.760	0.814	0.761	0.675	0.734	0.752	0.803	0.544	0.804
Ti	3.300	3.734	3.239	2.011	3.010	3.091	3.410	2.286	3.350
V	1.577	1.596	1.587	1.529	1.589	1.598	1.614	1.569	1.623
Cr	0.779	0.737	0.801	1.113	0.848	0.842	0.805	1.037	0.824
Mn	3.569	4.131	3.488	2.017	3.200	3.300	3.702	2.331	3.623
Fe	0.550	0.607	0.552	0.718	0.523	0.543	0.597	0.595	0.598
Co	0.887	0.853	0.908	1.163	0.951	0.947	0.917	1.110	0.936
Ni	0.560	0.617	0.563	0.647	0.536	0.555	0.609	0.522	0.611
Cu	0.403	0.462	0.405	0.762	0.414	0.407	0.452	0.648	0.454
Zn	0.474	0.429	0.499	0.872	0.552	0.544	0.503	0.773	0.525
Ga	0.345	0.405	0.360	0.758	0.415	0.407	0.395	0.647	0.398
Ge	0.389	0.448	0.392	0.694	0.364	0.385	0.439	0.578	0.442
As	0.359	0.326	0.384	0.773	0.438	0.431	0.389	0.667	0.411
Se	0.348	0.408	0.352	0.679	0.329	0.345	0.400	0.565	0.402
Br	0.316	0.315	0.341	0.733	0.396	0.389	0.347	0.626	0.369
Kr	0.312	0.372	0.315	0.662	0.314	0.309	0.364	0.549	0.367
Rb	0.269	0.311	0.295	0.690	0.349	0.342	0.303	0.582	0.323
Sr	0.262	0.293	0.287	0.682	0.342	0.335	0.293	0.574	0.316
Y	0.271	0.330	0.274	0.628	0.281	0.274	0.324	0.516	0.327
Zr	0.227	0.279	0.253	0.650	0.308	0.301	0.272	0.542	0.283
Nb	0.223	0.260	0.249	0.645	0.304	0.297	0.256	0.537	0.279
Mo	0.236	0.224	0.262	0.654	0.317	0.310	0.269	0.549	0.292
Tc	0.219	0.219	0.245	0.637	0.300	0.293	0.252	0.532	0.275
Ru	0.191	0.224	0.217	0.612	0.272	0.266	0.224	0.506	0.247
Rh	0.269	0.224	0.295	0.673	0.350	0.343	0.302	0.575	0.326
Pd	0.256	0.211	0.282	0.660	0.337	0.330	0.289	0.562	0.313
Ag	0.244	0.200	0.270	0.649	0.325	0.319	0.278	0.551	0.301
Cd	0.330	0.286	0.355	0.710	0.409	0.403	0.364	0.623	0.387
In	0.405	0.363	0.430	0.760	0.482	0.477	0.439	0.684	0.462
Sn	0.189	0.144	0.215	0.600	0.271	0.264	0.223	0.499	0.247
Sb	0.234	0.190	0.260	0.632	0.315	0.309	0.269	0.538	0.292
Te	0.205	0.160	0.231	0.608	0.286	0.280	0.239	0.511	0.263

Supplementary Data- continued

	Eu	Gd	Tb	Dy	Ho	Er	Tm	Yb	Lu
I	0.241	0.198	0.268	0.633	0.322	0.316	0.276	0.542	0.300
Xe	0.266	0.223	0.292	0.649	0.346	0.341	0.301	0.562	0.325
Cs	0.210	0.166	0.236	0.605	0.291	0.285	0.245	0.513	0.269
Ba	0.234	0.190	0.260	0.620	0.314	0.308	0.269	0.532	0.292
La	0.116	0.176	0.121	0.418	0.095	0.116	0.172	0.305	0.176
Ce	0.239	0.196	0.265	0.619	0.319	0.313	0.274	0.533	0.297
Pr	0.257	0.214	0.283	0.630	0.336	0.330	0.292	0.547	0.315
Nd	0.056	0.116	0.061	0.422	0.076	0.069	0.112	0.313	0.116
Pm	0.288	0.343	0.293	0.185	0.270	0.290	0.343	0.075	0.348
Sm	0.808	0.797	0.828	0.941	0.857	0.860	0.850	0.940	0.868
Eu	0.000	0.060	0.027	0.425	0.083	0.076	0.056	0.318	0.060
Gd	0.060	0.000	0.071	0.460	0.127	0.121	0.080	0.358	0.104
Tb	0.027	0.071	0.000	0.400	0.056	0.050	0.051	0.292	0.056
Dy	0.425	0.460	0.400	0.000	0.364	0.384	0.432	0.182	0.438
Ho	0.083	0.127	0.056	0.364	0.000	0.021	0.077	0.238	0.082
Er	0.076	0.121	0.050	0.384	0.021	0.000	0.056	0.244	0.060
Tm	0.056	0.080	0.051	0.432	0.077	0.056	0.000	0.282	0.024
Yb	0.318	0.358	0.292	0.182	0.238	0.244	0.282	0.000	0.273
Lu	0.060	0.104	0.056	0.438	0.082	0.060	0.024	0.273	0.000
Hf	0.099	0.143	0.072	0.417	0.056	0.034	0.064	0.249	0.040
Ta	0.095	0.096	0.091	0.470	0.117	0.095	0.040	0.306	0.035
W	0.276	0.217	0.271	0.610	0.294	0.274	0.220	0.466	0.215
Re	0.208	0.251	0.181	0.359	0.125	0.132	0.173	0.184	0.149
Os	0.288	0.330	0.262	0.295	0.207	0.213	0.253	0.116	0.229
Ir	0.290	0.332	0.264	0.306	0.209	0.215	0.255	0.127	0.231
Pt	0.248	0.291	0.222	0.359	0.166	0.173	0.213	0.183	0.189
Au	0.140	0.185	0.114	0.468	0.106	0.085	0.106	0.300	0.082
Hg	0.394	0.434	0.368	0.238	0.315	0.320	0.358	0.077	0.335
Tl	0.222	0.266	0.196	0.418	0.140	0.147	0.188	0.245	0.164
Pb	0.262	0.306	0.236	0.393	0.181	0.187	0.228	0.217	0.204
Bi	0.220	0.264	0.194	0.443	0.138	0.145	0.186	0.271	0.162

Supplementary Data- continued

	Hf	Ta	W	Re	Os	Ir	Pt	Au	Hg
H	3.861	4.331	8.644	3.343	3.048	3.064	3.251	3.897	2.811
D	1.044	0.991	1.130	1.133	1.206	1.202	1.155	1.042	1.286
He	1.351	1.306	1.144	1.425	1.482	1.479	1.443	1.352	1.542
Li	2.575	2.680	3.206	2.444	2.356	2.362	2.419	2.590	2.280
Be	0.901	0.956	1.129	0.982	1.057	1.054	1.007	0.914	1.144
B	1.055	1.004	0.917	1.144	1.214	1.211	1.168	1.061	1.293
C	0.920	0.868	1.005	1.013	1.087	1.085	1.039	0.928	1.173
N	0.920	0.976	1.144	0.838	0.898	0.895	0.848	0.939	0.990
O	0.940	0.890	0.893	1.034	1.106	1.104	1.060	0.952	1.191
F	0.931	0.880	0.859	1.024	1.097	1.095	1.052	0.944	1.181
Ne	1.012	0.963	0.793	1.101	1.169	1.168	1.128	1.027	1.249
Na	1.094	1.049	0.886	1.178	1.240	1.240	1.204	1.111	1.313
Mg	0.892	0.843	0.771	0.987	1.059	1.057	1.015	0.910	1.144
Al	1.075	1.032	0.871	1.159	1.220	1.220	1.186	1.095	1.293
Si	0.974	0.928	0.761	1.064	1.130	1.130	1.092	0.994	1.209
P	0.855	0.807	0.690	0.951	1.022	1.022	0.981	0.877	1.109
S	1.096	1.057	0.906	1.176	1.232	1.232	1.202	1.120	1.300
Cl	0.727	0.784	0.953	0.651	0.690	0.689	0.643	0.759	0.789
Ar	1.203	1.171	1.045	1.269	1.314	1.316	1.293	1.229	1.371
K	0.937	0.893	0.731	1.025	1.089	1.090	1.055	0.963	1.168
Ca	0.807	0.760	0.591	0.903	0.973	0.973	0.934	0.834	1.060
Sc	0.778	0.833	0.991	0.706	0.636	0.645	0.699	0.814	0.600
Ti	3.145	3.517	6.377	2.740	2.498	2.516	2.676	3.205	2.312
V	1.620	1.638	1.697	1.617	1.609	1.615	1.628	1.655	1.616
Cr	0.859	0.816	0.654	0.949	1.013	1.014	0.980	0.889	1.093
Mn	3.364	3.836	8.097	2.864	2.575	2.596	2.785	3.432	2.356
Fe	0.571	0.630	0.801	0.499	0.552	0.553	0.508	0.611	0.656
Co	0.966	0.930	0.788	1.045	1.098	1.100	1.074	0.998	1.167
Ni	0.584	0.643	0.810	0.513	0.481	0.481	0.507	0.626	0.586
Cu	0.427	0.487	0.664	0.530	0.610	0.610	0.567	0.469	0.712
Zn	0.563	0.515	0.501	0.665	0.740	0.741	0.701	0.596	0.836
Ga	0.426	0.430	0.609	0.531	0.610	0.611	0.568	0.459	0.712
Ge	0.415	0.475	0.651	0.460	0.541	0.541	0.498	0.459	0.644
As	0.450	0.402	0.534	0.555	0.633	0.634	0.592	0.485	0.733
Se	0.376	0.436	0.613	0.448	0.528	0.529	0.486	0.420	0.632
Br	0.408	0.360	0.524	0.514	0.593	0.594	0.552	0.444	0.694
Kr	0.340	0.401	0.578	0.433	0.513	0.514	0.471	0.386	0.617
Rb	0.362	0.340	0.520	0.469	0.548	0.549	0.507	0.399	0.651
Sr	0.355	0.323	0.503	0.462	0.541	0.542	0.500	0.392	0.644
Y	0.301	0.361	0.539	0.401	0.482	0.483	0.440	0.347	0.586
Zr	0.322	0.310	0.490	0.429	0.509	0.510	0.468	0.360	0.612
Nb	0.318	0.291	0.471	0.425	0.505	0.506	0.464	0.356	0.608
Mo	0.331	0.284	0.436	0.438	0.517	0.518	0.477	0.370	0.619
Tc	0.314	0.267	0.431	0.421	0.500	0.502	0.460	0.353	0.603
Ru	0.287	0.256	0.437	0.394	0.474	0.475	0.433	0.325	0.577
Rh	0.365	0.318	0.339	0.470	0.547	0.549	0.509	0.404	0.647
Pd	0.352	0.305	0.331	0.457	0.534	0.536	0.496	0.391	0.634
Ag	0.341	0.294	0.322	0.446	0.523	0.525	0.485	0.380	0.624
Cd	0.425	0.380	0.214	0.527	0.600	0.602	0.565	0.465	0.695
In	0.499	0.456	0.292	0.597	0.665	0.668	0.633	0.539	0.755
Sn	0.286	0.239	0.317	0.393	0.471	0.473	0.432	0.326	0.573
Sb	0.331	0.285	0.252	0.436	0.512	0.514	0.475	0.371	0.611
Te	0.302	0.255	0.263	0.408	0.484	0.486	0.447	0.342	0.585

Supplementary Data- continued

	Hf	Ta	W	Re	Os	Ir	Pt	Au	Hg
I	0.338	0.292	0.207	0.443	0.517	0.520	0.481	0.379	0.615
Xe	0.363	0.318	0.162	0.466	0.539	0.542	0.504	0.404	0.635
Cs	0.308	0.261	0.202	0.412	0.488	0.490	0.451	0.348	0.587
Ba	0.331	0.285	0.160	0.434	0.509	0.511	0.473	0.372	0.606
La	0.150	0.211	0.388	0.191	0.273	0.275	0.232	0.200	0.381
Ce	0.336	0.291	0.123	0.438	0.511	0.514	0.477	0.377	0.607
Pr	0.353	0.309	0.142	0.455	0.526	0.529	0.493	0.395	0.621
Nd	0.092	0.151	0.330	0.201	0.281	0.283	0.241	0.141	0.388
Pm	0.324	0.381	0.540	0.259	0.191	0.202	0.257	0.374	0.138
Sm	0.886	0.874	0.806	0.935	0.962	0.968	0.960	0.931	1.008
Eu	0.099	0.095	0.276	0.208	0.288	0.290	0.248	0.140	0.394
Gd	0.143	0.096	0.217	0.251	0.330	0.332	0.291	0.185	0.434
Tb	0.072	0.091	0.271	0.181	0.262	0.264	0.222	0.114	0.368
Dy	0.417	0.470	0.610	0.359	0.295	0.306	0.359	0.468	0.238
Ho	0.056	0.117	0.294	0.125	0.207	0.209	0.166	0.106	0.315
Er	0.034	0.095	0.274	0.132	0.213	0.215	0.173	0.085	0.320
Tm	0.064	0.040	0.220	0.173	0.253	0.255	0.213	0.106	0.358
Yb	0.249	0.306	0.466	0.184	0.116	0.127	0.183	0.300	0.077
Lu	0.040	0.035	0.215	0.149	0.229	0.231	0.189	0.082	0.335
Hf	0.000	0.061	0.240	0.109	0.190	0.192	0.150	0.051	0.297
Ta	0.061	0.000	0.181	0.156	0.235	0.237	0.196	0.089	0.341
W	0.240	0.181	0.000	0.315	0.389	0.391	0.354	0.254	0.485
Re	0.109	0.156	0.315	0.000	0.082	0.084	0.041	0.119	0.192
Os	0.190	0.235	0.389	0.082	0.000	0.011	0.067	0.187	0.111
Ir	0.192	0.237	0.391	0.084	0.011	0.000	0.056	0.176	0.109
Pt	0.150	0.196	0.354	0.041	0.067	0.056	0.000	0.121	0.151
Au	0.051	0.089	0.254	0.119	0.187	0.176	0.121	0.000	0.255
Hg	0.297	0.341	0.485	0.192	0.111	0.109	0.151	0.255	0.000
Tl	0.124	0.171	0.332	0.062	0.131	0.119	0.064	0.082	0.189
Pb	0.165	0.211	0.370	0.056	0.102	0.091	0.035	0.123	0.161
Bi	0.122	0.169	0.332	0.088	0.157	0.146	0.090	0.081	0.214

Supplementary Data- continued

	Tl	Pb	Bi
H	3.460	3.296	3.536
D	1.112	1.146	1.099
He	1.410	1.438	1.400
Li	2.481	2.436	2.503
Be	0.965	1.001	0.954
B	1.131	1.164	1.120
C	1.000	1.036	0.989
N	0.873	0.845	0.891
O	1.024	1.059	1.015
F	1.016	1.051	1.007
Ne	1.096	1.129	1.088
Na	1.176	1.206	1.169
Mg	0.982	1.017	0.974
Al	1.159	1.190	1.153
Si	1.063	1.096	1.057
P	0.950	0.985	0.943
S	1.180	1.209	1.176
Cl	0.697	0.666	0.718
Ar	1.279	1.303	1.278
K	1.030	1.062	1.026
Ca	0.906	0.941	0.901
Sc	0.755	0.725	0.777
Ti	2.861	2.729	2.931
V	1.649	1.647	1.660
Cr	0.957	0.990	0.954
Mn	3.007	2.847	3.091
Fe	0.551	0.521	0.574
Co	1.058	1.086	1.057
Ni	0.566	0.536	0.589
Cu	0.537	0.576	0.532
Zn	0.674	0.711	0.670
Ga	0.539	0.578	0.535
Ge	0.468	0.508	0.464
As	0.564	0.603	0.560
Se	0.457	0.496	0.453
Br	0.524	0.563	0.520
Kr	0.443	0.482	0.439
Rb	0.479	0.518	0.476
Sr	0.473	0.512	0.469
Y	0.412	0.452	0.409
Zr	0.440	0.480	0.437
Nb	0.437	0.476	0.434
Mo	0.450	0.489	0.447
Tc	0.433	0.473	0.430
Ru	0.406	0.446	0.404
Rh	0.483	0.522	0.481
Pd	0.471	0.509	0.468
Ag	0.460	0.499	0.457
Cd	0.542	0.579	0.540
In	0.613	0.648	0.612
Sn	0.407	0.446	0.404
Sb	0.451	0.489	0.449
Te	0.422	0.461	0.420

Supplementary Data- continued

	Tl	Pb	Bi
I	0.458	0.496	0.456
Xe	0.482	0.519	0.480
Cs	0.428	0.466	0.426
Ba	0.450	0.488	0.449
La	0.205	0.246	0.203
Ce	0.455	0.492	0.453
Pr	0.471	0.508	0.470
Nd	0.215	0.255	0.213
Pm	0.320	0.292	0.345
Sm	0.966	0.982	0.973
Eu	0.222	0.262	0.220
Gd	0.266	0.306	0.264
Tb	0.196	0.236	0.194
Dy	0.418	0.393	0.443
Ho	0.140	0.181	0.138
Er	0.147	0.187	0.145
Tm	0.188	0.228	0.186
Yb	0.245	0.217	0.271
Lu	0.164	0.204	0.162
Hf	0.124	0.165	0.122
Ta	0.171	0.211	0.169
W	0.332	0.370	0.332
Re	0.062	0.056	0.088
Os	0.131	0.102	0.157
Ir	0.119	0.091	0.146
Pt	0.064	0.035	0.090
Au	0.082	0.123	0.081
Hg	0.189	0.161	0.214
Tl	0.000	0.041	0.026
Pb	0.041	0.000	0.055
Bi	0.026	0.055	0.000

Supplementary References

1. Massiot, D. *et al.* Modelling one- and two-dimensional solid-state NMR spectra. *Magn. Reson. Chem.* **40**, 70–76 (2002).
2. Mastikhin, V. M., Lapina, O. B. & Maximovskaya, R. I. ^{95}Mo solid-state NMR spectra of molybdates. *Chem. Phys. Lett.* **148**, 413–416 (1988).
3. Forgeron, M. A. M. & Wasylishen, R. E. A solid-state ^{95}Mo NMR and computational investigation of dodecahedral and square antiprismatic octacyanomolybdate(IV) anions: Is the point-charge approximation an accurate probe of local symmetry? *J. Am. Chem. Soc.* **128**, 7817–7827 (2006).
4. De Lacaillerie, J. B. D. E. *et al.* ^{95}Mo magic angle spinning NMR at high field: Improved measurements and structural analysis of the quadrupole interaction in monomolybdates and isopolymolybdates. *J. Phys. Chem. B* **109**, 14033–14042 (2005).
5. Han, O. H. *et al.* Solid-state nuclear magnetic resonance spectroscopic investigation of hydrotreating catalysts and related materials. *Appl. Catal. A, Gen.* **98**, 195–210 (1993).
6. Bastow, T. J. ^{95}Mo NMR: hyperfine interactions in MoO_3, MoS_2, $MoSe_2$ Mo_3Se_4, $MoSi_2$ and Mo_2C. *Solid State Nucl. Magn. Reson.* **12**, 191–199 (1998).
7. Hanna, J. V. *et al.* A ^{93}Nb solid-state NMR and density functional theory study of four- and six-coordinate niobate systems. *Chem. - A Eur. J.* **16**, 3222–3239 (2010).
8. Papulovskiy, E., Shubin, A. A., Terskikh, V. V, Pickard, C. J. & Lapina, O. B. Theoretical and experimental insights into applicability of solid-state ^{93}Nb NMR in catalysis. *Phys. Chem. Chem. Phys.* **15**, 5115 (2013).
9. Bonhomme, C. *et al.* First-Principles Calculation of NMR Parameters Using the Gauge Including Projector Augmented Wave Method: A Chemist's Point of View. *Chem. Rev.* **112**, 5733–5779 (2012).
10. Cuny, J. *et al.* Density Functional Theory Calculations of ^{95}Mo NMR Parameters in Solid-State Compounds. *ChemPhysChem* **10**, 3320–3329 (2009).
11. LCHIMAG - Vaspwiki. https://www.vasp.at/wiki/index.php/LCHIMAG.
12. Cross, J. & Newville, M. Inclusion of local structure effects in theoretical X-ray resonant scattering amplitudes using ab initio X-ray-absorption spectra calculations. *Phys. Rev. B - Condens. Matter Mater. Phys.* **58**, 11215–11225 (1998).
13. Novak, A. Hydrogen bonding in solids correlation of spectroscopic and crystallographic data. in *Large Molecules* 177–216 (1974). doi:10.1007/bfb0116438.
14. Fop, S., Dawson, J. A., Fortes, A. D., Ritter, C. & McLaughlin, A. C. Hydration and Ionic Conduction Mechanisms of Hexagonal Perovskite Derivatives. *Chem. Mater.* **33**, 4651–4660 (2021).
15. Fop, S. *et al.* High oxide ion and proton conductivity in a disordered hexagonal perovskite. *Nat. Mater.* **19**, 752–757 (2020).

16. Yashima, M. *et al.* High oxide-ion conductivity through the interstitial oxygen site in $Ba_7Nb_4MoO_{20}$-based hexagonal perovskite related oxides. *Nat. Commun.* **12**, 556 (2021).
17. Suzuki, Y. *et al.* Simultaneous Reduction of Proton Conductivity and Enhancement of Oxide-Ion Conductivity by Aliovalent Doping in $Ba_7Nb_4MoO_{20}$. *Inorg. Chem.* **61**, 7537–7545 (2022).
18. Sakuda, Y., Hester, J. R. & Yashima, M. Improved oxide-ion and lower proton conduction of hexagonal perovskite-related oxides based on $Ba_7Nb_4MoO_{20}$ by Cr^{6+} doping. *J. Ceram. Soc. Japan* **130**, 442–447 (2022).
19. Fop, S. *et al.* Oxide Ion Conductivity in the Hexagonal Perovskite Derivative $Ba_3MoNbO_{8.5}$. *J. Am. Chem. Soc.* **138**, 16764–16769 (2016).
20. Fop, S. *et al.* Investigation of the Relationship between the Structure and Conductivity of the Novel Oxide Ionic Conductor $Ba_3MoNbO_{8.5}$. *Chem. Mater.* **29**, 4146–4152 (2017).
21. Yashima, M. *et al.* Direct evidence for two-dimensional oxide-ion diffusion in the hexagonal perovskite-related oxide $Ba_3MoNbO_{8.5-\delta}$. *J. Mater. Chem. A* **7**, 13910–13916 (2019).
22. Miranda, C. D., López, C. A., Pedregosa, J. C. & Alonso, J. A. Structural origin of the enhanced ionic conductivity upon Nb doping in $Sr_{11}Mo_4O_{23}$ defective double perovskite. *Dalt. Trans.* **46**, 3934–3942 (2017).
23. Yaguchi, H., Fujii, K. & Yashima, M. A new structure family of oxide-ion conductors based on $BaGdInO_4$. *J. Mater. Chem. A* **8**, 8638–8647 (2020).
24. Li, R. *et al.* $Mo_3Nb_{14}O_{44}$: A New Li^+ Container for High-Performance Electrochemical Energy Storage. *Energy Environ. Mater.* **4**, 65–71 (2021).
25. Kuang, X. *et al.* Oxygen vacancy ordering phenomena in the mixed-conducting hexagonal perovskite $Ba_7Y_2Mn_3Ti_2O_{20}$. *Chem. Mater.* **19**, 2884–2893 (2007).
26. Gunkel, F. *et al.* Ordering and phase control in epitaxial double-perovskite catalysts for the oxygen evolution reaction. *ACS Catal.* **7**, 7029–7037 (2017).
27. Baca, M., Pigamo, A., Dubois, J. L. & Millet, J. M. M. Propane oxidation on MoVTeNbO mixed oxide catalysts: Study of the phase composition of active and selective catalysts. *Top. Catal.* **23**, 39–46 (2003).
28. Murayama, H. *et al.* Structure characterization of orthorhombic phase in MoVTeNbO catalyst by powder X-ray diffraction and XANES. *Appl. Catal. A Gen.* **318**, 137–142 (2007).
29. Roychowdhury, S. *et al.* Enhanced atomic ordering leads to high thermoelectric performance in $AgSbTe_2$. *Science (80-.).* **371**, 722–727 (2021).
30. Welzmiller, S. *et al.* Increasing Seebeck Coefficients and Thermoelectric Performance of Sn/Sb/Te and Ge/Sb/Te Materials by Cd Doping. *Adv. Electron. Mater.* **1**, 1500266 (2015).
31. Waterstrat, R. M., Kuentzler, R. & Muller, J. Structural instabilities and superconductivity in quasi-binary Mn_5Si_3-type compounds. *J. Less-Common Met.* **167**, 169–178 (1990).
32. Liu, Q. Z., Wang, H. F., Chen, F. & Wu, W. Single-crystalline transparent and conductive oxide films with the perovskite structure: Sb-doped $SrSnO_3$. *J. Appl. Phys.* **103**, 1–5 (2008).

33. Yashima, M., Omoto, K., Chen, J., Kato, H. & Xing, X. Evidence for (Bi,Pb)-O covalency in the high T C ferroelectric $PbTiO_3$-$BiFeO_3$ with large tetragonality. *Chem. Mater.* **23**, 3135–3137 (2011).
34. Forgeron, M. A. M. & Wasylishen, R. E. Molybdenum magnetic shielding and quadrupolar tensors for a series of molybdate salts: A solid-state ^{95}Mo NMR study. *Phys. Chem. Chem. Phys.* **10**, 574–581 (2008).
35. Lapina, O. B. *et al.* 93Nb NMR chemical shift scale for niobia systems. *Solid State Nucl. Magn. Reson.* **28**, 204–224 (2005).
36. Tansho, M. *et al.* Different Local Structures of Mo and Nb Polyhedra in the Oxide-Ion-Conducting Hexagonal Perovskite-Related Oxide $Ba_3MoNbO_{8.5}$ Revealed by ^{95}Mo and ^{93}Nb NMR Measurements. *J. Phys. Chem. C* **126**, 13284–13290 (2022).
37. Arnold, A. A. *et al.* Structure of $NaYF_4$ Upconverting Nanoparticles: A Multinuclear Solid-State NMR and DFT Computational Study. *J. Phys. Chem. C* **117**, 25733–25741 (2013).
38. Engelhardt, G., Kentgens, A. P. M., Koller, H. & Samoson, A. Strategies for extracting NMR parameters from ^{23}Na MAS, DOR and MQMAS spectra. A case study for $Na_4P_2O_7$. *Solid State Nucl. Magn. Reson.* **15**, 171–180 (1999).
39. Amoureux, J.-P. & Pruski, M. MQMAS NMR: Experimental Strategies. in *NMR of Quadrupolar Nuclei in Solid Materials* (eds. Wasylishen, R. E., Ashbrook, S. E. & Wimperis, S.) 143–161 (John Wiley & Sons, Ltd, 2012).
40. Amoureux, J.-P. & Fernandez, C. Triple, quintuple and higher order multiple quantum MAS NMR of quadrupolar nuclei. *Solid State Nucl. Magn. Reson.* **10**, 211–223 (1998).
41. Anupõld, T., Reinhold, A., Sarv, P. & Samoson, A. A comparison of double rotation and multi-quantum magic angle spinning spectra. *Solid State Nucl. Magn. Reson.* **13**, 87–91 (1998).
42. Tansho, M., Suehiro, T. & Shimizu, T. Constancy of the quadrupolar interaction product in nanocrystalline gallium nitride revealed by ^{71}Ga MAS NMR shift distribution. *Solid State Nucl. Magn. Reson.* **97**, 25–30 (2019).
43. Cromer, D. T. & Liberman, D. Relativistic Calculation of Anomalous Scattering Factors for X Rays. *J. Chem. Phys.* **53**, 1891–1898 (1970).
44. Sasaki, S. Numerical tables of anomalous scattering factors calculated by the Cromer and Liberman's method. *KEK Rep.* **88–14**, 1–136 (1989).
45. Adams, S. & Rao, R. P. High power lithium ion battery materials by computational design. *Phys. status solidi* **208**, 1746–1753 (2011).
