# Supplementary material for: Hidden chemical order in disordered Ba7Nb4MoO20 revealed by resonant X-ray diffraction and solid-state NMR
Source: Nat Commun. 2023 Apr 24;14:2337. doi: 10.1038/s41467-023-37802-4 (PMC10126145; doi:10.1038/s41467-023-37802-4)
Supplement: Supplementary file 3 — Description of Additional Supplementary Files [file 41467_2023_37802_MOESM3_ESM.docx]

**Description of Additional Supplementary Files**

Supplementary Data 1

Description: Full list of the Scattering Contrast Score
